# Protocol for biomodel engineering of unilevel to multilevel biological models using colored Petri nets

**DOI:** 10.1016/j.xpro.2023.102651

**Published:** 2023-12-08

**Authors:** Fei Liu, Monika Heiner, David Gilbert

**Affiliations:** 1 School of Software Engineering, South China University of Technology, Guangzhou, Guangdong 510006, P.R. China; 2 Department of Computing Science, Brandenburg University of Technology Cottbus-Senftenberg, D03013 Cottbus, Germany; 3 Department of Computing Science, Brunel University London, UB8 3PH London, UK

**Keywords:** Bioinformatics, Systems biology, Computer sciences

## Abstract

Biological systems inherently span multiple levels, which can pose challenges in spatial representation for modelers. We present a protocol that utilizes colored Petri nets to construct and analyze biological models of systems, encompassing both unilevel and multilevel scenarios. We detail a modeling workflow exploiting the PetriNuts platform comprising a set of tools linked together via common file formats. We describe steps for modeling preparation, component-level modeling and analysis, followed by system-level modeling and analysis, and model use.

## Highlights

• This protocol supports biomodel engineering of biological models
• Steps range from the preparation of both unilevel and multilevel models to their use
• Instructions for component-level and system-level modeling and analysis
• The PetriNuts platform exploits colored stochastic/continuous/hybrid Petri nets

## Abstract (editor highlights)

Publisher’s note: Undertaking any experimental protocol requires adherence to local institutional guidelines for laboratory safety and ethics.

## Graphical abstract

**Figure:**
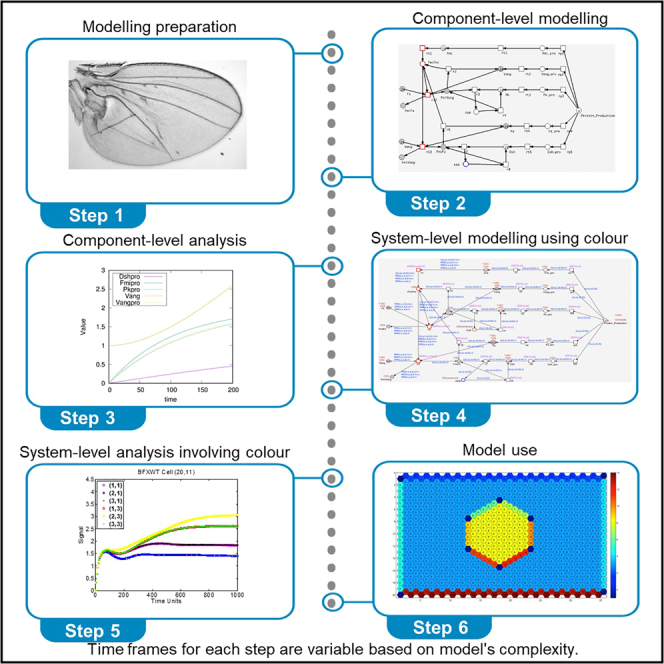

## Before you begin

### Overview

The protocol presented in this paper utilizes the PetriNuts computational platform which supports both systems and synthetic biology. Systems biology is the discipline of understanding the emergent behaviors of a biological system comprising many interacting components,^1^ as opposed to synthetic biology which is an engineering discipline concerned with the modification or construction of biological systems.^2^ Since complex biological systems are inherently multilevel, both disciplines need to incorporate appropriate mechanisms to operate from unilevel to multilevel, and this is a defining attribute of our platform.

**Figure 1:**
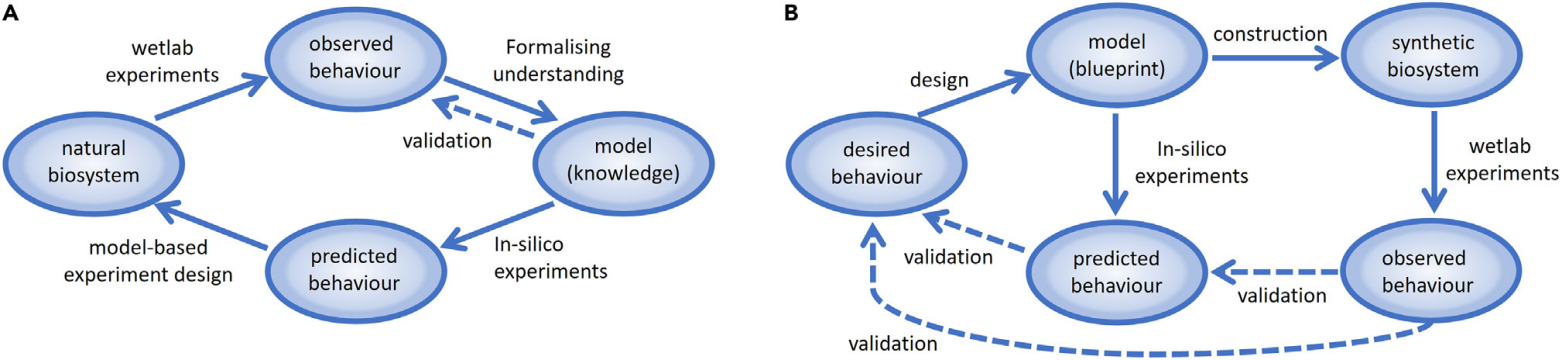
The role of models (A) Systems Biology, (B) Synthetic Biology.

Modeling, simulation and analysis play an indispensable role in both systems biology, and synthetic biology; see Figure 1. Systems biology can produce good explanations or predictions about experimental results, exploit *in silico* experiments thus saving time or cost, allow to better understand or visualize complex biological processes, and offer a whole image of a complex biological system by integrating all quantitative data and qualitative knowledge. Synthetic biology uses computational models as *designs* to guide the modification of biological systems, construction of novel biological systems or the external reconfiguration of existing biological systems by altering their environments (inputs).

We are concerned with developing dynamic models as opposed to static models because the systems that we are modeling are inherently dynamic, and we are interested in exploring, analyzing their behaviors and predicting the effect on their behaviors of changes in their environment or even modifications to their composition. A variety of dynamic modeling approaches have been proposed,^3^ to represent and analyze a wide array of biological systems, e.g., signaling pathways, regulatory networks and metabolic networks. Such approaches include e.g., ordinary differential equations (ODEs), stochastic methods, and Petri nets. Among these, Petri nets are highly suitable to represent the concurrent, asynchronous, and dynamic behavior of biological systems because of both the intuitive graphical representation, which is easily comprehended by biologists, and their powerful facilities for simulation and analysis. Section 1-3 of supplemental information summarize the basic principles for modeling using Petri nets with a focus on biosystems. Section 4 of supplemental information gives an example, the repressilator. Many extensions of Petri nets (e.g., stochastic, continuous, and hybrid Petri nets) have been applied to modeling different aspects of biological systems^4^; see Section 5-6 of supplemental information for a summary.

### From unilevel to multilevel

Currently systems biology is moving towards an emphasis on the integrated modeling of biological systems, which includes two main directions: (i) scaling up to whole-cell modeling,^5^ aiming at the construction of models for all the main components and interactions of a cell thus involving the integration of many different types of pathways, and (ii) multilevel (including multi-cellular) modeling,^6^ aiming at the construction of models consisting of different levels of a biological system. So far, whole-cell modeling is often achieved without the explicit use of levels, e.g., flattening cellular compartments into a unilevel model. However multilevel modeling explicitly considers modeling at multiple levels (e.g., subcellular, cellular, tissue and organ levels) of biological systems and integrates them into one model, which can more accurately describe a system and thus provide more insights into the system. Specifically, multilevel modeling at least can address the following biological issues: cellular morphogenesis, intercellular signaling, cell-cell communication, cell movement/chemotaxis, and pattern formation by distributing differentiated cells in appropriate three-dimensional structures in space and time.

Modeling beyond one level introduces plenty of challenges, e.g., repetition of components (e.g., cells, tissues), (hierarchical) organization, communication or movement/diffusion of components, differentiation, division or deletion of components or pattern formation of a biological system. To address these challenges, colored Petri nets (ColPNs) have been shown to be very suited for the construction of multilevel, multiscale and multidimensional models, and gained increased popularity for a wide spectrum of applications.^7^ ColPNs offer parameterized, compact graphical representations of complex biological systems with powerful simulation and analysis capabilities. With ColPNs, the locality of each species (taking cells as an example) is represented as a color, and thus the whole biological system (its structure and space) can be represented as nested tuples of colors in Cartesian coordinates, which clearly show the hierarchical two-/three- dimensional (2D/3D) organization of the system. The communication of cells is interpreted as the information exchange of colors denoting the cells, and similarly the movement of cells is described as the change of colors. The division or death of cells is simply achieved by adding new colors or removing the corresponding colors from the defined color set. In summary, ColPNs represent the structure of a biological system as a graph which is encoded using colors and convert the manipulation of species at each level or across levels into the manipulation of the graph.

**Figure 2:**
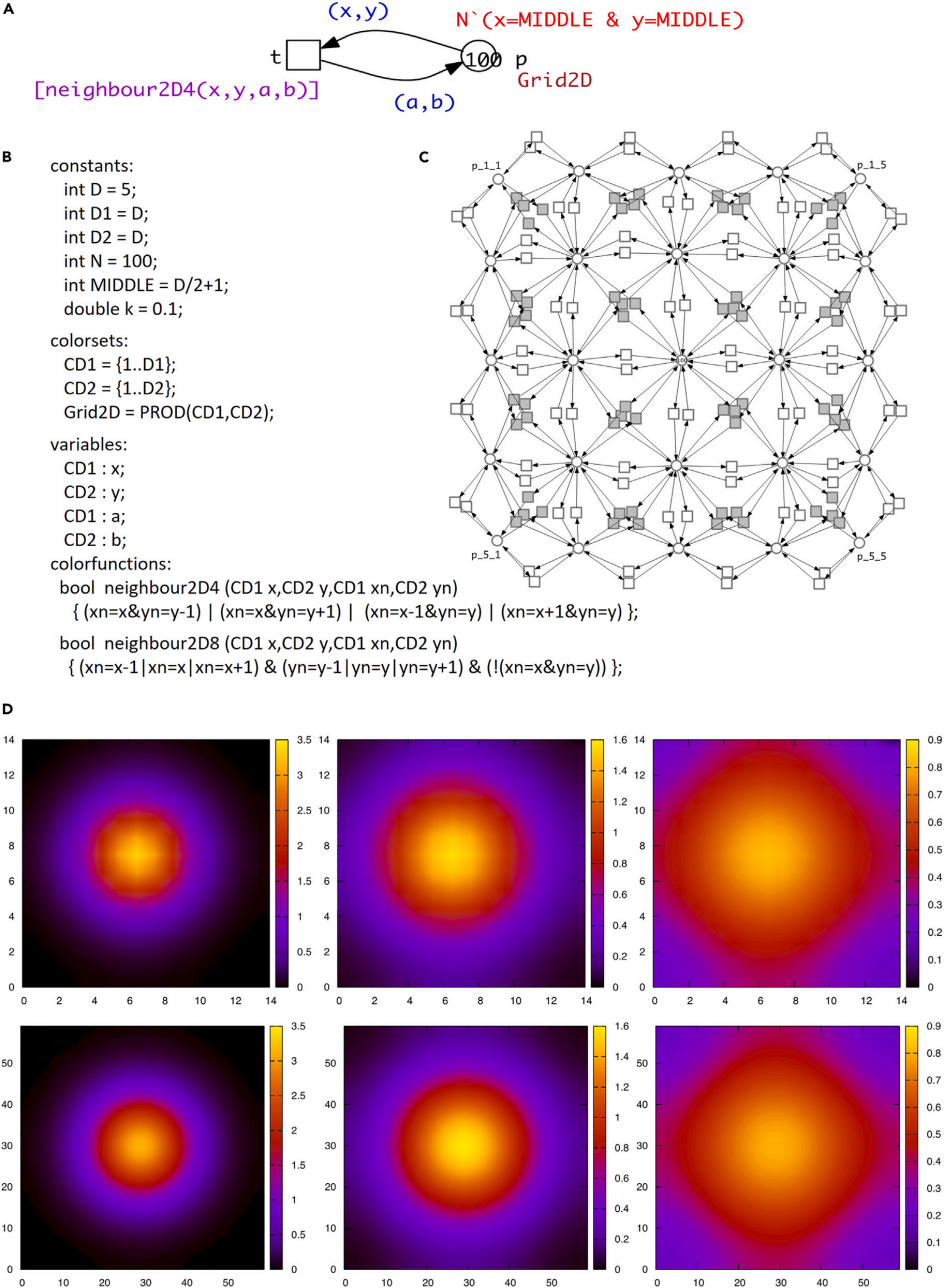
Case study: Diffusion in 2D Diffusion is a basic phenomenon occurring in many biological systems evolving in time and space. (A) Colored Petri net, which can equally be read as ColSPN or ColCPN; the firing rate of the transition t follows mass-action law with the kinetic parameter k (not shown). (B) All color-related annotations. The model is scalable by the constant D, specifying the size of the square grid. The model can be easily adjusted to different neighborhood relations by exchanging the color function used in the transition guard (given in square brackets). (C) Unfolded Petri net for D = 5 with four neighbors (white transitions only), and eight neighbors (including gray transitions). (D) 2D representation of continuous simulation results for space resolution 15 × 15 (first row) and 60 × 60 (second row); snapshots taken at simulation time 25, 50 and 100 (from left to right).

The terminology of ColPN is given in Section 7 of supplemental information, basic syntax in Section 8 of supplemental information and a few basic examples in Section 9 of supplemental information; the use of color to encode space is illustrated in Section 10 of supplemental information, and an introductory case study is given in Figure 2.

### The framework

**Figure 3:**
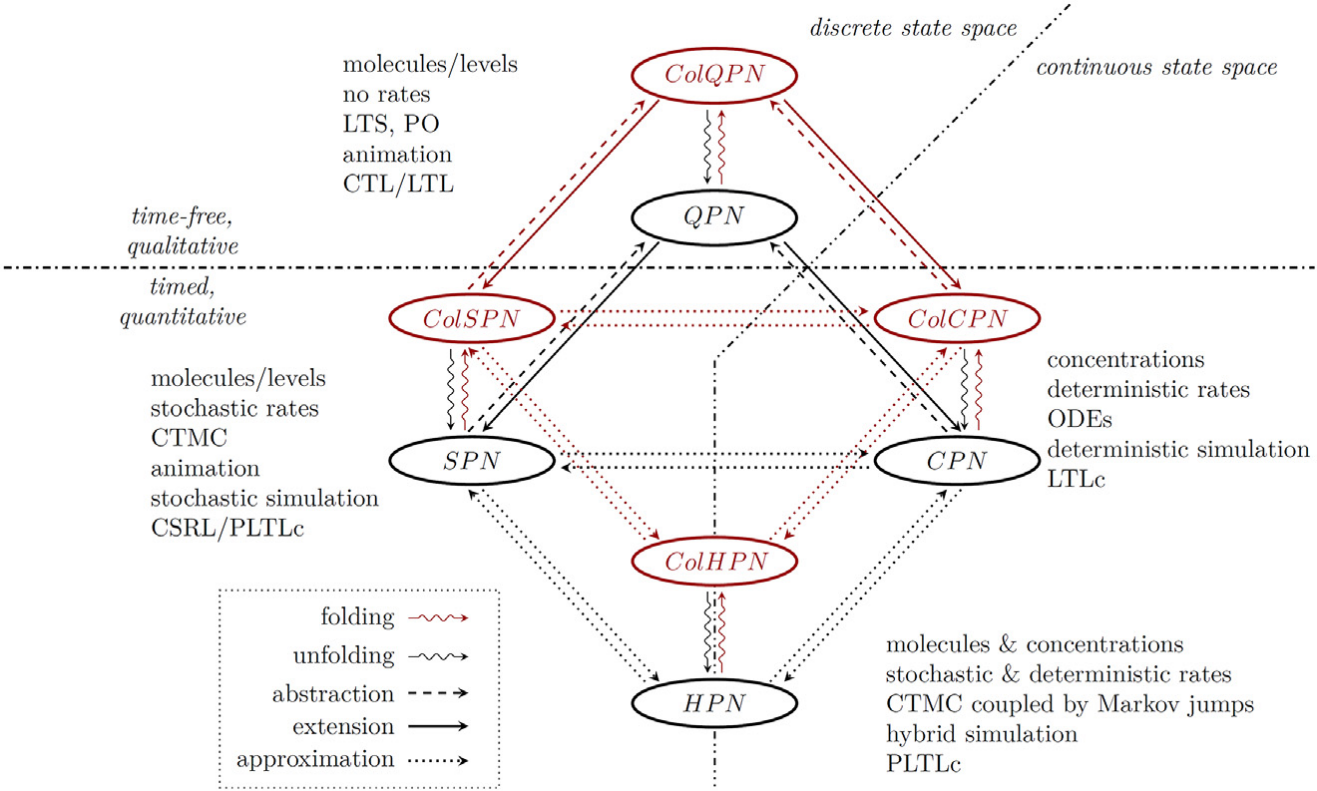
Paradigms integrated in Snoopy’s unifying Petri net framework This figure is adapted from Heiner, et al.^11^ See Section 28 of supplemental information for explanation of the abbreviations. The colored counterparts to the four uncolored net classes are given in red. All six quantitative (timed) net classes have a fuzzy counterpart; see Section 6 of supplemental information. Each paradigm is annotated by the following attributes which equally apply to the uncolored and colored net classes: (A) interpretation of the marking, (B) interpretation of the reaction rates, (C) semantics, (D) execution type: animation/simulation type, (E) temporal logic type corresponding to the given semantics to describe expected properties. A model given in a specific net class can be converted into any other net class, possibly in several steps. This conversion may involve a loss or enrichment of information, if we move between the qualitative and quantitative paradigms (see arrows abstraction and extension). The conversion may also change the graphical representation of a model (without changing the actual network), if we move between the uncolored and colored levels (see arrows folding and unfolding). The conversion may also just mean a different interpretation of the kinetic information (rates), without changing anything in the model specification, if we move between the stochastic, continuous, and hybrid paradigms (see arrows approximation). The conversion between the fuzzy and non-fuzzy (crisp) paradigms just changes which kind of kinetic parameters are accepted.^12^

**Figure 4:**
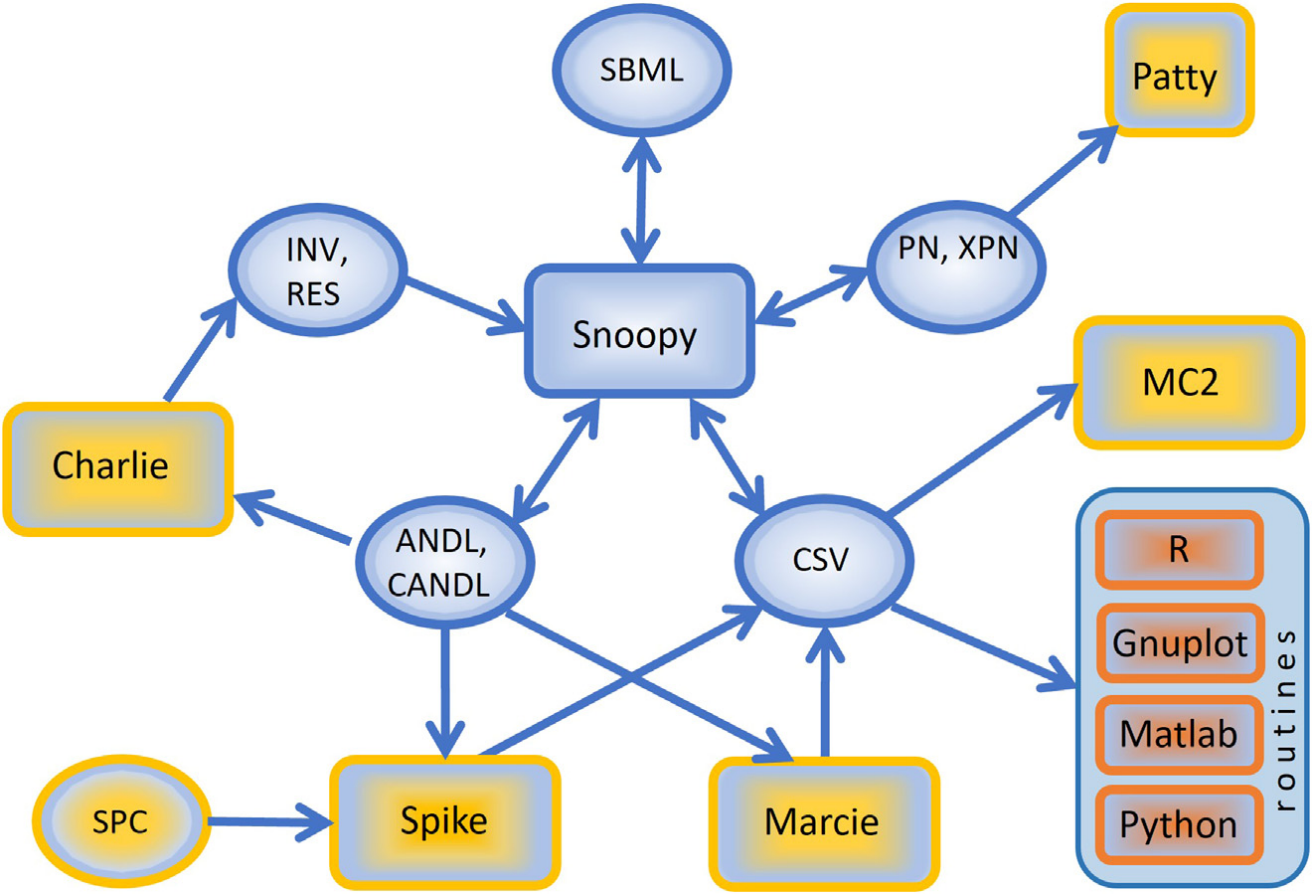
Architecture of the PetriNuts platform See Section 25 of supplemental information for a brief description of the tools, which all run independently and communicate via files. Spike also reads SBML; however, if enrichments of SBML models are required, such as adding initial concentrations or kinetic rates, models should be first read by Snoopy. Legend (see Section 28 of supplemental information for full description of abbreviations): (A) PN, XPN: Snoopy files in XML format; (B) ANDL/CANDL: Snoopy’s exchange formats as plain text; (C) CSV: Simulation traces as ‘comma separated values’ file; (D) SPC: Spike’s configuration file; (E) INV, RES: Analysis result files generated by Charlie and read by Snoopy for visualization; (F) SBML: SBML files.

Over the years, we have developed a BioModel Engineering approach,^8^ building on Petri nets for constructing and analyzing multilevel and multiscale models.^9^^,^^10^ The sound use of color enables the construction of scalable models, increasing maintainability and reusability, and also permits the exploration of size-dependent emergent behaviors. Our approach integrates a variety of non-colored and colored Petri net classes (including qualitative, stochastic, deterministic and hybrid) in a framework, see Figure 3.^11^^,^^12^ This framework is implemented in our PetriNuts platform, see Figure 4, which incorporates a set of powerful tools, see Section 25 of supplemental information, which can seamlessly achieve the modeling and analysis of a multilevel biological system. Moreover, Petri net models from different classes can be conveniently converted into each other, which enables the investigation of a biological system using various complementary modeling abstractions.

### Applications

The platform has been applied to construct and analyze many case studies ranging from unilevel to multilevel, see Section 26 of supplemental information. Amongst them are the following multilevel, multiscale and multidimensional systems (see Section 11 of supplemental information for an explanation of these terms):

1. Multilevel modeling and analysis of the phenomenon of Planar Cell Polarity (PCP) signaling in *Drosophila* wing.^13^ Highlighted features include: 2D space encoding, hierarchical organization, and pattern formation
2. Multiscale modeling of coupled *Ca*^2+^ channels.^14^ Highlighted features include: 2D space encoding, diffusion, and hierarchical organization.
3. Creating multilevel models by assembling components of biomolecules from a module-oriented database.^15^
4. Modeling 3D diffusion of calcium as a hybrid (stochastic and deterministic) model.^16^
5. Modeling and analyzing the multicellular biological system of *C. elegans* vulval development.^17^ Highlighted features include: one- dimensional (1D) space encoding, and patter formation.
6. Modeling and simulating reaction–diffusion systems.^18^ Highlighted features include: 2D space encoding, diffusion, hybrid simulation, and patter formation.
7. Spatial-temporal modeling and analysis of bacterial colonies with phase variable genes.^19^ Highlighted features include: 2D space encoding with polar coordinates, cell movement, and cell growth.
8. Modeling the humoral immune system response at the cellular scale to reproduce the adaptive response like memory and specificity features.^20^
9. Modeling of yeast cell cycles based on multisite phosphorylation.^21^ Highlighted features include: 2D space encoding, and hybrid simulation
10. Spatial modeling of complex multiscale molecular biosystems,^22^ in which various regular and irregular cellular structures can be encoded using ColPNs.
11. Analyzing the patterning of boundary cells in the *Drosophila* large intestine.^23^ Highlighted features include: 2D space encoding, hybrid simulation, and patter formation.
12. Spatial quorum sensing modeling for *E. coli* biofilm formation driven by Autoinducer 2.^24^ Highlighted features include: 3D space encoding, cell communication, diffusion, and pattern formation.

### Comparison with other methods and tools

In the computational modeling area, there are basically two classes of modeling approaches: (1) mechanism modeling (also called white-box modeling), which focuses on the mechanism of the system and is based on physical laws. Petri nets are usually considered as a mechanism modeling method. (2) Data driven modeling (also called black-box modeling), which produces models based on the inputs-outputs behavior of the system by hiding the structure of the system. In reality, we usually achieve models by the complementary use of both approaches, i.e., biological knowledge supported by datasets. The knowledge element provides the basis for constructing the topology of the model (i.e., network structure), and the datasets can thereafter provide the input for parameter fitting/tuning using standard approaches.

Petri nets are known as a popular tool in the systems biology area and are widely used for constructing and analyzing different types of biological networks.^25^^,^^26^ Standard (non-colored) Petri nets do not scale up, as also many other formalisms do not, because they do not have the appropriate abstraction mechanisms, and thus cannot be easily used for constructing and maintaining large and/or complex models. This drawback equally applies to popular Petri net tools, which usually only deal with networks at the pathway level, such as Cell Illustrator,^27^ MUFINS,^28^ CPN Tools,^29^^,^^30^ and a general modeling framework to study epidemiological systems.^31^ To address this issue, colored Petri nets have been extended to the timed, quantitative world and implemented in Snoopy and the associated tools in the platform which permits their exploitation as a parameterized modeling method for constructing large multilevel models which can include descriptions of space. For example, this high-level approach can easily simulate a model with more than 500 cells,^13^ and beyond, see Section 27 of supplemental information for more examples. Of course, the platform can be used in a basic manner to construct unilevel models and without any notion of space. Currently, our Petri net platform has been widely used by many research groups, cited more than 600 times (according to Google scholar, cites for Heiner et al.^11^), and used in many international training workshops and tutorials, see https://www-dssz.informatik.tu-cottbus.de/BME.

The key features and advantages that this approach offers as implemented in our platform, see Figure 4, are:

1. Graphical representation of multilevel and multiscale biological systems, which is easy to comprehend and use by both computer scientists and computational biologists.
2. A powerful tool for constructing and analyzing multilevel biological models. For example, the same model can be investigated under different complementary modeling paradigms – qualitative (no notion of time), stochastic, continuous, hybrid, possibly involving fuzzy kinetic parameters, and colored versions of all of these, see Figure 3.
3. Each paradigm comes with a set of simulation algorithms which can be run either in the graphical mode with Snoopy or in the command line mode with Spike (much more efficiently), which with the ability to perform sophisticated scanning facilitates the simulation of large multilevel models.
4. Formal analysis methods, e.g., structural analysis techniques, analytical and simulative model checking, for analyzing multilevel models, assuring the validity of the constructed models.
5. The ability to construct large models of multilevel biological systems in a concise manner by the use of colors to encode levels, space and repeated entities. These colored descriptions can represent very large models when unfolded, comprising many thousands of chemical reactions and biochemicals, see Section 27 of supplemental information for some examples. Our platform can simulate and analyze these huge models.
6. The ability to construct scalable models, contributing to maintainability and reusability; see Figure 2 for an example.

### Use of the protocol in BioModel Engineering

The protocol presented enables scientists to carry out BioModel Engineering, supported by our platform. BioModel Engineering is the science of systematically designing, constructing and analyzing computational models of biological systems.^8^ It takes place at the interface of computing science, mathematics, engineering and biology, and is inspired by concepts from computing science and efficient software engineering strategies. BioModel Engineering does not aim at engineering biological systems per se, but rather aims at describing their structure and behavior, in particular at the level of intracellular molecular processes, using computational tools and techniques in a principled way.^9^^,^^10^

**Figure 5:**
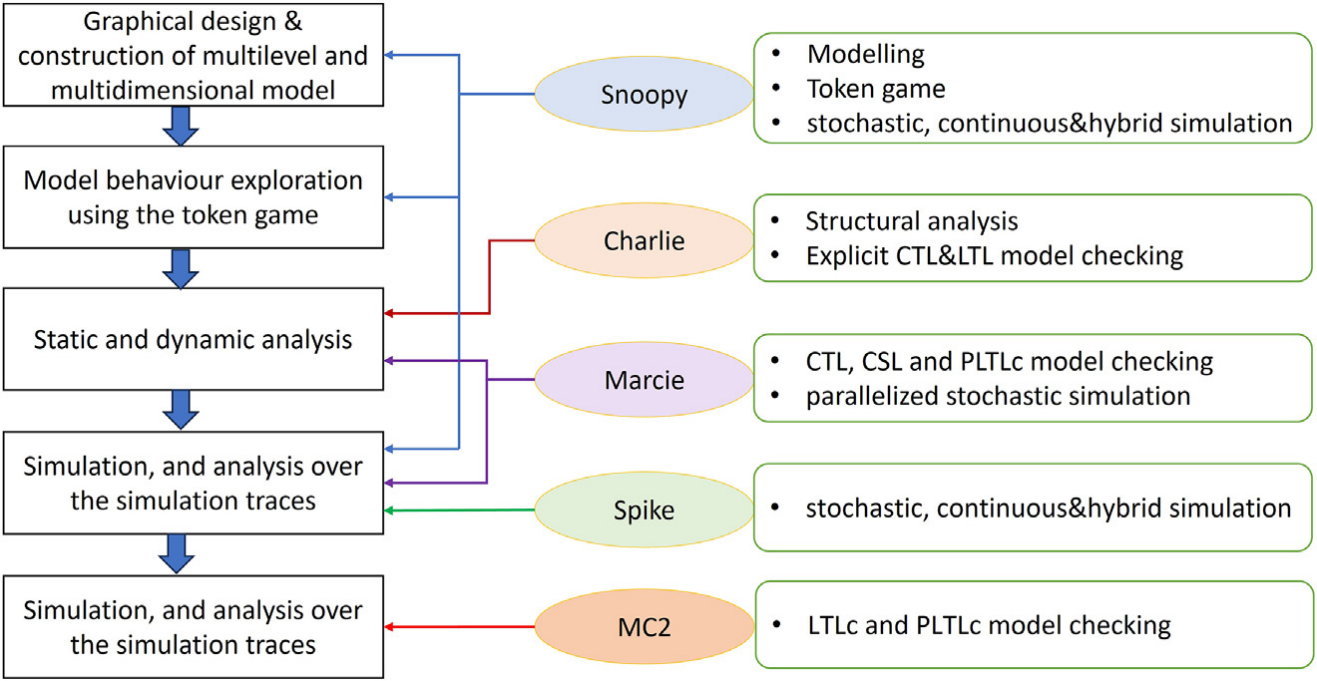
Modeling stages with corresponding tool support by the PetriNuts platform

Our protocol can be thought of as a workflow for the systematic development of models of biological systems (‘biomodels’ for short), and incorporates the following stages (compare Figure 5):

1. Graphical design & construction of multilevel and multidimensional models, supported by sophisticated color mechanisms, while multiscale models are facilitated by hybrid nets (Snoopy).
2. Model behavior exploration using the token game (token animation) (Snoopy).
3. Static and dynamic analysis as offered by Petri net theory, such as place and transition invariants, complemented by analytical model checking (Charlie & Marcie).
4. Simulation, and analysis over the simulation traces, e.g., by simulative model checking and methods from data analytics (Snoopy, Marcie, Spike, MC2).
5. Parameter fitting via scanning (Spike).
6. Optimization (follow standard approaches).

The use of color greatly facilitates maintenance of complex models which contain repeated elements, and also enables scalability with the appropriate use of constants when defining color set upper bounds.

### Expertise needed

This protocol can be used by researchers working in the area of modeling and simulation of biological systems. These can include computer scientists, computational biologists, epidemiologists, life and biomedical scientists, bioengineers and mathematicians.

Developing colored models is much closer to programming than for uncolored models and requires careful and stepwise model development.

### Hardware and software

A standard computer with a Windows, Mac or Linux operating system (preferably 64-bit) is needed. The minimum RAM required is 8 GB; depending on the size of the constructed model, more memory may be needed. More heavy simulation experiments take advantage of multiple cores.

The platform comprises several tools; see Section 25 of supplemental information. The core can be downloaded from http://www-dssz.informatik.tu-cottbus.de/DSSZ/Software. All these tools can be installed separately, are free for non-commercial use, and run on Windows, Linux, and Mac OS.

Additionally, we recommend the use of several third-party tools for post-processing of simulation traces; see also Section 25 of supplemental information.

### Basic terminology

By way of introduction we explain the basic notions of our modeling methodology: component, instance, system, and relationships among them.

The overall design of a system should be top-down, and implementation of the system is bottom up and can be performed iteratively by the stepwise composition of components to obtain the next higher level in the system hierarchy. The components themselves have to be developed and tested before their integration, and can be developed independently and in parallel within a collaborative teamwork environment. Components should be stored in libraries to facilitate reuse. Note that if there is only a single (space free) component, the introduction of space into a model introduces at least two levels.

We distinguish here between components and instances of components. A **model component** is a building block (template, pattern) which can be stored in a library. An **instance** is the use of a component, produced by importing the component from a library. A component can be replicated into several instances for inclusion in a larger model, using either copy and paste or preferably color. Instances can possibly be variants, with the same structure but with different rates and/or markings.

A **system model** comprises instances from one or more components, which includes in the simplest case just one instance from one component. A system model may be decomposed into several subsystems. Note that a system model which has been validated could be stored in a library and become a component in another model.

Correspondingly, we distinguish between relationships between components and relationships between instances.

**Relationships between components** are typically achieved by directly connecting the components by shared places and/or transitions, without using color, and no spatial information is encoded in the composition. For example, we have developed system models to describe quorum sensing and biofilm formation in bacteria^24^; an initial non-spatial subsystem was designed which combined the component producing the AI-2 signaling molecule with the quorum sensing component which also produces biofilm.

**Relationships between instances** are conveniently achieved using color, enabling spatial relationships to be modeled. There are two methods to encode space: (i) using graphs facilitating long distance relationships, (ii) using a coordinate system enabling short distance spatial relationships (typically diffusion); these methods can be combined to give powerful spatial descriptions. Continuing our quorum sensing example above, space in the form of a 2D grid was added to the basic model using color coordinates, creating instances of the subsystem, which then communicate by diffusion of AI-2 in space.

Although there is no explicit tool support for a particular system development strategy in any of the systems for BioModel Engineering that we are aware of, users are free to define and adopt specific development strategies of their choice, including the order in which components are combined. Sound engineering principles require that the chosen development strategy is well documented, see Gilbert et al.^24^ for an example.

Included in the whole development process are model analysis and model checking, which may result in the need to edit components, subsystems or the whole system model. Such editing can be carried out manually or automatically.^32^

### The workflow

The procedure presented in this paper is encapsulated in the workflow described in this section which supports multilevel modeling, and is enabled by the software platform that we have developed.

**Figure 6:**
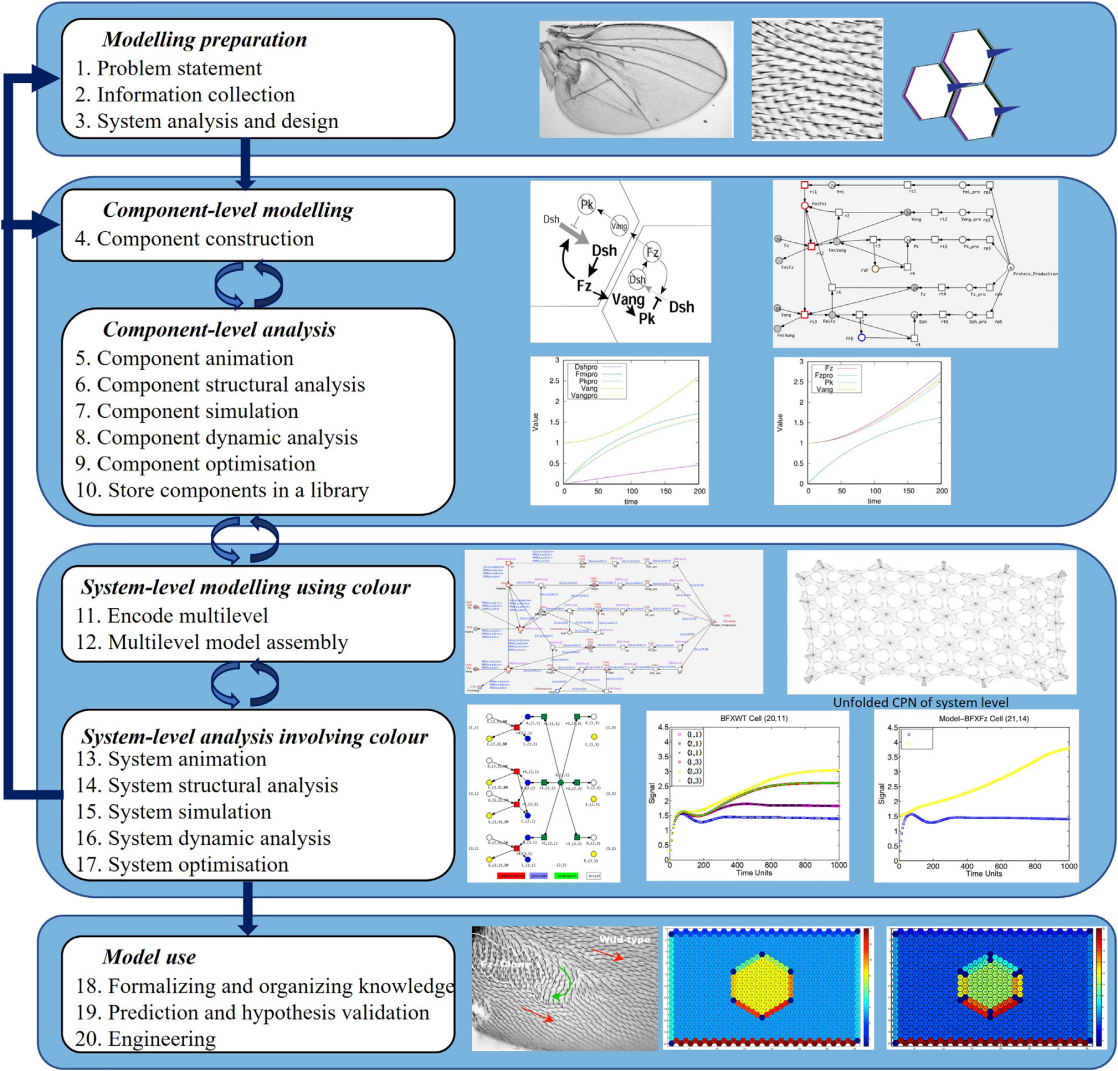
The workflow illustrated by the fly wing case study The workflow has repetitive parts indicated by the backward arrows, and thus stages can be repeated. Some sub-figures are reused from Gao et al.^13^^,^^33^ ([2013] IEEE. Reprinted, with permission, from Q. Gao, et al.^13^).

The workflow is shown in Figure 6 where the arrows reflect the system development strategy and is described in detail below. The workflow has repetitive parts indicated by the backward arrows, and thus stages can be repeated; timings below are given for each time a stage is undertaken. Typically, in the first round, components are uncolored and color is introduced at the system level when creating instances of components. In the following description of the workflow we deal with uncolored models at the component level, and introduce color concepts at the system level, and in the case of analogous steps, we only give the differences. Due to the very nature of the approach, we give more details of the procedures needed to construct components, and a higher-level view of the formation of systems from component instances.

We illustrate our workflow with two running examples: the Delta Notch pathway, introduced in Section 12 of supplemental information, and the Repressilator, introduced in Section 4 of supplemental information.

## Key resources table

**Table:** 

REAGENT or RESOURCE	SOURCE	IDENTIFIER
**Deposited data**		

2D diffusion model	BTU DSSZ	https://github.com/PetriNuts/MultilevelModelling/tree/master/snoopy_models/Diffusion
Repressilator model	BTU DSSZ	https://github.com/PetriNuts/MultilevelModelling/tree/master/snoopy_models/Repressilator
Delta Notch model	BTU DSSZ	https://github.com/PetriNuts/MultilevelModelling/tree/master/snoopy_models/DeltaNotch

**Software and algorithms**		

Snoopy	BTU DSSZ	https://www-dssz.informatik.tu-cottbus.de/DSSZ/Software/Snoopy
Charlie	BTU DSSZ	https://www-dssz.informatik.tu-cottbus.de/DSSZ/Software/Charlie
Marcie	BTU DSSZ	https://www-dssz.informatik.tu-cottbus.de/DSSZ/Software/Marcie
Spike	BTU DSSZ	https://www-dssz.informatik.tu-cottbus.de/DSSZ/Software/Spike
Patty	BTU DSSZ	https://www-dssz.informatik.tu-cottbus.de/DSSZ/Software/Snoopy#webanimation
MC2	BTU DSSZ	https://www-dssz.informatik.tu-cottbus.de/DSSZ/Software/MC2
R routines	The R Foundation	https://www.r-project.org
Gnuplot routines	SourceForge	http://www.gnuplot.info/
MATLAB routines	MathWorks	https://www.mathworks.com/
Python routines	Python Software Foundation	https://www.python.org/
2D plot Python routine (for hexagonal arrangement)	BTU DSSZ	https://github.com/PetriNuts/MultilevelModelling/tree/master/third_party_routines/2DHexagonPlot
2D movie generation Python routine	BTU DSSZ	https://github.com/PetriNuts/MultilevelModelling/tree/master/third_party_routines/2DMovie

**Other**		

Movies of 2D diffusion	BTU DSSZ	https://github.com/PetriNuts/MultilevelModelling/blob/master/video_clips/2D_diffusion%20movies.zip
Fuzzy colored Petri nets (repressilator example)	BTU DSSZ	https://github.com/PetriNuts/MultilevelModelling/blob/master/video_clips/Repressilator-fuzzybands-membership.wmv

## Step-by-step method details

### Modeling preparation

**Timing: hours to months based on model's complexity**

This part describes the recommended steps before starting the actual model development.

1. Problem statement.
  a. Provide a clear statement of the biological problem to be studied and the purpose for developing the multilevel model.
  b. Determine the system boundaries, i.e., what parts should be considered in the model.
  c. Identify the levels to be considered (e.g., cell, tissue and organ) and what types, if any, of spatial relationships are required to describe the system.
  d. Create a detailed document *Problem Description & Requirements Specification* for recording all of these, including a set of requirements regarding the expected behavior at the levels of both components and system.
2. Information collection about the biological system to be studied.

***Note:*** According to the purpose of the modeling and identified problem domain, collect relevant information including spatial relationships from the literature and databases for inclusion in the multilevel model.

3. System analysis and design.
  ***Note:*** Considering the purpose of the modeling and the information available, perform analysis on the biological system to determine the main model components required, their instances at each level, their interactions within and between levels, and their specific spatial relationships.
  a. Determine the relationship between components.
    ***Note:*** Determine the structure of the model in terms of the number and name of levels, the components at each level and the connection of components between levels. Often the component structure will be strictly linear, but can also be a tree or more generally an acyclic graph. For example, considering the modeling of a *Drosophila* fly wing tissue,^13^ there are four levels, each containing one component: tissue, cell, compartment and biochemical reaction network. In our model of bacterial quorum sensing,^24^ there are three levels: bacterial colony, bacterium, and biochemical reaction network; the latter comprises two components (AI-2 production, and AI-2 detection triggering biofilm formation).
  b. Determine the relationship between instances.
    ***Note:*** Determine the organization of the instances within and between levels, introducing the notion of spatial relationships. For example, considering a multicellular organism, at the tissue level, cell instances are organized into regular or irregular patterns in one-, two-, or three-dimensional space. At the cell level each cell generally contains instances from several intra-cellular components, for example cytosol, mitochondrion and nucleus in eukaryotes. There is only one instance of the cytosol and the nucleus, but there are several instances of the mitochondrion - the mitochondria. At the intra-cellular level, each instance of a component contains a network of biochemical reactions. The *Drosophila* fly wing case study presents a tissue model comprising tightly packed hexagonal cell instances, the internal structure of which is modeled by several virtual compartments.^13^ In the quorum sensing example,^24^ the two components are instantiated in a Cartesian two-dimensional space.

### Component-level modeling

**Timing: hours to days based on model's complexity**

This part describes the options for obtaining a Petri net model for each component (e.g., a compartment or a cell) of the multilevel model. Obtaining each model component can be realized independently and in parallel within a collaborative team.

**Figure 7:**
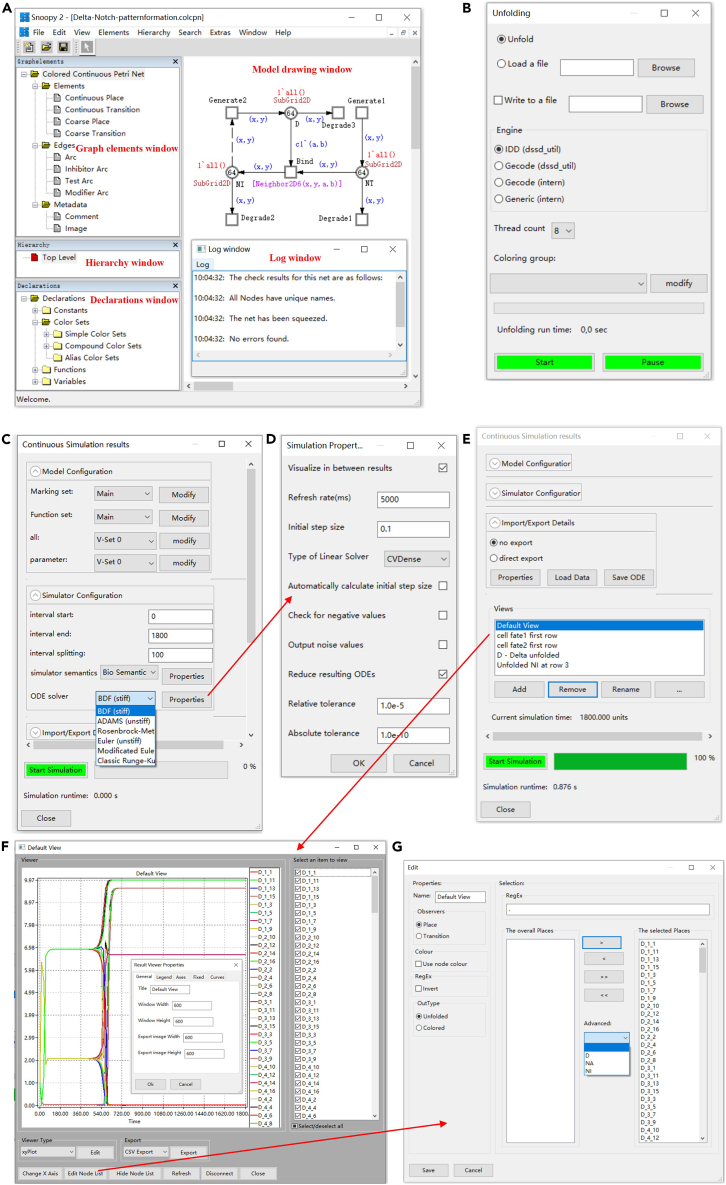
Modeling and simulation interface of Snoopy (A) Snoopy’s main interface, with which you can select a modeling element (left upper tree), display the hierarchical net organization (left middle tree), declare constants and colors (left lower tree), and check the Log window for any syntax issues. (B) The unfolding dialog, with which you can choose an unfolding engine and the number of threads to be used for unfolding. (C) The simulation dialog, with which you can configure the model involving the marking set, function set and parameter set, and configure the simulator including simulator semantics and solver, e.g., (D) gives the simulation properties of the chosen BDF solver. (E) The simulation dialog, with which you can manage the import and export of data, and manipulate the views to show simulation results. (F) Display of simulation results, where simulation traces (views) can be selected, configured and exported. (G) Node selection dialog, which appears by clicking the ‘Edit Node List’ button in (F).
    ii. Select the ‘Import’ item in the ‘File’ menu and pop up the ‘Import’ dialog.
    iii. On the ‘Import’ dialog, choose ‘Import SBML to discrete/continuous/stochastic Petri net’ and then click the ‘OK’ button.
  b. Reuse, and if required modify, an existing model available in the Snoopy format.
    ***Note:*** Similarly, semantic error checking should be carefully performed. This can be simply done by opening a net in Snoopy by clicking the ‘Open’ button.
  c. Construct a new model from scratch**.** See Figure 8 for an illustrative example (see the Delta Notch model in key resources table).
    i. Create a new Petri net file.
      ***Note:*** Open the Snoopy software (see Figure 7 for the main interface of Snoopy), click the ‘New’ button and choose a Petri net type (e.g., stochastic Petri net). See Table 1 for some guidelines how to choose a suitable net class and Section 5 of supplemental information for more detailed information. Then a new drawing window (canvas) appears, corresponding to the chosen Petri net type.

4. Component construction.
  ***Note:*** This can be done in either of three ways (see Section 13-15 of supplemental information for software use).
  a. Import an existing SBML model from some library or other external sources.
    ***Note:*** Pre-existing models need to be checked carefully for semantic errors, for example mistakes in operations or spelling in biochemical equations. The reuse of an SBML model developed for FBA requires the addition of initial marking (concentrations) and rate functions; see Gilbert et al.^32^ for more details, including automated editing and error correction.
    i. Open the Snoopy software (see Figure 7 for the main interface of Snoopy).

**Table 1:** Guideline for choosing the right net class

Net class	Features
SPN/ColSPN	1. Support stochastic modeling; 2. Represent molecules as discrete tokens; 3. The semantics of this net class are CTMC; 4. Choose colored classes for large systems with repeated components;
CPN/ColCPN	1. Support deterministic modeling; 2. Represent the concentration species as continuous token values; 3. The semantics of this net class is a set of ODEs; 4. Choose colored classes for large systems with repeated components;
HPN/ColHPN	1. Support hybrid (namely stochastic and deterministic) modeling; 2. Represent molecules of some species as discrete tokens and other species as concentration values; 3. The semantics of this net class is the interplay of the CTMC component and the ODEs component; 4. Choose colored classes for large systems with repeated components.
    ii. Draw species as place nodes and set initial marking.
      ***Note:*** In Petri nets, places (circles in Snoopy) may represent different types of species like ions, molecules, proteins, and complexes. See Section 13 of supplemental information for the Petri nets elements. To add a new species, select the place icon and click on the appropriate location on the canvas. Then edit its properties by double clicking the icon that corresponds to the species. In the properties editing dialog, add in the ‘General’ tab the name of the species, the initial marking and give a description in the ‘Comment’ field, including any related literature references. Finally, set up the graphic properties, e.g., pen and brush colors, width and height of the node. All node attributes can be made visible/invisible by selecting/deselecting the corresponding option ‘show’.
      ***Note:*** We can specify the initial number of tokens of a place with a constant, which facilitates the exploration of a model in different initial marking configurations. To define a constant, double click the ‘Constants’ item in the ‘Declarations’ window, and the constant definition dialog appears, where one can add, delete or check a constant. When defining a new constant, specify its name, group, type (int or double) and its value in the Main value set. Additionally, further value sets can be defined, renamed and deleted. Constants are organized in groups; there are two pre-defined groups: marking and parameter, which are subsumed by the third pre-defined group ‘all’. Further groups can be defined. A model can be configured by help of these groups and value sets, before animating, analyzing or simulating it.
      **CRITICAL:** The use of constants has to obey the general syntax rules of Petri nets. Thus, which types of constants are available (int, double) depends on the net class. Discrete net classes (such as PN, SPN) permit only integer constants for arc weights and initial marking, while net classes with continuous nodes (CPN, HPN) also permit the use of double constants for arc weights and initial marking.
    iii. Draw biological reactions as transition nodes.
      ***Note:*** In Petri nets, transitions may represent chemical reactions, state shifts, transport/diffusion etc. To add a new reaction, select the transition icon and click on the appropriate location on the canvas. Then edit its properties by double clicking the icon that corresponds to the reaction. In the properties editing dialog, modify the properties in an analogous way as for the place nodes.
    iv. Define rate functions for transitions (appropriate for SPN, CPN, HPN).
      ***Note:*** Double click a transition, and then edit its properties. In the properties editing dialog, fill in a corresponding rate function in the ‘Function’ domain in the ‘Functions’ tab. See Section 5 of supplemental information for how to define rate functions for the different net classes.
      **CRITICAL:** Different predefined kinetic rate functions are available, e.g., mass action or Michaelis Menten. These rate functions can be chosen with the help of the function assistant in the ‘Functions’ tab. Besides, we recommend the definition and use of (double) constants for any kinetic parameters, as this facilitates the exploration of the model using different parameter settings defined as value sets. Moreover, we can define more than one rate function for a transition in the ‘Function overview’ dialog by clicking the ‘Add function set’ button, which will add another rate function for each transition. Thus, for each transition we can specify different forms of rate functions in different function sets.
    v. Draw edges.
      ***Note:*** Note that we use the terms ‘edge’ and ‘arc’ interchangeably. Edges connect place nodes and transition nodes and specify the relationships between these two types of nodes. To add an edge, select an appropriate edge type (e.g., standard or read arc). Click on the source node, and move the mouse pointer, while keeping the mouse button pressed, to the target node, where the mouse button is released. Alternatively, any mouse click on the way from the source to the target node will set an auxiliary point for the edge, permitting curved edges. By double clicking an arc and opening the properties editing dialog, we can specify a multiplicity and set the graphic properties for the arc. Likewise, the multiplicity permits a constant.
      **CRITICAL:** Arcs always connect nodes of different type (place, transition). The attempt to connect two places (transitions) is simply ignored. Different types of arcs play distinct roles, which should be properly chosen, e.g., correctly use read arcs and modifier arcs, see Section 3 of supplemental information.
    vi. Apply logical nodes (optional).
      ***Note:*** Logical nodes can improve the readability of a large network by reducing the crossing of links between nodes. To apply logical nodes, choose a set of nodes with an identical name. Enable the ‘Logic’ checkbox in the ‘General’ tab for this set, either by double clicking on each node to open its properties editing dialog one after the other, or by selecting the whole set, followed by ‘Edit selected elements’. After that, all these chosen nodes become gray and changing the properties of one node will immediately affect all the others.
    vii. Apply macro nodes (optional).
      ***Note:*** Macro nodes permit the structuring of a model into meaningful hierarchical subsets to reflect the physical structure of a biological system, e.g., compartmentalization of a cell.
      To apply macro nodes, first construct the net as a flat net. Then select a subnet and choose the Coarse item in the ‘Hierarchy’ menu. Select an appropriate coarse element (macro place or macro transition) and click the OK button. After that, the selected subnet will be included in a macro node. Similarly, change the name of the macro node by doubling click the node, which will open its properties editing dialog.
      **CRITICAL:** The use of logical nodes and macro nodes can greatly increase the readability of a model, and hence facilitate debugging. If appropriately applied, they should never have an influence on the underlying structure of the model, but just change the style of representation (troubleshooting 1). See Section 2 of supplemental information for more details on how to use these two kinds of nodes.
    viii. Adjust model layout.
      ***Note:*** A readable model layout is imperative to reveal the model structure and facilitates further analysis.
      In Snoopy, three automatic layout algorithms are offered -- Planarization, FMMM and Sugiyama -- which can be found in the ‘Edit/Layout’ menu. However, automatic layout may not reflect the physical structure of a component, e.g., a compartment or cell. Therefore, manual adjustment of the model layout is usually preferred. More helpful options to amend the layout can be found in the ‘Edit/Transform shapes’ menu. The layout of edges can be fine-tuned by adding auxiliary points to an existing edge by a cmd-mouse (ctrl-mouse) click.
      ***Note:*** As complementary material for component-level modeling we suggest,^24^^,^^34^ where we have illustrated in detail model construction by module composition. See also Section 26 of supplemental information for more case studies.

**Figure 8:**
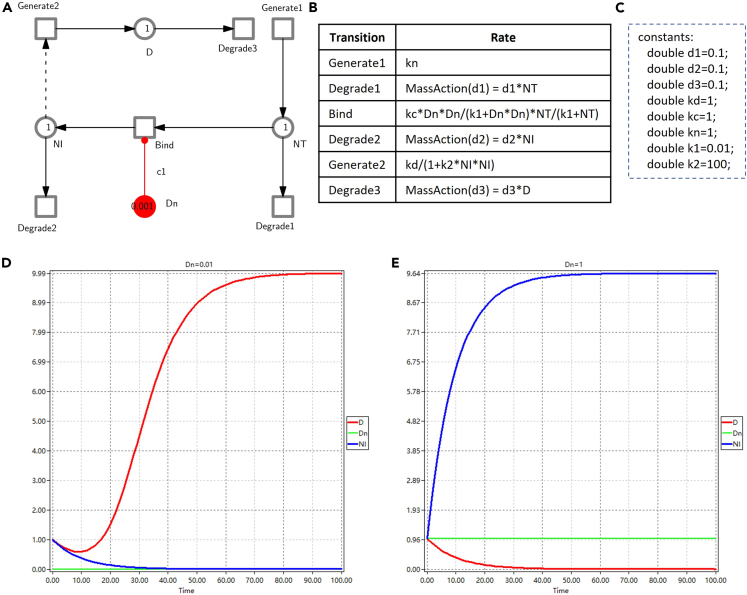
Illustration of component modeling with the Delta Notch pathway (A) A CPN model of a typical Delta Notch pathway in a single cell from a multi-cellular organism; see Section 12 of supplemental information for more details. To make this reduced component model work, we add an external entity Dn, which simulates the transmembrane ligand Delta (D) from neighboring cells. Dn is modeled as a constant stimulus. (B) The rate functions used to produce plots (D) and (E). (C) The constants used in the rate functions, adapted from Collier et al.^35^ (D and E) Two plots for Dn = 0.001 and Dn = 1, respectively. The increase of Dn (from 0.001 to 1) produces more NI, which has an inhibitory effect on the production of D in the same cell (reducing D from about 10 to 0 in the steady state).

### Component-level analysis

**Timing: hours to days based on model's complexity**

This part describes the steps for thoroughly analyzing each constructed component before instantiating them to assemble the system. Steps 5 to 9 have to consider the set of requirements contained in the Problem Statement & Requirements Specification, in order to validate the component model against them, and to modify either of them accordingly, if required.

5. **Component animation** (appropriate for PN, XPN, SPN).
  ***Note:*** Snoopy supports animation via the token game for all discrete Petri net classes to explore the model behavior by following the token flow and establish initial confidence in the model.
  a. In the ‘View’ menu, click the ‘Start Anim-mode’ item, which initiates the animation dialog.
  b. In the animation dialog, run animation and observe the animation result.
    ***Note:*** There are several options that can be selected (see Section 16 of supplemental information for details), including manual or automatic modes. In the dialog, click the ‘Play forward’ button to get an execution of the animation or repeatedly click the ‘Step forward’ button to run the animation step by step. By playing this token game, we can observe a sequence of reachable states which corresponds to a walk through the state space of a model.
6. **Component structural analysis** (appropriate for all Petri nets that do not involve special arcs (inhibitor, equal, reset and self-modifying arcs, see Section 3 of supplemental information) which bring the Turing power).
  ***Note:*** Validate the component structure using standard analysis techniques of Petri net theory by help of Charlie’s static analyses. These techniques are by their very nature exhaustive and aim at conclusions about behavioral properties, such as boundedness, liveness and reversibility (see Section 19 of supplemental information), without constructing the state space. They permit the modeler to decide if the state space is finite or infinite, and thus may guide the choice of methods applied for dynamic analyses, see Step 8. If a model is not reversible and/or not live, then the dynamic behavior observed by deterministic simulation runs and stochastic simulation runs may diverge.
  ***Note:*** Structural analysis techniques, which have proved their usefulness for BioModel Engineering, include: (a) elementary graph properties, (b) place and transition invariants, and (c) Siphon Trap Property (STP). Before performing these analyses, the Petri net model constructed in Snoopy should be exported to the ANDL format, which then can be loaded into Charlie. To perform structural analyses with Charlie, follow these steps (see also Section 18 of supplemental information).
  a. Elementary graph properties.
    ***Note:*** To decide the properties explained in Section 20 of supplemental information, click on the bar *net properties*. After loading a file, all elementary graph properties are immediately decided and the results are summarized in the result vector. Green stands for YES (the property holds) and red for NO (the property does not hold). Hovering with the mouse over a specific property reveals a tool tip giving the full name of that property. More details are given in the protocol window or the output window.
  b. **Place and transition invariants.** To compute the properties explained in Section 21 of supplemental information (first row) do:
    i. Click on the bar *IM-based analysis* (IM stands for incidence matrix).
    ii. If only structural boundedness should be decided, choose *check structural boundedness.* Alternatively, select *P-invariant* or *T-invariant.*
    iii. If the invariants shall be written to a file, go to *options, export invariants to file* and specify a target folder and file name.
      ***Note:*** By default the file name will be the same as the Petri net just loaded, extended by the suffix ‘_P.inv’ or ‘_T.inv’. Finally, close this sub-window.
    iv. Click *compute.*
      ***Note:*** The current state of the invariant analysis engine is shown in the thread manager window.
      ***Note:*** When the computation finished, the result w.r.t. CPI or CTI is shown in the result vector (the field turns from gray to red or green), and also reported in the protocol window and output window in more detail (how many invariants have been found, which places/transitions are not covered by the invariants).
    v. If the export option had been selected, all invariants have been written as plain text to the previously specified file.
      ***Note:*** This file can now be loaded with Snoopy for closer visual inspection, see Section 22 of supplemental information.
  c. **Siphon Trap Property (STP).** To compute the properties explained in Section 21 of supplemental information (second row) do:
    i. Click on the bar *siphon/trap computation.*
    ii. Select what should be computed (siphons, traps or the STP).
      ***Note:*** Do not forget to select *compute all*, if you want to obtain all siphons or traps.
    iii. If the results shall be written to a file, select *export siphons* or *export traps*, specify a target folder and file name.
      ***Note:*** By default the file name will be the same as the Petri net just loaded, extended by the suffix ‘_DLS.res’. Finally, close this sub-window.
    iv. Click *compute.*
      ***Note:*** The current state of the siphon/trap analysis engine is shown in the thread manager window.
      ***Note:*** When the computation finished, the STP result is shown (if it had been selected) in the result vector (the field turns from gray to red or green), and also reported in the protocol window and output window in more detail. At the same time any conclusions on liveness are drawn, which are now possible based on the decided structural properties, and are shown in the result vector and reported in detail in the protocol and output window (see Section 18 of supplemental information).
    v. If the export option had been selected, all place sets have been written as plain text to the previously specified file.
      ***Note:*** Like invariants, this file can now be loaded with Snoopy for closer visual inspection, see Section 22 of supplemental information.
      ***Optional:*** There is also a *place set analyzer* option, which permits the compiling of place sets, which are immediately checked whether they are trap, siphon or bad siphon.
      **CRITICAL:** The computation of place/transition invariants and the STP decision may be computationally expensive. Thus, Charlie should preferably run on a separate computer to that used for the main protocol. Place (transition) invariants may overlap, and in the worst case we get exponentially many place (transition) invariants w.r.t. the size of the Petri net. Thus, the interpretation of the subnets induced by the many invariants may be hard to manage. Then, the partitioning by ADT sets may help.^36^
7. **Component simulation** (appropriate for SPN, CPN, HPN) in order to explore the behavior of the model.
  ***Note:*** Snoopy offers a number of simulation algorithms to simulate different net classes, including SPN, CPN and HPN. To simulate a model, click the ‘Start Simulation-Mode’ item in the ‘View’ menu and the simulation dialog appears. In this dialog, you usually follow the following steps.
  a. Model configuration.
    ***Note:*** In the ‘Model configuration’ tab, choose a marking set for places, a rate function set for transitions, and a value set for every group of constants.
  b. Simulator configuration.
    i. In the ‘Simulation Configuration’ tab, set up the start and end of the simulation interval, together with number of observation points in the interval (interval splitting).
    ii. Choose an appropriate simulator type (See Table 2) and specify its setting under ‘Properties’ (see Section 17 of supplemental information).
      ***Note:*** Our platform supports two CPN semantics^37^: adaptive semantics according to David et al.^38^ and bio-semantics. The latter assumes that non-enabledness coincides with a rate of zero; thus, there is no check for enabledness, which reduces the overhead and improves the overall performance. HPN/ColHPN simulation requires advanced ODE solvers; the user can choose between ARK, BDF and ADAMS. Simulation of fuzzy Petri nets employs the simulation algorithms available for the corresponding crisp counterpart.
      **CRITICAL:** Each quantitative net class can be simulated using several simulation algorithms, each of which may generate similar, different, or even contradictory results. Therefore, the careful choice of an appropriate simulation algorithm is essential.

**Table 2:** Simulation algorithms offered by Snoopy

Net class	Simulation algorithm	Description
SPN/ColSPN	Gillespie	Gillespie’s exact SSA.^43^
	Tau-leaping	An approximate SSA.^44^
	Delta-leaping	An approximative SSA for large and dense networks,^45^ which can be used to simulate genome-scale metabolic models.^32^
	FAU	An approximative numerical analysis method.^46^
CPN/ColCPN	Euler (unstiff)	A numerical integration method for unstiff systems of ODEs, as implemented in Hindmarsh et al.^47^
	Modified Euler (unstiff)	A numerical integration method for unstiff systems of ODEs, as implemented in Hindmarsh et al.^47^
	ADAMS (unstiff)	A numerical integration method for unstiff systems of ODEs, as implemented in Hindmarsh et al.^47^
	Classic Runge-Kutta	A numerical integration method for systems of ODEs, as implemented in Hindmarsh et al.^47^
	BDF (stiff)	Backward Differentiation Formula,^48^ as implemented in Hindmarsh et al.^47^
	Rosenbrock method (stiff)	A numerical integration method of the Runge-Kutta type for stiff systems of ODEs, as implemented in Hindmarsh et al.^47^
HPN/ColHPN	Exact HRSSA	A hybrid simulation algorithm proposed by Haseltine and Rawlings.^49^
	Exact accelerated HRSSA	An improved hybrid simulation algorithm.^50^
	Improved HRSSA	A hybrid simulation method,^51^ based on exact accelerated HRSSA,^49^ and the rejection-based stochastic simulation algorithm^52^; following the ideas of Marchetti et al.^53^
	Dynamic hybrid simulation	A hybrid simulation algorithm with dynamic partitioning of the reactions.^54^
  c. Define views to show simulation results.
    ***Note:*** In the ‘Views’ tab, add a new view by clicking the ‘Add’ button, and delete a view by clicking the ‘Remove’ button. Results views need to be configured by selecting whether places or transitions are shown, and which traces are to be displayed. This can be done by clicking the ‘Edit Node List’ button and then by manual selection, or by the use of regular expressions.
  d. Perform simulation.
    ***Note:*** Perform simulation by clicking the ‘Start Simulation’ button. At the same time, a progressing bar will show the progress of the current simulation. When the simulation ends, the total simulation runtime will be given (troubleshooting 2).
  e. Observe simulation results in the view dialog.
    ***Note:*** Double clicking a view in the ‘Views’ tab, a window will appear in which one can observe traces of the selected places or transitions.
  f. Export simulation results to file.
    ***Note:*** Select simulation results of interest and export them in a CSV form with separators like colon, comma, semicolon or tabulator, or export the current plot to an (e.g., png, jpeg, gif, bmp) image. Alternatively, we can export simulation results during simulation running with the setting of ‘Import/Export details’ or load previously written data for display in a view.
8. **Component dynamic analysis**, which aims at the validation of the behavior of a component.
  ***Note:*** It complements the structural analysis in Step 6 and formalizes the evaluation of simulation traces obtained in Step 7. It can be performed in the following three ways.
  a. **Model checking**, which is used to validate the behavior of the model against desired properties.
    ***Note:*** For a general discussion on model checking and its use in the context of our platform, see Section 23 of supplemental information.
    i. Select model checker.
      ***Note:*** The following guidelines may help the user to select the appropriate tool: If the model is a bounded QPN or bounded ColQPN, then analytical model checking of CTL properties with Marcie is the recommended choice. Likewise, if the model is a bounded SPN or bounded ColSPN, then CTL model checking permits the exploration of the state space in a qualitative way. If the model is a bounded SPN or bounded ColSPN, and the state space is of moderate size, then try to use analytical model checking of CSL properties which will give exact results, if computing resources permit. Otherwise perform simulative model checking of PLTL properties with Marcie or MC2. In all other cases, simulative model checking of PLTL properties with MC2 must be used.
    ii. Perform model checking
      ***Optional:* Analytical CTL model checking** of QPN, ColQPN, SPN and ColSPN with Marcie with the following steps (for details see the Marcie Manual.^39^):
      ***Note:*** Ensure that you have the ANDL file of interest (e.g., GeneGate.andl) of a QPN (in this case the bounded gene gate model given in the key resources table) or CANDL file of a ColQPN. Write CTL queries and save them in a file (e.g., properties.ctl). Perform model checking using the following command:
      marcie --net-file = GeneGate.andl --ctl-file = properties.ctl
      and you will obtain a Boolean result: TRUE or FALSE. For example, see the property:
      AG [ [gene_b = 1 & blocked_b = 0] | [gene_b = 0 & blocked_b = 1] ]
      stands for “Forever it holds that gene b is either on or blocked”.
      ***Optional:* Analytical CSL model checking** of SPN and ColSPN with Marcie with the following steps (for details see Marcie Manual.^39^):
      ***Note:*** Ensure that you have an ANDL file of interest (e.g., GeneGate.andl) of an SPN or CANDL file of a ColSPN. Write CSL queries and save them in a file (e.g., transient.csl). Perform model checking using the following command:
      marcie --net-file = GeneGate.andl --csl-file = transient.csl --const = k = 10,t1 = 1,t2 = 1
      and you will obtain the probability of the given property. For example, see the property:
      const integer k;
      const double t1;
      const double t2;
      P = ? [ F [t1,t2] protein_b >= k ] stands for “What is the probability that there are at least k molecules of protein b between time points t1 and t2”.
      ***Optional:* Simulative PLTL model checking** of SPN and ColSPN with Marcie: with the following steps (for details see Marcie Manual.^39^):
      ***Note:*** Ensure that you have an ANDL file of interest (e.g., model.andl) of an SPN or CANDL file of a ColSPN. Write PLTL queries and save them in a file (e.g., properties.ltl). Perform model checking using the following command:
      marcie --net-file = GeneGate.andl --ltl-file = properties.ltl --const = t1 = 1,t2 = 1
      and you will obtain the probability of the given property. For example, see the property:
      const double t1;
      const double t2;
      P = ? [F [t1,t2] protein_b > $v ]
      stands for “What is the probability that the number of molecules of protein b is greater than a free variable v between time points t1 and t2”.
      ***Optional:* Simulative model checking** of all quantitative net classes using MC2 with the following steps:
      ***Note:*** First obtain a CSV data file (e.g., traces.csv) that stores the time series traces from a simulation. Write a query file (property.queries) in terms of the syntax of PLTLc (see Donaldson et al.^40^ for a detailed description). Issue the following command on the command line:
      “java -jar MC2Tool.jar traces.csv property.queries -time -snoopy”
      and you will obtain on the standard output the resulting analysis comprising the probability value for each property in the file property.queries. For example the property:
      P >=1 [ F ( G( d[A] = 0 ˆ [A] > 0 ) ) ]
      stands for ‘Eventually the concentration of metabolite A is at steady state greater than zero.’
      Note that the ‘ˆ’ symbol is the logical AND operator.
      ***Note:*** In the component model of the Delta Notch pathway given in Figure 8, we use the following query to check the relative concentrations of NI and D: P >=1[ F(G([NI] > [D] v [NI] < [D]) ) ] which reads, ‘Eventually either NI is always greater than D or vice versa’.
      **CRITICAL:** Note that there are some syntactic differences in the way in which PLTLc properties are written in Marcie and MC2. For example, properties for MC2 require the use of square brackets around each place or transition name (troubleshooting 3).^40^
  b. Time series data analytics.
    ***Note:*** The simulation output comprises time series traces of the values of place markings or transition activity, which can be saved in the form of CSV (or equivalent) files. These data can then be analyzed using standard time-series data analytic techniques using software such as MATLAB or R. The actual methods used depend on the problem being investigated; see Section 24 of supplemental information for possible options.
  c. Cross validation against data sets.
    ***Note:*** Due to the dangers of overfitting a model to data, some form of cross-validation is the standard procedure to follow. This method employs resampling, iteratively selecting subsets of the data to repeatedly test and train a model.
9. Component optimization.

***Note:*** It is often the case that although a component has the desired net structure, the behavior exhibited is not the expected one. In general, modification of the rate constants can be attempted in order to remedy this. The simplest method is that of exploration of the values of the kinetic rate constants, which can be either achieved manually, or via automated parameter scanning – a facility provided by the Spike tool and defined in the SPC file. However, the disadvantage of parameter scanning is that if the solution space is large and complex, it will be hard to ensure that the scanned ranges will cover the possible solution, and indeed there may be more than one solution.

***Note:*** Target driven optimization is an approach that can be employed in this situation; it is a heuristic and thus not guaranteed to find the actual solution, but the best solution in a reasonable time. Optimization algorithms that can be used include: hill climbing, restart hill climbing, simulated annealing and genetic algorithms,^41^ and has been used in e.g., combination with model checking.^40^ These algorithms will need to be run as an external harness to the Petri net software, which in this case should be the Spike simulator; modifications to the rate constants can be made directly in the corresponding ANDL file, or in the Spike SPC file. Automated optimization over the net structure itself is achievable, but more challenging computationally,^42^ and is best achieved manually.

**CRITICAL:** In scanning, the choice of parameter ranges and scanning intervals is important in order to identify suitable solutions.

10. Store components in a library.

***Note:*** Components should be stored in a library, which could be flat-files in a designated folder structure, or in a database, for an example see Blätke et al.^15^^,^^22^ Some form of version control is preferable, and ANDL and CANDL files are ideal for this treatment; Snoopy files can also be used in order to preserve graphical and layout information, as long as they are saved in the uncompressed XML format. There are many version control systems available, and the choice of which system to use may depend on the local technical setup (troubleshooting 3).

### System-level modeling using color

**Timing: hours to days based on model's complexity**

This part describes how to assemble a system from components by the use of color. System-level modeling starts with retrieving components from the library; in doing so, they become instances. Their use involves a couple of steps outlined as follows.

11. Encode multilevel aspects of the model.
  ***Note:*** Using color sets and logical expressions, we can represent different dimensional space and describe how instances are organized in space. In the following, we use the encoding of 2D space based on Cartesian coordinates as a running illustration, going into depth where necessary. In the following in (a) we describe how to encode this from scratch, and in (b) how definitions stored in a library can be reused.
  a. Encoding from scratch.
    i. Encode space with color.
      ***Note:*** We first define two constants M and N, denoting the length and width of a regular rectangular grid, respectively, and then declare two simple integer color sets, Row and Column, to represent the row and column of the grid. Based on the two simple color sets, we further define a Cartesian product color set Grid2D, where the coordinates (x,y) address a position in the grid. See Figure 9 for an example.
      ***Optional:*** Alternatively, space encoding using polar coordinates or graphs could be employed; see Section 10 of supplemental information for all of these.

**Figure 9:**
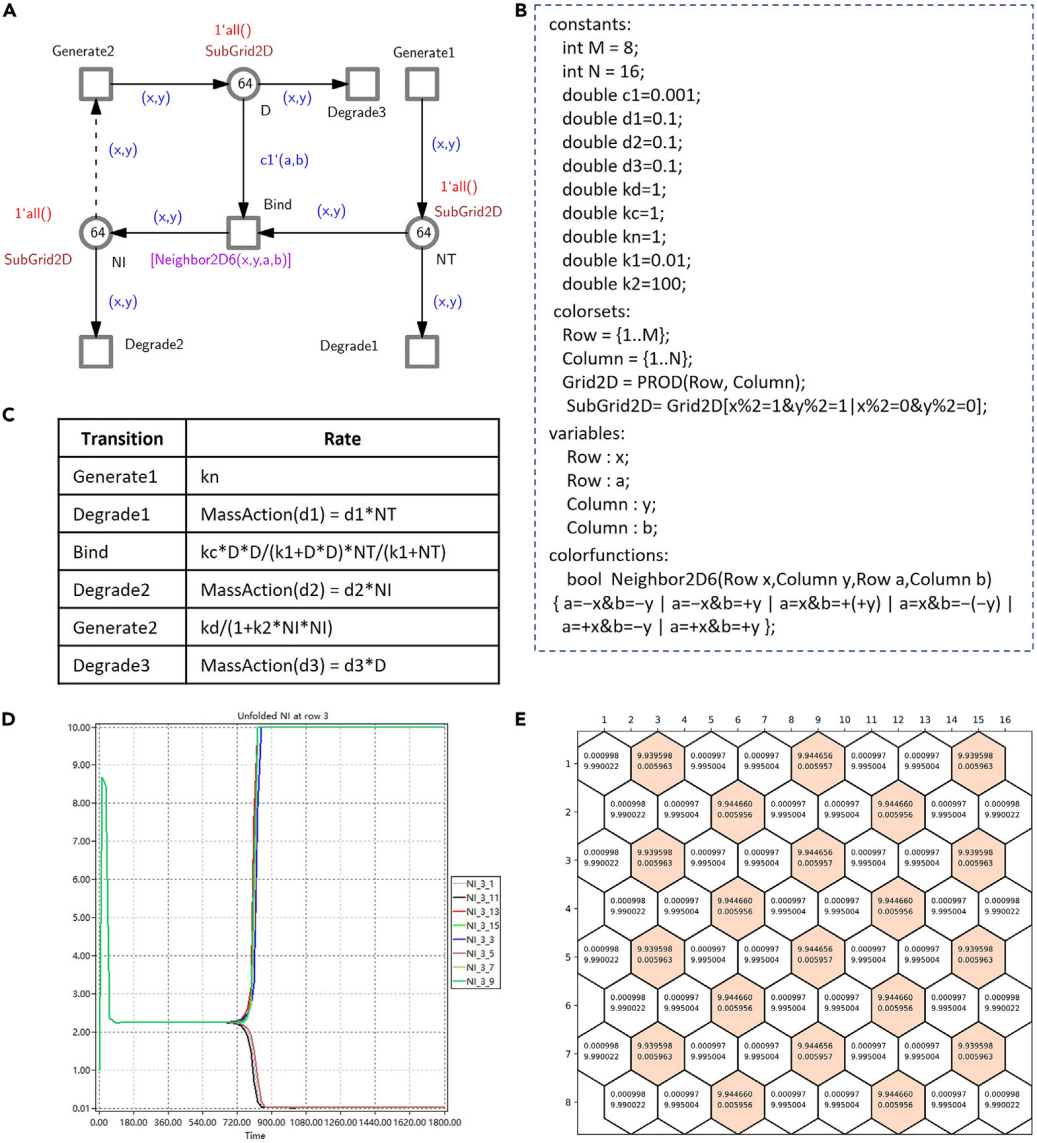
Case study: Delta Notch pattern formation (A) The multicellular Delta Notch ColCPN, which models the lateral inhibition patterning of Delta Notch mutual inactivation,^35^ by coloring the component model given in Figure 8. This model can be used to simulate the pattern formation mediated by the Delta Notch signaling pathway in 2D space, which involves three levels: tissue, cell and Delta Notch pathway. The level of NI in a cell determines the fate of the cell: a low (high) NI level leads to the adoption of the primary (secondary) fate. (B) The declarations of the ColCPN model. Each cell is modeled as a hexagon, and distinguished by a product color in SubGrid2D. When M = 8 and N = 16, we obtain 64 cells. In each cell, we set the initial concentration of each species (D, NT and NI) to 1 with the marking expression 1′all(), which makes in total for each colored place 64. (C) The rate functions and constants used to produce (d), which are adapted from Collier et al.^35^ (D) Simulation traces of Delta and Notch in all cells produced by Snoopy in the setting of (c); shown are the unfolded places of NI for the third row of the hexagonal tissue. (E) A steady-state periodical pattern of the multicellular model, derived from (D). Each hexagon has two numbers, the NI concentration on the top and the D concentration on the bottom. Cells with high NI (secondary fate) are colored, while cells with low NI (primary fate) are given in white.
    ii. Represent instances with colors.
      ***Note:*** If each instance has a unique location on a Cartesian grid, we can denote each instance simply by (x,y). That is, each tuple color in Grid2D represents the location (address) of an instance. If each instance is located in another geometry, e.g., hexagonal, we have to adopt logical expressions to define constraints over Grid2D in order to obtain only those locations which conform to positions in hexagonal space. For example, in the Delta Notch model we can define a subset SubGrid2D by selecting the colors in Grid2D which satisfy the logical expression x%2=1&y%2=1|x%2=0&y%2=0 (to be read as: either the row or column indices are both odd or both even), i.e., selecting all hexagons in Figure 9D. See Section 10 of supplemental information, Figure (c_i_) for more explanations.
      **CRITICAL:** Depending on the specific biological scenario to be represented, an appropriate spatial organization should be employed, e.g., hexagonal or rectangular organization of 2D space, see Section 10 of supplemental information**.**
    iii. **Define neighborhood functions to represent interactions between instances.** Take the 2D space as an example. For any two grid locations with coordinates (x,y) and (a,b), respectively, we can use a logical expression to define a neighborhood function, i.e., whether cell (x,y) is a neighbour of cell (a,b); see Neighbor2D6 in Figure 9, which defines a neighborhood function on a hexagonal geometry with at most six neighbors and the periodical boundary condition. Figure 2 gives two more neighborhood functions which define at most four or eight neighbors respectively.
      **CRITICAL:** Defining neighborhood functions is a very important issue, and care needs to be taken when doing this (troubleshooting 4).
    iv. Represent multilevel with color sets.
      ***Note:*** Colored Petri nets permit the definition of nested color sets, which can represent multiple levels in a biological system. Consider the modeling of a piece of tissue described above. If we use Grid2D to represent cells in a tissue and define another color set, e.g., Compartment2D, in which each element $\left(x_{1},y_{1}\right)$ represents a compartment, we then define a high-level color set, e.g., HCS = product Grid2D × Compartment2D to represent the two levels: cells and compartments. That is, each element ((x,y),(x1,x2)) in HCS locates the position of an instance, where (x,y) denotes the position of a cell in the grid, and (x1,x2) denotes the position of the compartment within that cell. See Section 10 of supplemental information, Figure (c_i_) and (c_ii_) for an illustration and Gao et al.^13^ for an example.
  b. Reuse of color definitions
    i. Store definitions in a CANDL file.
      ***Note:*** Color definitions in a model can be stored by exporting the model to a CANDL file, which is a text file that can be edited by any editor. This permits the careful addition of definitions from other CANDL files to this CANDL library file.
    ii. Load definitions from a CANDL file.
      ***Note:*** Using File → Import, select the CANDL file containing the definitions. The subsequent dialog will permit the import of all or selected constants, color sets, color functions and variables, to an existing model or to a new model.
12. Multilevel model assembly.
  ***Note:*** Assemble a multilevel model by putting an instance on each spatial grid cell and associating appropriate neighborhood expressions to interactions among instances and across different levels. See Section 10 of supplemental information for more details. For the constructed instance models (e.g., the Delta Notch instance model given in Figure 7A), we need to perform the following steps to achieve a multilevel model.
  a. Assign color sets to places.
    i. Double click a place, and then edit its properties.
    ii. In the properties editing dialog, choose an appropriate color set (e.g., SubGrid2D) and possibly define its initial marking in the ‘Markings’ tab.
      **CRITICAL:** In contrast to the marking setting for uncolored Petri nets, where we only need to give a number in the marking domain, for colored Petri nets, in the ‘Markings’ tab we should give a set of colors or a guard to select a group of colors in the ‘Color(s)’ domain, and the corresponding token number for the selected colors in the ‘Marking’ domain. For example, in the Delta Notch model, we set the ‘Color(s)’ domain to ‘all()’, and insert the number 1 in the ‘Marking’ domain for place D (troubleshooting 5).
  b. Define guards for transitions.
    i. Double click a transition, and then edit its properties.
    ii. In the properties editing dialog, fill in a logical expression in the ‘Guards’ tab, e.g., Neighbor2D6(x,y,a,b) for transition Bind.
      ***Note:*** We can also check a guard by clicking the ‘Check guard’ button. Besides, a guard writing assistant is offered by clicking the ‘Guard assistant’ button.
      **CRITICAL:** In the guard assistant dialog, all the information for defining a guard is provided, including available variables and constants, and all the operators and built-in functions. We recommend making full use of this facility to write correct guards (troubleshooting 6).
  c. Define rate functions for transitions.
    i. Double click a transition, and then edit its properties.
    ii. In the properties editing dialog, fill in a logical expression (e.g., true) in the ‘Predicate’ domain and a corresponding rate function (e.g., kc∗D∗D/(k1+D∗D)∗NT/(k1+NT) for transition Bind) in the ‘Function’ domain in the ‘Functions’ tab.
      **CRITICAL:** The logical expression in the ‘Predicate’ domain is used to select a group of transition instances for which a specific rate function is assigned. Multiple logical expressions can be defined, each occupying a row, by using the ‘Add functions’ and ‘Delete functions’ buttons. Make sure that each logical expression covers a group of appropriate transition instances, and that they are mutually exclusive. If a transition instance is not covered by any logical expression, its rate function is set to zero.
  d. Assign expressions to arcs.
    i. Double click an arc, and then edit its properties.
    ii. In the properties editing dialog, fill in an expression (e.g., (x,y)) in the ‘Expression’ domain in the ‘Functions’ tab.
      ***Note:*** We can also check an expression by clicking the ‘Check expression’ button. Besides, an expression writing assistant is offered by clicking the ‘Expression assistant’ button (troubleshooting 7).
  e. Check syntax of the model.
    ***Note:*** Click the ‘Check a net’ item in the ‘Edit’ menu, and a dialog appears, in which click ‘OK’ button and you will see if the model has syntax errors or not.

### System-level analysis involving color

**Timing: hours to days based on model's complexity**

This part describes the steps for thoroughly analyzing a constructed system model. All steps have to consider the set of requirements contained in the Problem Statement & Requirements Specification, in order to validate the system model against them, and to modify either of them accordingly, if required.

13. **System animation** (appropriate for ColPN, ColXPN, ColSPN). Snoopy also supports animation of some classes of colored Petri nets including ColPN, ColXPN, and ColSPN.

**Table 3:** Unfolding methods

Unfolding method	Description
IDD-based unfolding (IDD dssd_util)	Represents the unfolding solution space symbolically as Interval Decision Diagrams (IDD); outperforms often, but not always the following two methods.^55^ Set as default.
Gecode-based unfolding (Geocde dssd_util, or Geocde intern)	Deploys an off-the-shelf CSP (constraint satisfaction problem) solver by means of the library Gecode,^56^ which is usually more efficient than the following method.^57^
Generic unfolding (Generic intern)	Applies templates and basically uses a similar pattern matching mechanism,^57^ as CPN tools.^58^

***Note:*** This can be done basically in the same way as for uncolored Petri nets discussed above, except the following two points. (a) When the animation dialog is open, run the animation by clicking e.g., the ‘Play forward’ button; an unfolding dialog appears. When the unfolding is finished, the animation starts. This is because the animation of a colored Petri net is done on an uncolored Petri net by automatic unfolding. In the unfolding dialog (see Figure 7B), choose an unfolding method (see Table 3) and click the ‘Start’ button; the unfolding will be performed with a progressing bar and the unfolding run time. Although unfolding is a combinatorial problem, which suffers from a combinatorial explosion, the use in the platform of a constraint satisfaction approach for unfolding greatly alleviates this issue. (b) In the manual mode, by clicking a transition, a binding selection dialog will appear, in which we can choose a binding for firing. See Section 16 of supplemental information for more information.

14. **System structural analysis** (appropriate for all Petri nets that do not involve special arcs (inhibitor, equal, reset and self-modifying arcs, see Section 3 of supplemental information) which bring the Turing power).

***Note:*** Basically follow the workflow as outlined in Step 6| Component structural analysis. However, the export to the file format ANDL readable by Charlie now involves the unfolding of the colored model. Correspondingly, all analysis results produced by Charlie refer to the unfolded model. See Sections 18-22 of supplemental information for how to perform structural analysis.

**CRITICAL:** The size of the unfolded model may exceed what Charlie can handle in reasonable time. Thus, it is advisable to configure the system model to its smallest possible size to exhibit the required behavior before exporting to ANDL (which involves unfolding). The interpretation of Charlie’s analysis results may require a good understanding of the naming conventions used for the unfolded nodes, see Section 27 of supplemental information (troubleshooting 8).

15. **System simulation** (appropriate for ColSPN, ColCPN, ColHPN). Explore the multilevel model using different simulation algorithms including stochastic, deterministic and hybrid simulation algorithms. See Section 17 of supplemental information for how to run simulation. The simulation of colored Petri nets is slightly different from that of uncolored Petri nets. In the following, we only describe different points.
  a. When clicking the ‘Start Simulation-Mode’ item in the ‘View’ menu, an unfolding dialog appears, rather directly going to the simulation dialog.
    ***Note:*** An appropriate unfolding engine should be chosen, see Table 3. This is because the simulation of a colored Petri net is also performed on an uncolored Petri net by automatic unfolding.
  b. When the unfolding is done, the simulation dialog (see Figures 7C and 7E) will be opened.
    ***Note:*** Almost all operations on the simulation for the colored Petri nets are the same as uncolored Petri nets. The only difference is that we can observe both colored and uncolored places and transitions. In the view window, click the ‘Edit Node List’ button, the node selection dialog appears, where one can select uncolored or colored places/transitions (supported by regular expressions) to be displayed in the view window. See Section 27 of supplemental information for data about size of the unfolded models for some case studies.
    **CRITICAL:** A ColPN usually involves a large number of unfolded places/transitions. The platform offers several ways to support the selection of appropriate nodes in the node selection dialog (see Figure 7G), including: (a) manually choosing several nodes, (b) using the name of a colored node to obtain all of its unfolded nodes with the ‘Advanced’ dropdown list, and (c) using a regular expression in the ‘RegEx’ textbox to select nodes.
  c. Export simulation results (see Figure 7F for an example) to a CSV file, if they are to be analyzed in the next step (troubleshooting 9).
16. System dynamic analysis.
  ***Note:*** Analyze the model at system level using different techniques such as the exploration of 2D/3D plots and movies, simulative model checking driven by temporal-logic formulae, and statistical analysis, e.g., clustering.
  a. Simulative model checking. For a general discussion on model checking and its use in the context of our platform, see Section 23 of supplemental information.
    ***Note:*** Colored Petri net models are usually large, so analytical model checking often does not apply. To use simulative model checking at the system level, follow the model checking steps described above in Step 8/A/c&d. Write a query file for colored/uncolored places or transitions and perform model checking. E.g., in our Delta Notch model, we use the property P>=1[ F(G([NI_x_y] > 9 ˆ [D_x_y] < 1) ) ] to check if a cell adopts the primary fate (yes) or the secondary fate (no). In the property, NI_x_y (or D_x_y) refers to all the uncolored places of NI (or D) obtained by unfolding. For more examples about simulative model checking at the system level, refer to Gao et al.^13^ and Liu et al.^23^
  b. Time series data analytics.
    ***Note:*** The simulation output comprises time series traces of the values of place markings or transition activity, which can be saved in the form of CSV (or equivalent) files. As at the component level (see Step 8/b), these data can then be analyzed using standard time-series data analytic techniques using software such as MATLAB or R. The actual methods used depend on the problem being investigated; for possible options see Section 24 of supplemental information. In particular, for a colored system model, the potentially large amount of unfolded data, including location information, introduces extra complexity to the analysis.
    ***Note:*** In particular, visualization techniques are extremely useful to gain an understating of the behavior of the multidimensional model:
    ***Optional:*** 2D plot drawing. Take the python routine as an example, which are used for drawing the pattern of the Delta Notch model (see Figure 9D), to illustrate this analysis.
    i. Select simulation results of interest (e.g., all the unfolded places of D and NI of the Delta Notch model given in Figure 9) and export them to a CSV file with a separator.
    ii. Edit the 2D plot routine (e.g., the python routine 2DHexagonPlot.py given in the key resources table) by modifying the data file name and separator.
    iii. Use python to open the routine 2DHexagonPlot.py and then the 2D pattern is created and shown (e.g., see Figure 9D).
      ***Optional:*** 2D movie generation for reaction-diffusion systems. With the Python 2D movie generation routine (see the 2D plot routine in the key resources table), we can generate a 2D movie for the simulation traces of a 2D diffusion model (see key resources table for a 2D diffusion model), which takes the following steps.
    iv. Select simulation traces of interest (e.g., all the unfolded places of p of the 2D diffusion model given in Figure 2) and export them to a CSV file (e.g., 2D_diffusion_traces.csv) with a separator, e.g., comma.
    v. Edit the 2D movie generation routine (e.g., the python routine 2DMovie.py given in Section 25 of supplemental information) by modifying the data file name, separator, and the row and column of the 2D grid.
    vi. Use Python to open the routine 2DMovie.py and then the 2D movie is created and shown. It should look like the one provided in the key resources table, Movies of 2D diffusion (troubleshooting 10).
17. System optimization.

***Note:*** Adjust kinetic parameters and neighborhood expressions according to the whole system analysis results, against the corresponding reference traces or patterns over space and time.

***Note:*** E.g., in the boundary formation model of the *Drosophila* large intestine,^23^ we set most kinetic parameters to fixed values and then tune three key parameters, a, b and f by repeating simulations until the model produces the wild-type phenotype. This process can be very time consuming. Automated approaches to target driven optimization are in general preferable to parameter exploration via scanning.

### Model use

**Timing: days to years based on model's complexity**

This part very briefly describes possible, not necessarily alternative scenarios to use a model.

18. Formalizing and organizing knowledge.

***Note:*** Models can be used to formalize and organize existing knowledge about a biological system. Indeed this is often the first step in modeling a biological system; the modeler will ‘interview’ biologists and try to get them to describe the system of interest using some or all of the following: natural language, pictures and diagrams of varying degrees of formality or a possibly incomplete or flawed collection of biochemical equations. The skill of the modeler is then to represent this knowledge in a form which is meaningful to the biologists, and to hopefully get them to agree the model or suggest changes. Petri nets provide an excellent way in which to communicate with biologists in this respect because they represent the topology of a model in a very intuitive and graphical manner. Biologists very often readily accept this representation, and will suggest corrections to a model because the process of constructing the model jointly with the modeler has clarified their own understanding of the biological system. See for example Section 4 of supplemental information, which illustrates a modular and stepwise construction of a Petri net model of a repressilator.

19. Prediction and hypothesis validation.
  ***Note:*** The biological systems over which predictions can be performed range from the intra/inter-cellular level to epidemic/pandemic at the level of societies^59^; see Figure 1A (systems biology). In Section 26 of supplemental information, we summarize some published case studies undertaken with our platform, ranging from gene transcription & regulatory networks via signal transduction networks and metabolic networks to population level, which all offer rich information on how to use models for prediction.
  a. Analyze a new hypothesis and accordingly configure the model by modifying the marking, rate parameters, or the net structure.
  b. Run the simulation to obtain the simulation result.
  c. To validate a hypothesis in the form of predictions, perform *in vivo* experiments, or observe the changes in the modeled system as it progresses over time or is exposed to changing environments.
    ***Note:*** For example, in Liu et al.^23^ we want to validate the hypothesis: over-expression of Delta results in different patterns of embryos in the *Drosophila* large intestine. We started from the normal expression level of Delta, which produced the wild-type phenotype, and then slowly increased the over-expression of Delta by a small step, successively producing ectopic phenotype, ectopic & drop-outs phenotype and salt & pepper phenotype (see Liu et al.,^23^; Figure 8). We then performed *in vivo* experiments by growing embryos at different temperatures (the increase of temperature causes the over-expression of Delta signal), which produced similar patterns (see Liu et al.,^23^; Figure 11) as simulation did.
20. Engineering.

***Note:*** Synthetic biology - engineering biological systems; see Figure 1B. In Section 26 of supplemental information, some case studies such as biosensors and DNA walker circuit design have been summarized.

***Note:*** Assuming that a model has been constructed which has a behavior that conforms to the requirements specification: (a) Use the model as design to guide engineering of the corresponding biological system. (b) Check that the behavior of the engineered biological system conforms to the behavior of the model.

## Expected outcomes

The protocol exploits our ColPN technique in order to construct and analyze biological models ranging from unilevel to multilevel. As part of the workflow, the following are developed within a sound BioModel Engineering framework ensuring reproducibility of results: a library of components, a library of spatial constructs, a set of system models, synthetic (simulated) data relating to the system models, and a set of analyses of the simulated data, comprising data visualization.

With this technique, we can construct multilevel models, for example the Delta Notch model given in Figure 9 to study pattern formation issues widely existing in biological systems. The Delta Notch model can be reproduced with the model file given in the key resources table, which can produce patterns like those given in Figure 9D. Such a model can be easily reconfigured to simulate different scenarios like mutations by changing the following items: (i) marking set, (ii) rate constants, and (iii) neighborhood functions and boundary conditions.

Moreover, we have applied this ColPN technique to study more multilevel biological phenomena; see Section 26 of supplemental information for a brief summary and references.

## Limitations

Aspects of BioModel Engineering, which are currently not supported or only partially supported, include: database permitting the storage and reuse of model components, version control of models, optimization, and built in advanced data analytics.

## Troubleshooting

### Problem 1

Border node and coarse node are from the same type (related to Step 4).

### Potential solution

Border node and coarse node should be of different types. Make sure that all the border nodes are of the same type (place or transition) and then coarse these nodes into a macro node of the different type (transition or place).

### Problem 2

Deterministic simulation does not make progress (related to Step 7 &15).

### Potential solution

• Check if simulation did start by looking in the simulation output table for non-zero entries.
• Check log window for possible reasons.
• Reduce the size of the colored model.
• Use stiff solver or switch to stochastic model using an approximative SSA (tau leaping, delta leaping) or hybrid model and treat slow reactions as stochastic and fast reactions as continuous.

### Problem 3

Stochastic simulation does not make progress (related to Step 7 &15).

### Potential solution

This is possibly because stochastic model can contain dead states, or extreme increase in reaction rates. Solve this issue in the following steps:

• Check log window for possible reasons.
• Reduce the size of the colored model.
• Use an approximative SSA (tau leaping, delta leaping).

### Problem 4

Deterministic simulation trace contains negative values (related to Step 7 &15).

### Potential solution

This is possibly because the step size used by the solver is not appropriate. Solve this issue in the following steps:

• Use another solver.
• Adjust solver parameters (initial step size, relative and absolute tolerances).

### Problem 5

Expected simulation behavior is not apparent (related to Step 7 &15).

### Potential solution

This is possibly because simulation traces are too short, or do not contain sufficient time points. Solve this issue in the following steps:

• Increase / decrease simulation time;
• increase the number of output steps.

### Problem 6

Unexpected results from simulative model checking (related to Step 8 &16).

### Potential solution

This is possibly because model checking results may depend on the output step size and the solver used to generate the traces. Solve this issue in the following steps:

• Increase output step size;
• Use different solvers.

### Problem 7

Losing control of the model versions (related to Step 10).

### Potential solution

Use version control system, choices from e.g., https://en.wikipedia.org/wiki/Comparison_of_version-control_software.

### Problem 8

The neighborhood function does not correctly describe the neighbors (related to Step 11).

### Potential solution

Double check the function to validate that the defined neighborhood function correctly describes the neighbors. If it is used in the guard of a transition, then by clicking the “Show bindings” button, all legal bindings can be seen.

### Problem 9

Color mismatches the color set (related to Step 12).

### Potential solution

Check the arc expression to ensure that its type is of the color set of the corresponding place.

### Problem 10

Expression syntax error (related to Step 12).

### Potential solution

Check the expression to make it comply with the syntax given in the manual.^60^ Especially pay attention to use the backquote (`) rather than single quote (‘).

### Problem 11

Guard is not a Boolean expression (related to Step 12).

### Potential solution

Check the guard to assure that it is evaluated to a Boolean value.

### Problem 12

Error in the operands of equal/unequal operator (related to Step 12).

### Potential solution

Check the Boolean expression to make both operands be of the same type.

### Problem 13

Generating the unfolded model by export does not appear to generate a file (related to Step 14).

### Potential solution

• Check log window if the size of the unfolded model is given then the model has been generated and saved. Thus check that the destination folder was correctly given.
• If there is an error reported in the log window, then there is an error in the colored model which needs to be resolved before export.

### Problem 14

Simulation appears not to start (related to Step 15).

### Potential solution

• Check colored model.
• Reduce model size. See Section 27 of supplemental information for some examples of size increase on unfolding.

### Problem 15

Colored model does not behave as expected (related to Step 15&16).

### Potential solution

• Assuming that the model is scalable, set the constants defining the color sets as small as possible to reduce complexity. Export the unfolded model, and inspect it by (i) looking at the net layout, (ii) counting nodes & arcs, (iii) analyzing the structure using Charlie.
• Inspect simulation traces, both colored and uncolored, for unexpected behavior.

## Resource availability

### Lead contact

Further information and requests for resources and reagents should be directed to and will be fulfilled by the lead contact, Fei Liu (feiliu@scut.edu.cn).

### Materials availability

This protocol did not generate new materials.

## Data Availability

• Our models, videos, and routines, as listed in the key resources table, are all available on https://github.com/PetriNuts/MultilevelModelling. • All tools of our platform are freely available for non-commercial use, which can be downloaded from https://www-dssz.informatik.tu-cottbus.de/DSSZ/Software/Software. • Any additional information required to reanalyze the data reported in this paper is available from the lead contact upon request.

## Supplemental information

Document S1. Petri net compendium
