# Supplementary material for: Protocol for biomodel engineering of unilevel to multilevel biological models using colored Petri nets
Source: STAR Protoc. 2023 Dec 8;4(4):102651. doi: 10.1016/j.xpro.2023.102651 (PMC10751555; doi:10.1016/j.xpro.2023.102651)
Supplement: Document S1. Petri net compendium [file mmc1.pdf]

# Contents


1. Basic Petri nets - terminology ........ 3
2. Basic Petri nets - additional features ........ 4
3. Advanced Petri nets - modelling features ........ 5
4. Example - Repressilator ........ 7
5. Quantitative net classes ........ 8
6. Fuzzy Petri nets ........ 10
7. Coloured Petri nets - Glossary of basic notions ........ 11
8. Coloured Petri nets – basic syntax ........ 14
9. Coloured Petri nets - folding and unfolding ........ 15
10. Encoding space - principles and examples ........ 17
11. Multilevel, Multiscale and Multidimensional ........ 19
12. Example – Delta Notch signalling pathway ........ 21
13. Snoopy - Petri nets elements ........ 23
14. Snoopy - basic features ........ 25
15. Snoopy - other useful features ........ 26
16. Snoopy - animation configuration ........ 27
17. Snoopy - simulation configuration ........ 28
18. Charlie's Graphical User Interface ........ 31
19. Behavioural properties ........ 32
20. Elementary graph properties ........ 33
21. Basic notions of structural analysis ........ 34
22. Snoopy - Charlie interplay ........ 36
23. Model checking ........ 37
24. Data analytics ........ 39
25. Tools of the Petri net platform to support the workflow ........ 40
26. Selected case studies undertaken with the platform ........ 42
27. Unfolding data ........ 44
28. Abbreviations ........ 45

References ........ 47

## 1. Basic Petri nets - terminology

Basic Petri nets are discrete as well as being time-free and thus qualitative in nature. They are associated with the following terminology. Note that we use the terms 'edge' and 'arc' interchangeably.

**Node**. There are two types of nodes: places and transitions. Statements, which equally apply to places and transitions, often simply speak of nodes.

**Place**. Places, graphically represented by circles, usually stand for passive system components like local conditions or resources. In the biological area, places may represent species or any kind of chemical compounds, e.g., genes, gene states, proteins, protein complexes, metabolites; see [BHM15], Figure 7.5 for more examples.

**Transition**. Transitions, graphically represented by rectangles, usually stand for active system components like events. In systems biology, transitions may represent any kind of chemical reactions, e.g. association, disassociation, translation, transcription, or transport steps; see [BHM15], Figure 7.6 for details.

**Arc**. An arc, graphically represented by an arrow, leads from a place to a transition, or from a transition to a place. The (non-negative integer) arc weight indicates its multiplicity, reflecting, e.g., stoichiometry of a chemical reaction. The weight 1 is usually not shown.

**Pre/post-nodes**. Regarding a specific node, we may wish to refer to all nodes

- from which an arc is coming to that node– the set of pre-nodes (immediate predecessors), and
- to which an arc is going from that node –the set of post-nodes (immediate successors).

Distinguishing between places (species) and transitions (reactions), we obtain four special sets:

- pre-places of a transition –the reaction's precursors,
- post-places of a transition –the reaction's products,
- pre-transitions of a place–all reactions producing this species,
- post-transitions of a place – all reactions consuming this species.

**Token**. A place may contain an arbitrary (non-negative) number of tokens, graphically represented by black dots or a natural number.

**Marking**. An allocation of tokens to all the places of a Petri net is called a marking; it gives the current system state of the model. The initial marking specifies the initial conditions, where the observation of the system behaviour starts.

**Enabled**. A transition is called enabled if each of its pre-places contains at least the number of tokens specified by the weight of the corresponding arc.

**Firing**. An enabled transition may fire (occur); the firing of a transition transfers tokens from its pre-places to its post-places according to the weights of the adjacent arcs.

*Example.* Petri net representation for a reaction r with the stoichiometric equation $2H_2+O_2 \rightarrow 2H_2O$, and three states connected each by a single firing (adapted from [BHM15]).

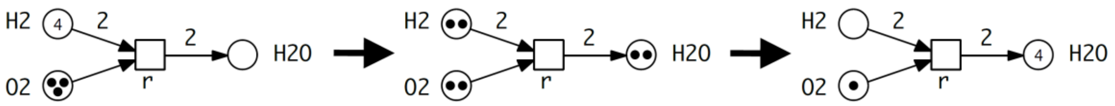


Figure S1. Petri net example.

Precursors become pre-places and the single product of the reaction becomes the post-place of the transition *r*. Vice versa, the transition r is a post-transition of the two places $H_2$ and $O_2$, and a pre-transition of $H_2O$. The stoichiometries appear as arc weights. The initial marking (state) is arbitrarily set to four molecules of $H_2$ and three molecules of $O_2$. In this state, the transition is enabled. Firing the transition consumes tokens on the pre-places, two tokens on $H_2$ and one token on $O_2$, while two tokens are produced on the post-place $H_2O$. The system reaches a state where each place carries two tokens. The transition is still enabled, and a second firing will lead to a state, where no tokens are left on $H_2$, thus the transition is not enabled anymore.

## 2. Basic Petri nets - additional features

We enrich basic Petri nets by the following features to enhance modelling convenience, without extending the expressiveness.

**Constants**. A constant may be varied to configure a model, but always holds a fixed value during a given model evaluation. Constants may be used to specify arc weights or the initial marking. As they permit model configuration, they help avoiding numerous model versions. There are only (non-negative) integer constants for basic Petri nets.

**Functions.** Functions allow the definition of templates usable everywhere where constants are allowed, making models even more flexible and better maintainable than already possible by help of constants.

**Logical nodes**. Logical nodes with the same name are identical, i.e., graphical copies of a single node. They are graphically represented by the colour grey or cross-hatched (if coloured otherwise, see Section 4 for an example). Logical nodes include logical places and logical transitions. Logical nodes are often used as connectors bringing together identical nodes that are repeated in the network structure to reduce crossing of arcs adjacent to nodes that are linked to numerous other nodes.

**Macro nodes**. Macro nodes are used to hierarchically structure a network, which facilitates the design and systematic construction of larger Petri nets. Each macro node creates a new layer of a network. Macro nodes can be arbitrarily nested. Macro nodes include macro places, graphically represented by two nested circles, and macro transitions, graphically represented by two nested rectangles. The boundary nodes of a macro place are transitions, and the boundary nodes of a macro transition are places.

*Example*: Five mathematically identical representations of the same basic enzymatic reaction: Left column, process-orientated – in three versions: **(a)** full (standard) description; **(b)** reversible reaction represented by a macro transition resulting in two hierarchical Petri net levels; **(c)** entire enzymatic reaction represented by one macro transition resulting in three hierarchical Petri net levels. Middle column: **(d)** reaction-oriented – 3 subnets by use of logical places. Right column: **(e)** molecule-oriented – 4 subnets by use of logical transitions (adapted from [BHM15]).

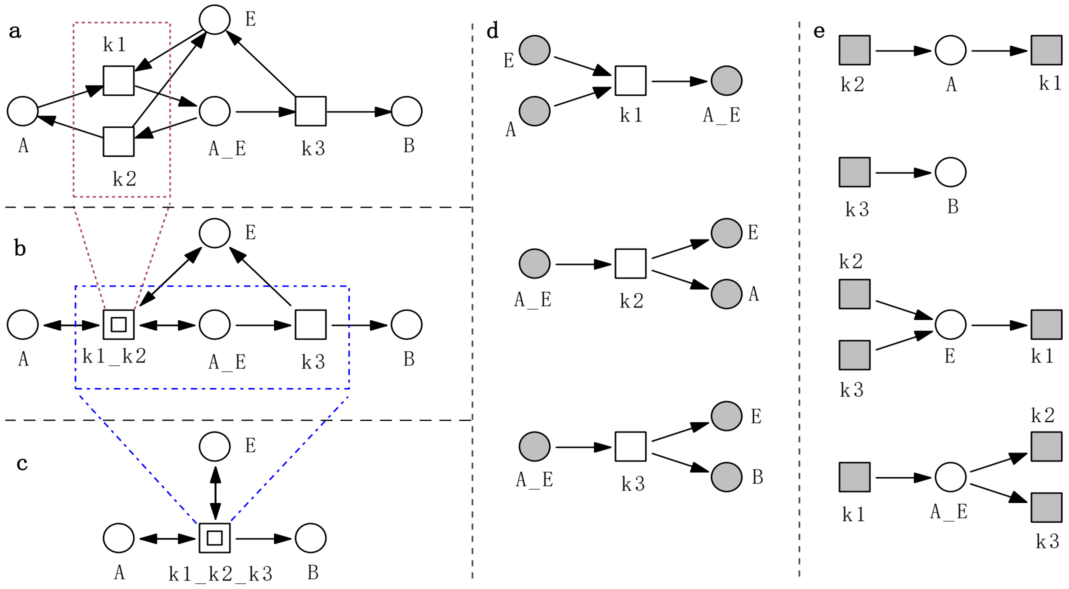


Figure S2. Five mathematically identical representations of the same basic enzymatic reaction.

## 3. Advanced Petri nets - modelling features

The following features extend the expressiveness of basic Petri nets. These are all advanced features developed within the general Petri net community, and not solely for modelling biological systems.

All following special arc types, except self-modifying arcs, go always from a place to a transition; for the graphical representations see the examples below. The first three arc types establish special preconditions for the enabledness of the adjacent transition, while the other three arc types do not have an impact on the enabledness. Upon firing of the transition, the marking of the adjacent place does not change, except for reset and self-modifying arcs.

**Read arc.** A read arc (also known as test arc) allows to model that some resource is read, but not consumed upon firing of the adjacent transition.

**Inhibitor arc.** The inhibitor arc [AF73] reverses the logic of the enabling condition established by a place, i.e. it imposes the precondition that a transition may only fire if the adjacent place contains less tokens than the weight of the arc indicates.

**Equal arc.** The equal arc imposes the precondition that a transition may only fire if the number of tokens of the adjacent place is exactly equal to the arc weight.

**Reset arc.** The reset arc [DFS98] does not impose a special firing condition, but empties the adjacent place upon firing of the transition.

**Self-modifying arc.** A self-modifying arc (also known as state-dependent arc) [Val78] carries as arc weight an expression, involving the adjacent place, which is either pre- or post-place of the connected transition. During model execution, this expression is evaluated and the result determines the current arc weight. Thus, the arc multiplicity changes according to the current marking of the adjacent place. The HPN in Section 11 exploits this feature to represent cell division.

**Modifier arc.** Modifier arcs, inspired by SBML [HFS+03], permit to include any places in a transition's rate function, while simultaneously obeying the syntactic rules of quantitative Petri nets (see Section 5). Modifier arcs do not influence the enabledness, but depending on the mathematical nature of the rate function, the firing rate may become zero if the adjacent place is empty.

*Examples.* Five Petri nets illustrating special arcs; M, W are constants; first row: before firing, second row: after firing for appropriate M, W.

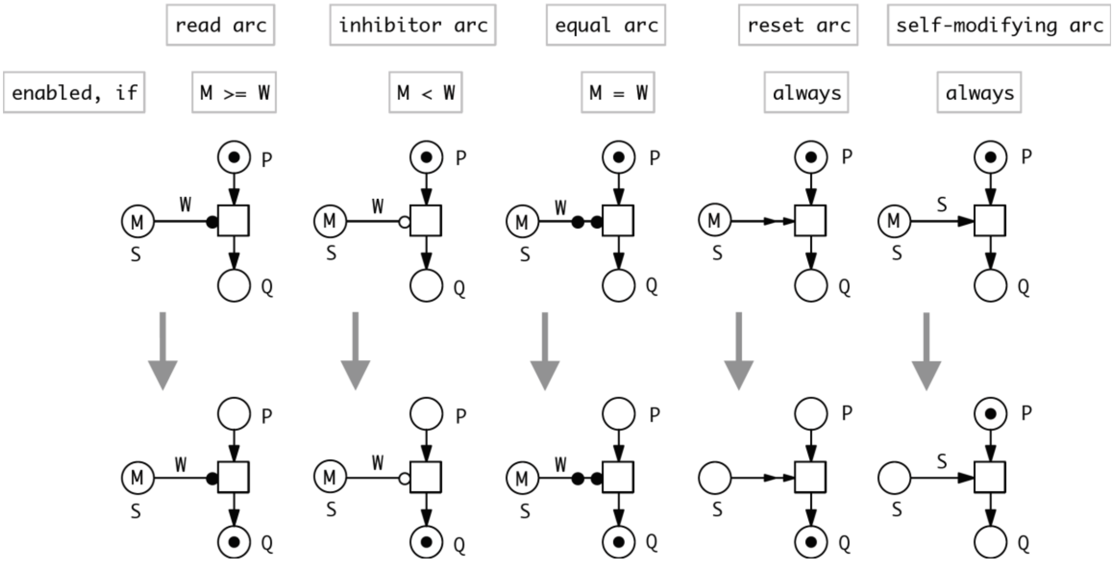


Figure S3. Five Petri nets illustrating special arcs.

*Remarks:*

- Read arcs do not bring the Turing power, but permit distinguishing between exclusive and concurrent access to shared resources, which is specifically useful under the partial-order semantics.
- Inhibitor, equal, reset and self-modifying arcs bring the Turing power. Thus, basic behavioural properties like liveness are not generally decidable anymore [Pet81].
- Modifier arcs only exist in quantitative Petri nets, and are graphically represented by dashed lines.

## 4. Example - Repressilator

The **repressilator** is a gene regulatory network comprising at least three genes, each expressing a protein that represses the next gene in the loop. Repressilators do occur in nature as a basic mechanism in plants and animals e.g. [WOQ+17], but have also been built synthetically in-vitro e.g. [SE00], [OCJ+15]. We use this example to illustrate a modular and stepwise construction of a Petri net model using Snoopy [LH14].

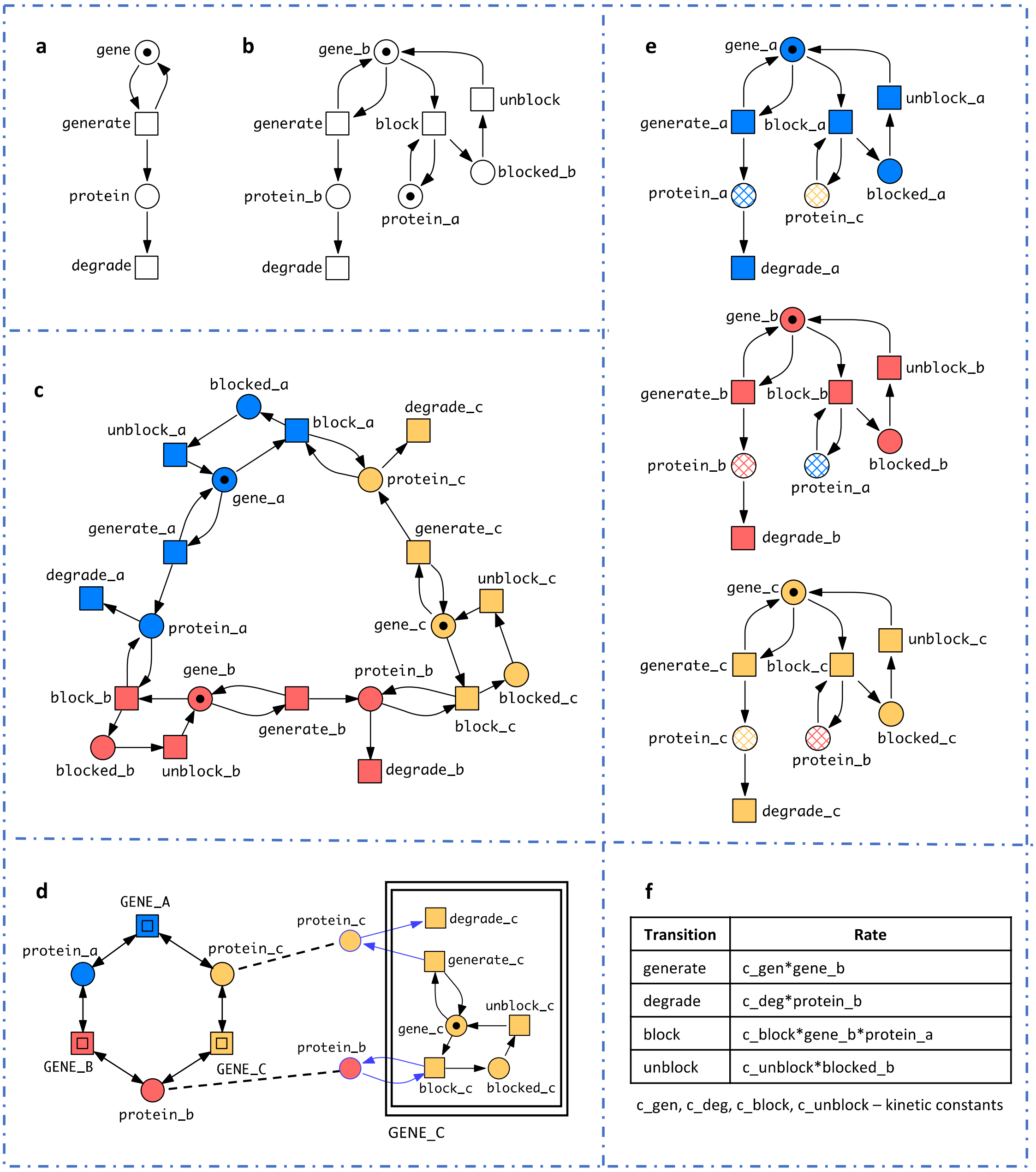


Figure S4. The repressilator example [HG13, LH14]. Some figures are adapted from F. Liu, M. Heiner, Petri Nets for Modeling and Analyzing Biochemical Reaction Networks, in Approaches in Integrative Bioinformatis, 245-272, 2014, Springer Nature.

(a) Petri net model of a gene. The presence of the gene allows the generation of proteins without consuming the gene, while generated proteins can degrade.

(b) Petri net model of a gene gate [BCP08] extends (a) by allowing the gene to be blocked by the protein produced by another gene.
(c) Petri net model of a gene regulatory network composed of three gene gates according to (b). Genes repress each other in a circular manner.
(d) Equivalent Petri net model of the gene regulatory network in (c) or (d) exploiting macro transitions to obtain a hierarchical representation.
(e) Equivalent Petri net model of the gene regulatory network in (c) or (d) using logical places. See also (a) in Section 7, using logical transitions.
(f) The rate functions for the transitions in (b), each following mass action kinetics.

## 5. Quantitative net classes

All the following net classes are extensions of qualitative Petri nets.

**Stochastic Petri nets (SPNs)** extend qualitative Petri nets by associating a delay firing rate with a transition, which is a random variable following exponential probability distribution [ABC+98]. The transition firing rate is often state-dependent and specified by a rate function (see below for details) [HGD08]. The semantics of an SPN is equivalent to a continuous time Markov chain (CTMC), which is isomorphic with the reachability graph of the underlying qualitative Petri net, but the arcs between states are labelled with transition (firing) rates. SPNs as supported by our platform have been further extended by the following special transitions [HLG+09].

- **Immediate transition.** An immediate transition fires with waiting time zero, which always has higher priority compared with any other transition.
- **Deterministic transition.** A deterministic transition fires after a deterministic time delay.
- **Scheduled transition.** A scheduled transition fires at predefined time points.

**Continuous Petri nets (CPNs).** In a CPN [DA10], the discrete token values of places are replaced by continuous values, which describe the overall behavior of species represented by places via concentrations. A deterministic firing rate is associated with each transition, determining the strength of the continuous flow from a transition's pre-places to its post-places. The transition firing rate is often state-dependent and specified by a rate function (see below for details) [HGD08]. The semantics of a CPN is given by a set of ordinary differential equations (ODEs), which turns CPN into a convenient means to graphically represent ODEs in a structured way [BGH+08].

**Hybrid Petri nets (HPNs)** combine both stochastic and continuous elements in one and the same model, as SPN and CPN may perfectly complement each other. Then, fluctuation and discreteness can be conveniently captured using stochastic simulation, while at the same time computationally expensive subnets are simulated deterministically using ODE solvers [HH12].

**Fuzzy Petri nets (FPNs)** support fuzzy numbers as kinetic parameters (see Section 6). Thus, they extend the quantitative net classes SPN, CPN and HPN, where all kinetic parameters have to be crisp values, yielding FSPN, FCPN, and FHPN [AHL19].

**Coloured Quantitative Petri nets.** Adding the concept of colour to SPN, CPN, and HPN yields the corresponding high-level net classes ColSPN, ColCPN, ColHPN [HHL+12]; likewise, combining ColPN with FSPN, FCPN, and FHPN yields ColFSPN, ColFCPN, and ColFHPN [AHL21].

There are two closely related key concepts applying equally to all quantitative net classes.

**Rate functions** are technically mathematical functions, which are generally state-dependent by the involvement of Petri net places. To keep a close relation between Petri net structure and behaviour, the syntactic constraint has been introduced to only use a transition's pre-places in its rate function, which can always be achieved by the use of modifier arcs. Rate functions often follow specific kinetic patterns, such as mass-action kinetics, which are supported by predefined functions, e.g. MassAction($k$), where $k$ is a rate constant. In the case of coloured Petri nets, rate functions can also be colour-dependent.

**Simulation traces.** Typically, a first step in analysing the behaviour of these models is to simulate them and to record time series for further analysis, using, e.g., model checking (see Section 23) or data analytics (see Section 24). There are two types of simulation traces: over (a) places, and (b) transitions.

(a) Simulation traces over places show the number of tokens at the selected places change over time.

(b) Simulation traces over transitions show the firing rate during the last observed time interval.

*Example*: Using the repressilator case study from Section 4 and reading it as a stochastic Petri net.
(a) Time series traces for places (protein concentrations).
(b) Time series traces for transition activity.

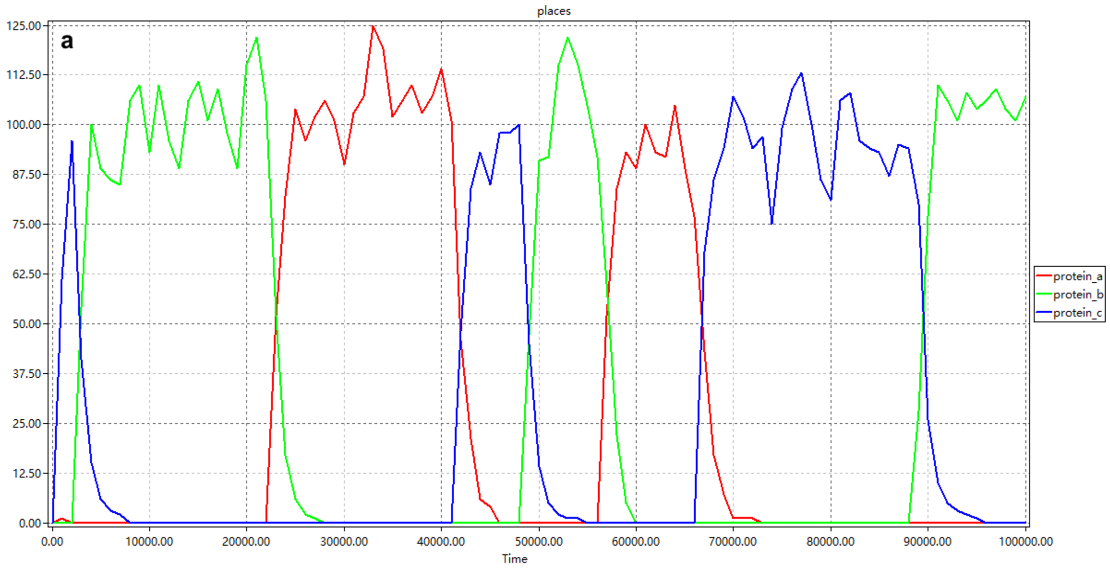

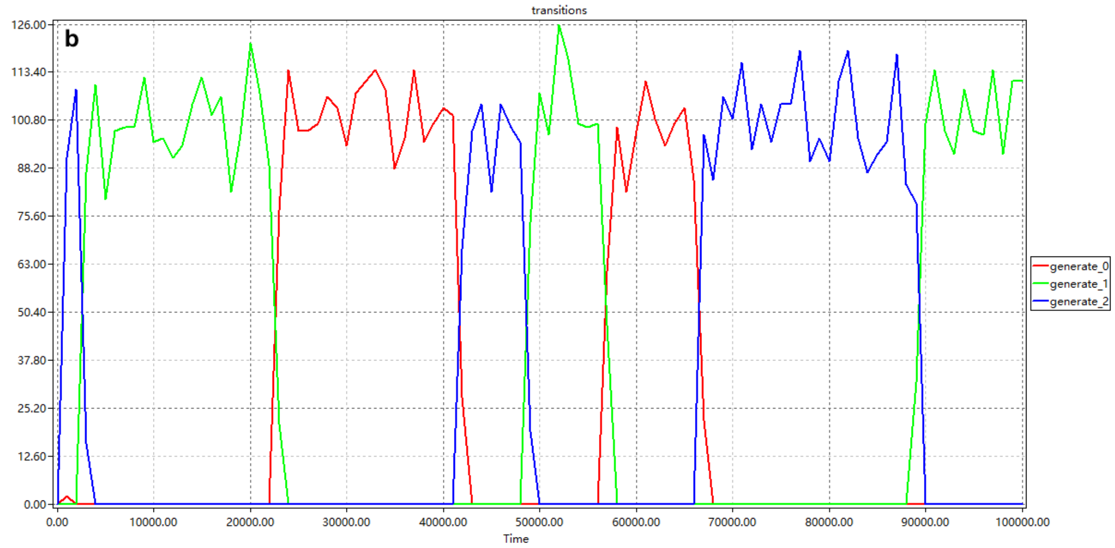


Figure S5. Simulation traces of the repressilator model.

## 6. Fuzzy Petri nets

Fuzzy Petri nets (FPNs) [LHG18] extend quantitative Petri nets by allowing kinetic parameters to be either fuzzy numbers or crisp numbers, which generates three new uncoloured net classes [AHL19]:

- fuzzy stochastic Petri nets (FSPNs),
- fuzzy continuous Petri nets (FCPNs), and
- fuzzy hybrid Petri nets (FHPNs),

and three new coloured net classes [AHL21]:

- coloured fuzzy stochastic Petri nets (ColFSPN),
- coloured fuzzy continuous Petri nets (ColFCPN), and
- coloured fuzzy hybrid Petri nets (ColFHPN).

FPNs are appropriate to model biological systems when kinetic parameters cannot be estimated or measured precisely.

Take the coloured repressilator model (given in Section 4) as an example. If we consider a kinetic parameter, e.g. k1, as a triangular fuzzy number (see Figure (a)), e.g. (0.05, 0.1, 0.12), we obtain a fuzzy model. Compare the rate functions given in the following table.

Table S1. The rate functions of the coloured repressilator model.

| Transition | Rate function | Kinetic parameter |
|---|---|---|
| generate | MassAction(k1) | k1 = (0.08, 0.1, 0.12) |
| degrade | MassAction(k2) | k2 = 0.001 |
| blocked | MassAction(k3) | k3 = 1 |
| unblocked | MassAction(k4) | k4 = 0.0001 |

Due to the uncertainties of kinetic parameters, the simulation of an FPN produces an uncertain band of each output and the membership functions over time. To achieve this, we first decompose each fuzzy number into a set of α-cuts, and then draw samples (crisp values) for all fuzzy numbers at each α-level. For each sample combination, we run (stochastic, deterministic, or hybrid) simulation and obtain traces of the outputs of interest. There are two sampling strategies: basic and reduced; see Figure (b) for an illustration of the reduced sampling strategy.

For example, Figure (c) gives a fuzzy band (between two curves of the same colour) of each of the unfolded places, protein_a, protein_b and protein_c, while Figure (d) gives the membership function of protein_a at the time point 75. See also the video in Key sources table: Fuzzy coloured Petri nets (Repressilator example).

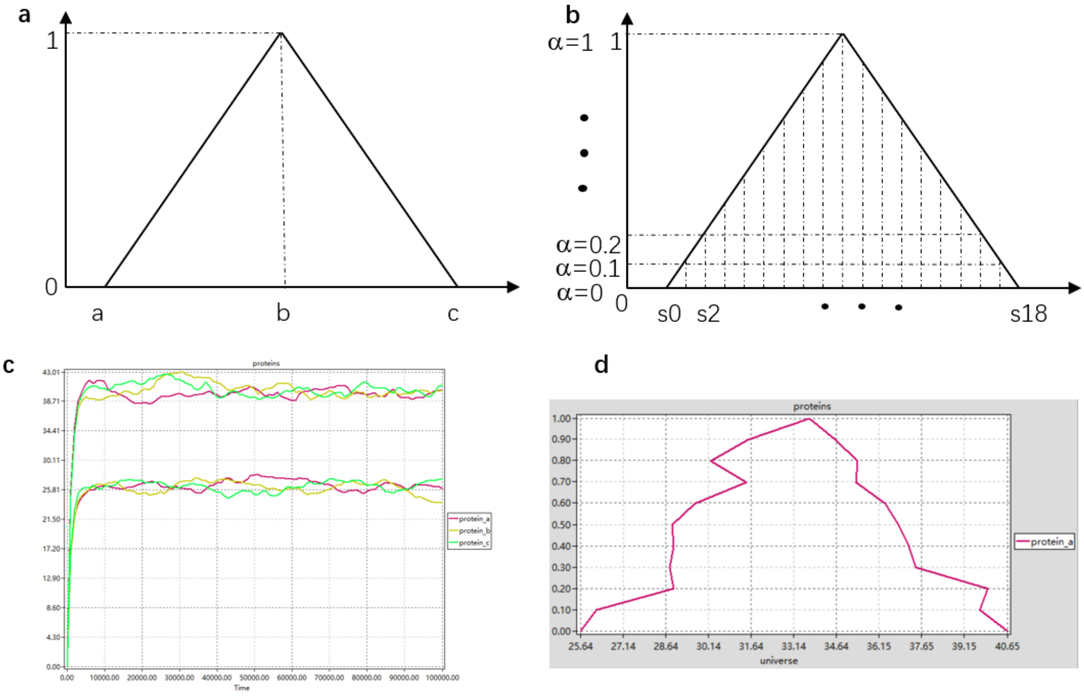


Figure S6. Simulation results of the coloured repressilator model as an FPN.

## 7. Coloured Petri nets - Glossary of basic notions

Coloured Petri nets enjoy a couple of specific notions, which are described as follows.

**Binding**. A coloured Petri net usually has a set of variables that are used in transition guards and arc expressions. To animate, simulate or analyse it, these variables must be assigned values of suitable data types (i.e., colour sets); this is called binding. A legal binding should satisfy the following two conditions: 1) each variable is assigned a value of a suitable colour set, and 2) the guard of the transition should be evaluated to true.

**Colour**. Coloured Petri nets allow tokens to have a (discrete) data value attached to them. This data value is called a colour.

**Coloured function**. A coloured function comprises the following elements: function name, parameter list, function body and return type. Coloured functions can be used to define colour sets, guards or arc expressions.

**Colour set**. A colour set consists of a group of colours with the same data type, e.g., integer, Boolean or string. Each place gets assigned a colour set and may contain tokens coloured with a colour of this colour set; thus, tokens become distinguishable.

**Expression**. An expression is built up from variables, constants, operation symbols and functions. It is associated with a particular colour set; its result is a multiset over this colour set. An expression has to be written in terms of a predefined syntax.

**Folding/Unfolding.** Coloured Petri nets with finite colour sets can be automatically unfolded into uncoloured Petri nets, which then allows the application of all the powerful Petri net analysis/simulation techniques available for the uncoloured counterparts. Vice versa, uncoloured Petri nets can be folded into coloured Petri nets, if partitions of the place and transition sets are given. See Section 9 for more details and examples.

**Guard**. Each transition has a guard, which is a Boolean expression over defined variables. The guard must be evaluated to true for the enabling of the transition. Guards may also occur in arc expressions to specify a group of arcs of specific colours and in the initial marking specification to select a subset of colours. Guards can also be used in rate functions to specify a group of transition instances that satisfy the guards, in order to associate them with different rate functions. Guards are given in square brackets; the trivial guard ‘true’ is usually not shown.

**Multiset.** A multiset is a set in which there can be multiple occurrences of the same element. The number of occurrences of an element is called coefficient or multiplicity. The marking of a coloured place is generally a multiset over the place’s colour set; likewise, the result of arc expressions are generally multisets over the colour set of the adjacent place.

**Place instance**. Each colour of the colour set associated with a place corresponds to an instance of the place.

**Transition instance**. A binding of a transition is an assignment of values to all variables associated with the transition. Each legal binding corresponds to a transition instance.

**Variable**. A variable is an identifier whose value can be changed during the execution of the model. Variables can be used in arc expressions, guards, initial marking specification, and rate functions.

*An example to illustrate the notions of coloured Petri nets.*

The repressilator consists of three identical gene gates [BCP08]; the three genes (called *a*, *b*, and *c*) repress each other in a circular manner. The repressilator can be modelled as an uncoloured Petri net (see Figure (a)) or a coloured Petri net (see Figure (b)); see Key sources table for the repressilator models. In the uncoloured system, the circular connection between the three gene gate instances is indicated by three pairs of logical transitions (given cross-hatched).

The coloured model can be obtained by folding the three identical gene gates of the uncoloured model. On the contrary, the coloured model can be unfolded to the uncoloured model. To do this, each colour of a place is unfolded to a place instance, and each legal binding of a transition is unfolded to a transition instance. See Section 9 for more information about folding/unfolding.

To construct the coloured model, a colour set *GeneSet* can be defined with three colours, *a*, *b* and *c*, to distinguish these three gene gates. Each place gets assigned the colour set *GeneSet*. There can be several tokens of the same colour on a place, and all these tokens on this place define a multiset over the colour set of the place, e.g. 1`all(), which is equivalent to 1`a++1`b++1`c. Each transition has a Boolean guard *true* (not displayed), which has to be evaluated to true in order to enable the transition (which is trivial for the given model). Each arc is assigned an expression, e.g. *x* or *-x* in this model. As a result, a coloured Petri net model (Figure (b)) is built.

Note that we use the UK spelling for 'colour' in the general text, whereas the keywords in the tool platform follow American spelling (such as *colorset* etc.).

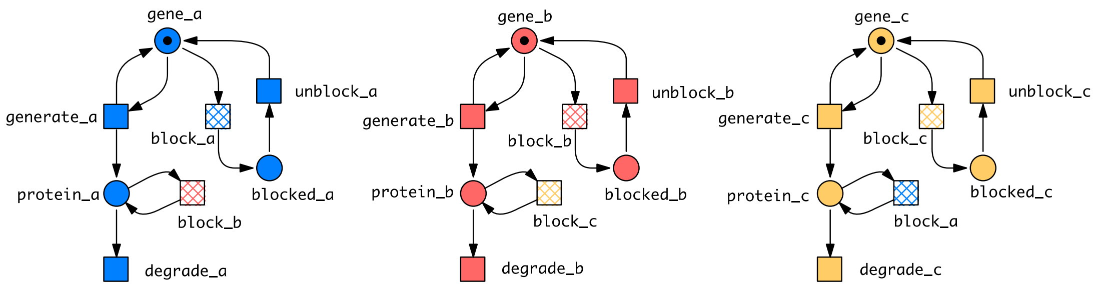


(a) An uncoloured Petri net model for the repressilator [LG13].

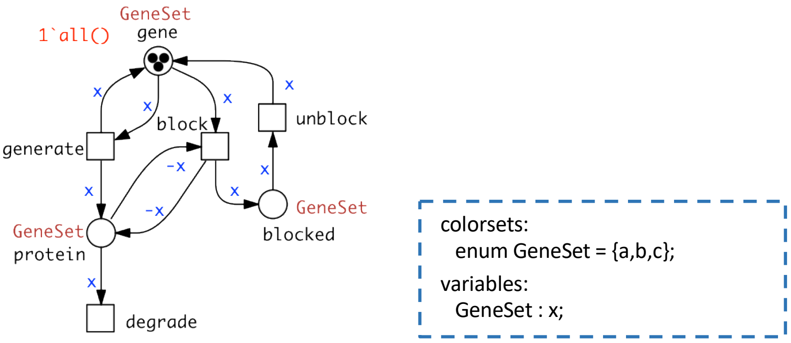


(b) A coloured Petri net model for the repressilator

Figure S7. Petri net model and coloured model of the repressilator. Adapted from F. Liu, M. Heiner, Petri Nets for Modeling and Analyzing Biochemical Reaction Networks, in Approaches in Integrative Bioinformatis, 245-272, 2014, Springer Nature.

## 8. Coloured Petri nets – basic syntax

Coloured Petri nets have an annotation language, which is described in BNF and given in the Manual for coloured Petri nets [LHR12]. Here we briefly describe the basic syntax of the annotation language.

**Data types for defining colour sets.** Two kinds of data types (simple and compound) are available. The simple types can be directly used, while the compound types have to be based on previously defined colour sets.

**Simple types** include dot, int, string, bool, enum and index.

(i) Dot — a singleton set containing only one black colour "dot".

(ii) int — the set of non-negative integers. The declaration syntax: integers separated by comma or dash, e.g. 1,2,3 or 1-3.

(iii) string — the set of all possible strings, which are specified by sequences of printable ASCII characters. The declaration syntax: strings separated by comma, e.g. a, b, c.

(iv) bool — the set of Boolean values {true, false}.

(v) enum — the set of all possible identifiers. The declaration syntax: identifiers separated by comma, e.g. a1, a2, a3.

(vi) index — declares a range between two indexed values, each comprising an identifier and an index specifier. The declaration syntax: index id with [intexp1 – intexp2], e.g., declaring a colour set Philosopher with index phil[1-5].

**Compound types** include product and union.

(i) A product colour set is a tuple of previously declared colour sets. The declaration syntax: defined colour sets separated by commas, e.g., AB can be declared as a product colour set based on A and B, where A and B are declared coloured sets.

(ii) A union colour set is a disjoint union of previously declared colour sets. The declaration syntax: defined colour sets separated by commas, e.g. AB is a union colour set based on A and B.

**Subsets of colour sets** can be defined in the following two ways.

(i) Enumerate the colours that will appear in a subset, separated by comma.

(ii) Use a logic expression to select a group of colours.

**Colour functions**. Each user-defined function contains:

(i) return type, which is a data type or a colour set,

(ii) function name, which is an identifier,

(iii) parameter list, separated by comma,

(iv) function body, which is an expression.

**Operators.**

(i) Unary operators include successor (+), predecessor (-), logical not (!).

(ii) Binary operators include:

a. arithmetic operators: multiplicity (*), division (/), modulus (%), power (^), addition (+), subtraction (-);

b. relational operators: less than (<), less than or equal to (<=), greater than (>), greater than or equal to (>=);

c. equality operators: equal (=), unequal (<>);

d. logical operators: and (&), or (|);

e. other operators: comma (,) that is used in a tuple expression, backquote (`) that is used to separate coefficient and colour, multiset addition (++) that is used to connect two multiset expressions.

**Built-in functions**.

(i) all(), which returns all colours of a colour set.

(ii) abs(), which returns the absolute value of a given expression.

**Expressions**. Each expression is written according to the BNF format given in Appendix A.3 of [LHR12], which can involve constants, variables, and user-defined functions and operators.

## 9. Coloured Petri nets - folding and unfolding

Uncoloured Petri nets and coloured Petri nets are in a folding-unfolding relationship.

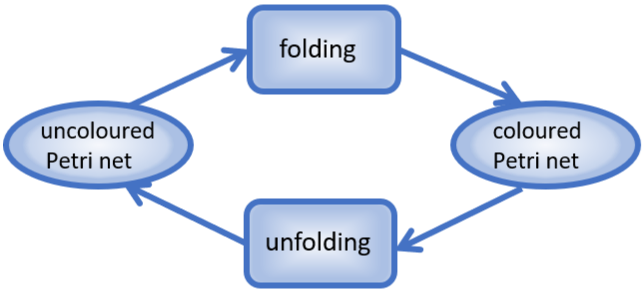


Figure S8. Folding and unfolding.

**Unfolding**. Any analysis or simulation of the coloured model is done on the automatically unfolded version. When simulating a coloured model, the traces of both the uncoloured and coloured places and transitions can be obtained and analysed. In addition, an unfolded version of a coloured model can be exported and opened for inspection. Unfolding applies naming conventions, easing readability: an uncoloured place/transition shares the name of its corresponding coloured place/transition followed by the associated colour(s) separated by underscore(s). For example, unfolding the coloured Petri net p→t given below in (a) generates two places p_1 and p_2, and two transitions t_1 and t_2.

**Folding**. Although unfolding is automated, automated folding is in general unfeasible, and thus the creation of a coloured model from an uncoloured model must be performed by the modeller. Due to the highly compressed nature of coloured models, where the net structure is partially

encoded in colour annotations, modellers should take care to check their coloured model, for example by animation as well as inspection of the unfolded model.

*Examples.* Here we give some illustrative examples regarding folding and unfolding using colour.

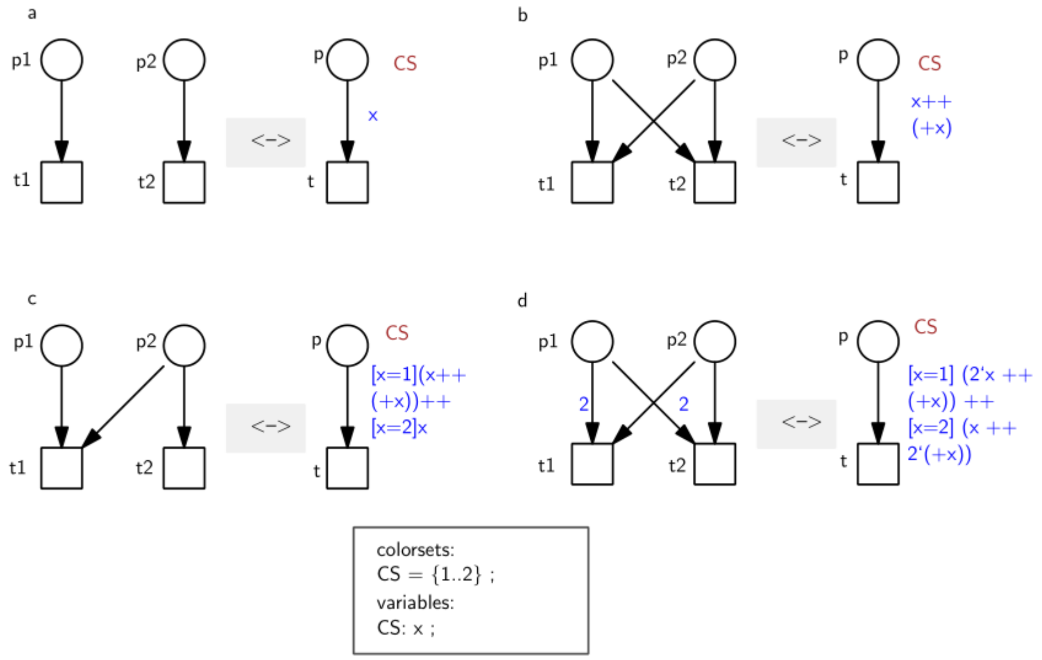


Figure S9. Illustrative examples regarding folding and unfolding using colour [GGH+13]. (© [2013] IEEE. Reprinted, with permission, from [Q. Gao, D. Gilbert, M. Heiner, F. Liu, D. Maccagnola and D. Tree. Multiscale Modeling and Analysis of Planar Cell Polarity in the Drosophila Wing. IEEE/ACM Transactions on Computational Biology and Bioinformatics, March-April 2013.] )

**a.** Two identical uncoloured instances p1→t1 and p2→t2 on the left can be folded into a coloured version p→t on the right, assigning each place to a unique colour, e.g. 1 or 2, thus CS stands for the (ordered) colour set {1,2}. The transition t in the coloured version requires for firing a token from either colour. We create a variable x, the value of which is always an element of CS, thus it can dynamically take any token residing on p at the time of the firing.

**b.** The two uncoloured places p1 and p2 on the left are each preplaces to the uncoloured transitions t1 and t2. This uncoloured system can be folded into the coloured system p→t on the right, using the same colour set CS. In this case the transition t in the coloured version requires for firing a token from both colours at the same time. The annotation on the arc (arc expression) x ++ (+x) stands for this requirement; the operator ++ denotes 'multiset and', and the expression (+x) indicates incrementation by 1 which is applied circularly to the ordered set {1,2} such that (+1) results in 2, and (+2) results in 1.

**c.** The uncoloured place p1 is a preplace to the transition t1and p2 is a preplace to both t1 and t2. This system can be folded to the coloured system p→t on the right, again using the same colour set CS. In this case the transition t in the coloured version requires for firing either a token from both colours at the same time, or just a token from colour 2. The annotation on the arc expression [x=1] (x ++ (+x)) ++ [x=2] x stands for this requirement. This can be read as: 'if the colour of the token is 1 then a token of each colour is required (see (b)), else if the colour

of the token is 2 then only that token is required'. In this case the second occurrence of '++' stands for a sequential choice.

**d.** The two uncoloured places p1 and p2 on the left are each preplaces to the uncoloured transitions t1 and t2, as in Figure (b) except that both transitions require two tokens from p1 and one token from p2. This uncoloured system can be folded into the coloured system p→t on the right, using the same colour set CS. In this case the transition t in the coloured version requires for firing two tokens of colour 1 and one token of colour 2 at the same time. The annotation on the arc expression [x=1] (2`x ++ (+x)) ++ [x=2] (x ++ 2`(+x)) stands for this requirement. This can be read as: 'if the colour of the token is 1 then two tokens of that colour and one token of the other colour are required, else if the colour of the token is 2 then one token of that colour and two tokens of the other colour are required'. Note that in this case, 2`x stands for 2 tokens of the colour assigned to x.

## 10. Encoding space - principles and examples

Encoding space is the first issue to be considered when a multilevel model is constructed with CPNs. Spatial relationships can be encoded in two main ways.

- **Coordinate spatial modelling (i)**: space can be defined in 1, 2 and 3D, and locations denoted by cartesian or polar coordinates represented as colour tuples accompanied by an appropriate arithmetic. For example, a 2D cartesian space can be represented by coordinate pairs of 2 colours e.g., $(x, y)$ in a product relationship.
- **Graph-based spatial modelling (ii):** represents spatial relationships as a directed graph encoded by a set of nodes as colours and set of tuples for the arcs in the graph.

**(i) Coordinate spatial modelling** involves two important aspects: (1) dividing the space into grid cells (for 2D space), each occupying a region such as rectangle or hexagon, or dividing the space into sub-volumes (for 3D space), each occupying a region such as cuboid; (2) determining the neighbourhood relations for a division.

**(i1) Rectangular arrangement in 2D space (Figure (a)).**
Divide a rectangular grid into squares. Each square is addressed by a two-tuple colour $(x, y)$. Different neighbourhood functions can be defined, for instance with four neighbours (top, bottom, left, right (see the four solid lines with arrows)) or eight neighbours (including diagonals (see the four dotted lines with arrows)), and adopting different boundary conditions (absorbing, reflecting, circular) [GHL+13, LBH+14]. See case study: Diffusion in 2D in the main text.

**(i2) Hierarchical rectangular arrangement in 2D space (Figure (b)).**
Use nested colour sets to represent a hierarchically organized system (three levels), which consists of (i) a group of channel clusters (Figure ($b_i$)), (ii) each cluster is further composed of a set of channels (Figure ($b_{ii}$)) [LH13], (iii) a channel. For this, a product colour $(x, y)$ is used to represent the location of a cluster, in which a second product colour $(a, b)$ is used to denote the location of a channel. Thus, a channel has a unique identity $((x, y), (a, b))$.

**(i3) Hexagonal arrangement in 2D space with multiple levels (Figure (c)).**

Use nested colour sets to represent a tissue comprising multiple cells hexagonally packed in a honeycomb formation in order to describe the phenomenon of Planar Cell Polarity (PCP) signalling in *Drosophila* wing [GGH+13]. This model considers three levels: (i) tissue (Figure ($c_i$)), (ii) cell (Figure ($c_{ii}$)), and (iii) compartment. A product colour $(x, y)$ is used to represent each cell on the tissue, and a second product colour $(a, b)$ to describe each compartment. That is, each compartment is distinguished by a nested colour $((x, y), (a, b))$.

**(i4) Cuboid arrangement in 3D space (Figure (d)).**

In [IHA+20] an encoding method of 3D space was given, where each element of the 3D grid is represented as a 3-tuple $(x, y, z)$ and a neighbourhood function in 3D space was given.

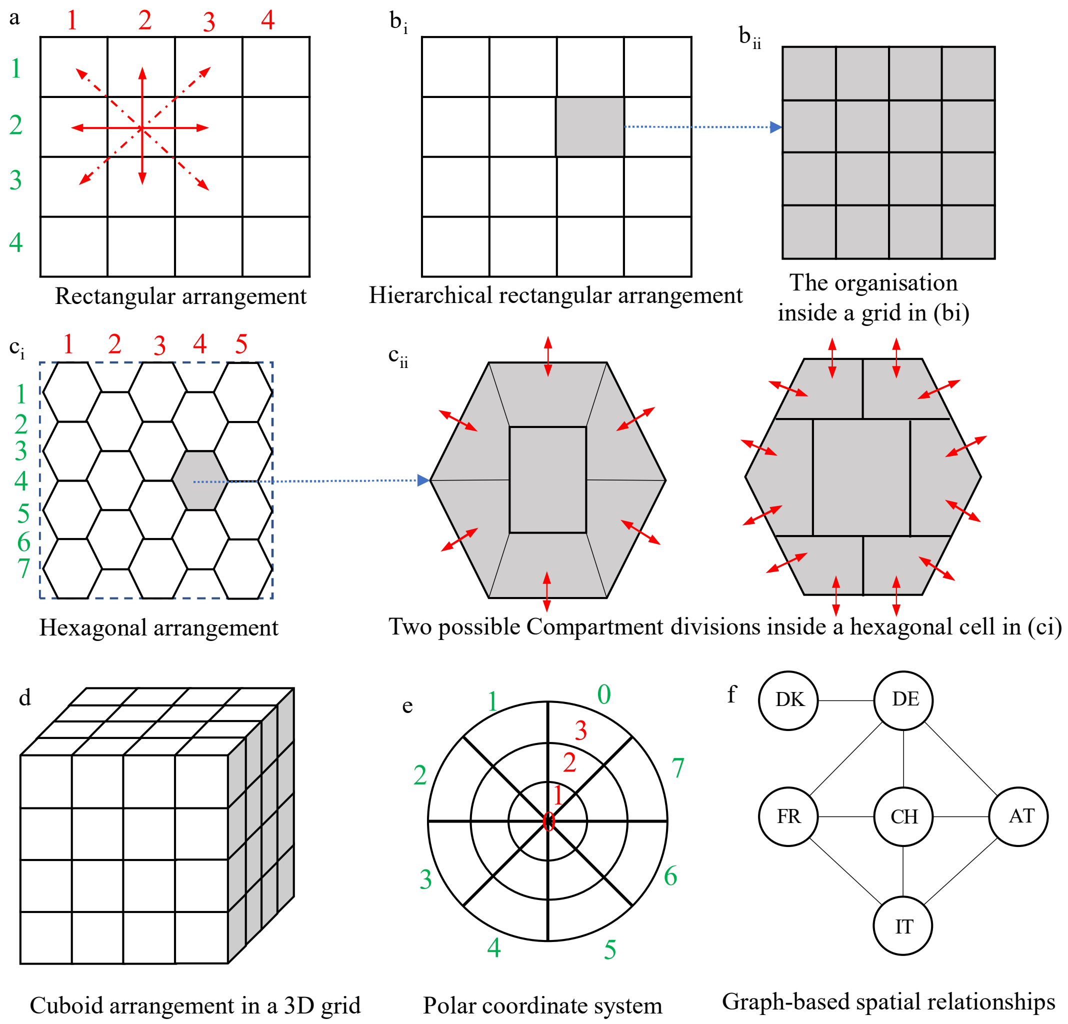


Figure S10. Some examples of space encoding.

**(i5) Polar coordinate systems (Figure (e)).**

In [PGH+15], the encoding of 2D space with the polar coordinate system was given, where each annular section is denoted by a product colour$(x, y)$. Besides, a neighbourhood function in this system was also proposed.

**(ii) Graph-based spatial modelling**. Spatial relationships can easily be represented as graphs, especially where a regular grid of items is not appropriate. For any graph, the set of nodes should be defined as one colour set, and the set of (directed) connecting arcs as a set of tuples defined as subset of the product of the node colour set.

Thus, in the undirected graph example (**Figure (f)**) which encodes the land border connections between six European countries, the set of nodes is {AT, CH, DE, DK, FR, IT}. Undirected arcs are represented by two directed arcs in opposite directions, e.g. tuples (DE, FR) and (FR, DE) for the border between DE and FR. This approach has been used to model the COVID-19 pandemic in Europe [CGH22].

## 11. Multilevel, Multiscale and Multidimensional

**Multilevel modelling** considers dynamic processes at multiple levels (e.g. subcellular, cellular, tissue level) of biological systems.

*Example Multilevel with space*: Planar cell polarity in *Drosophila* wing [GGH+13]; a signalling network, comprising seven compartments in a cell, each having the same signalling network, and the cells are combined to a tissue; see Section 10 for some examples. The input varies the impact of gene mutation on components of the signalling network, while the results are taken from the tissue level.

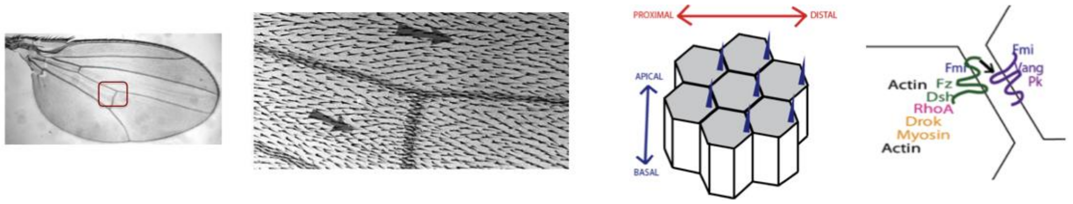


Figure S11. Planar cell polarity in *Drosophila* wing. Some figures are reused from [GGH+13]. (© [2013] IEEE. Reprinted, with permission, from [Q. Gao, D. Gilbert, M. Heiner, F. Liu, D. Maccagnola and D. Tree. Multiscale Modeling and Analysis of Planar Cell Polarity in the Drosophila Wing. IEEE/ACM Transactions on Computational Biology and Bioinformatics, March-April 2013.] )

*Example Multilevel without space*: Transcription of genes to mRNA and translation of mRNA to proteins (e.g. enzymes), creating three levels in modelling.

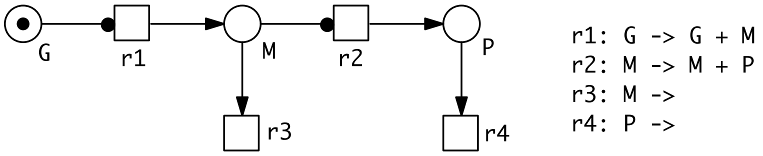


Figure S12. Transcription of genes to mRNA and translation of mRNA to proteins.

In the Petri net:

G - gene, M – mRNA, P – protein

r1 - transcription of gene G into mRNA M

r2 - translation of mRNA M into protein P

r3 - mRNA degradation

r4 - protein degradation

This pattern has been used for instance in the Petri net model for AI-2 production in quorum sensing, see Figure 3 in [GHG+19], where this pattern appears twice.

**Multiscale modelling** incorporates multiple different temporal and/or spatial scales in one model, regardless of whether the model has multiple levels. A multilevel model is not necessarily a multiscale model, and vice versa. However, multiple levels often coincide with multiple spatial and temporal scales.

*Both examples* above are also multiscale.

*Example Multiscale (in time, no space) without multilevel*: cell cycle at a single abstract level, where activity is reduced to cell division, yields an HPN, comprising continuous and stochastic/immediate transitions; based on [HSH13].

In the HPN, the continuous transition *grow* acts a source to feed *v* continuous place representing the volume of the cell. The stochastic transition *divide* will halve the volume of *v* as soon as it is triggered by the immediate transition *go* which puts a token on the discrete place *on* when the threshold *thr* has been reached. This effect is achieved by the two self-modifying arcs between the transition *divide* and the place *v*. The threshold *thr* determines the value above which the contents of the place *v* are divided (i.e. the value of *v*). The value at which *v* is divided is always equal or greater than *thr* by some value determined by the stochastic rate (*k_div*v*on*, where '*on'* is 0 or 1) of divide, never less than this.

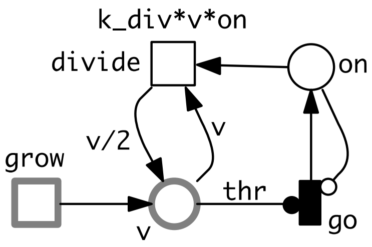


Figure S13. Cell cycle model.

Hybrid simulation traces: (Top) values of places – *v* (red): continuous, *on* (blue): discrete. (Bottom) rates of transitions: *go* (blue) always precedes *divide* (green), grow (red).

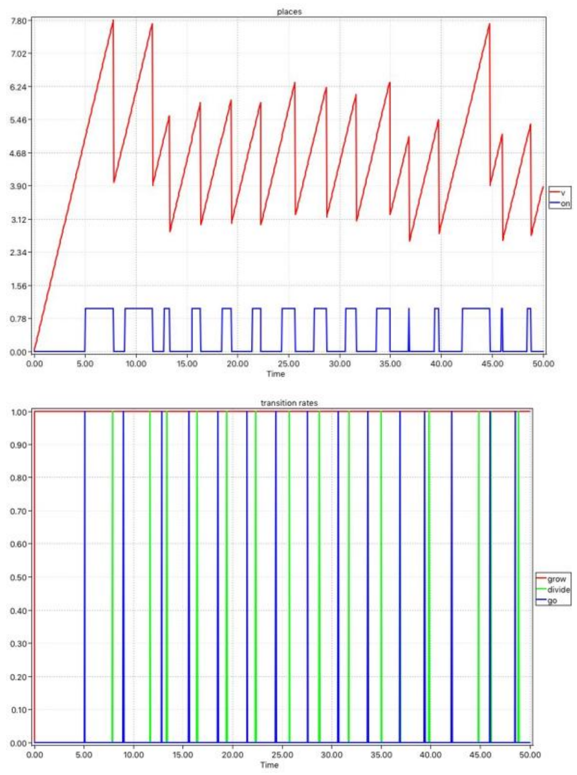


Figure S14. Simulation results of the cell cycle model.

*Example Multiscale (in space, but not in time) without multilevel*: In the DNA walker example [GHR18] there is both short distance – local movement between anchorages – and long distance – final anchorages must be sufficiently far apart to obtain reliable results. However, the system has only one time scale, that of movement between anchorages ('walking'), and there is only one physical level (the circuit layout).

*Example Multiscale (in both space and time) without multilevel*: In the phase variation example [PGH+15], space is present in terms of both local movement as well as global patterns in the bacterial colony. Time is described both in terms of local movement of individual bacteria as well as long time periods required for the emergence of global patterns.

**Multidimensional modelling.** Biological models can also be categorised according to their spatial multidimensionality, besides any multilevel or multiscale aspects. For example, when studying reaction–diffusion processes, we can model this phenomenon in one-, two- or three-dimensional space; see e.g. [LBH+14] – for 1D, 2D (Brusselator), or [IHA+20] for 3D example (calcium channel).

## 12. Example – Delta Notch signalling pathway

The Notch signalling pathway regulates cell division, fate and death in all metazoans [Kop12]. In the Delta Notch pathway, Notch as a transmembrane receptor (NT) transduces signals and interacts with the transmembrane Delta ligands (D) on neighbouring cells. This binding leads to cleavage of Notch at site 2. Subsequentially, another cleavage of Notch at site 3 occurs, causing the release of

the Notch intracellular domain (NI). NI then travels to the nucleus and activates some target genes, which have an inhibiting effect on the generation of Delta in the same cell.

The Delta Notch signalling pathway functions during diverse developmental and physiological processes, e.g., (i) lateral inhibition that makes different cells adopt distinct fates [CMM+96], [SLL+10] and (ii) boundary formation between two populations of cells, e.g. *Drosophila* hindgut [TYY+02], [LYS+22].

In the former case, the lateral inhibition between adjacent cells leads to fine-grained spatial patterns. At steady state, there usually appear two distinct behaviours, corresponding to two fates: (i) the primary fate due to high D, but low NI, and (ii) the secondary fate due to low D, but high NI.

In the following figure, we illustrate the interaction of two cells.

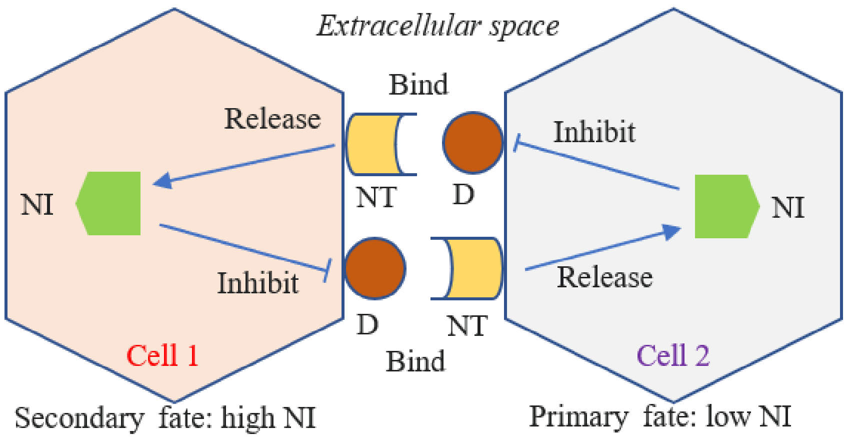


Figure S15. The interaction of two cells

Based on the diagram above, we construct the following SPN model of two cells (see Key sources table for the model). Here, the transitions *Generate* represent the production of D or NT, and the transitions *Degrade* represent the degradation of D, NT or NI. The transitions *Bind* model the binding of D to NT. The solid arcs give the flow of these reactions, while the dashed modifier arcs correspond to the inhibitory effect of NI on the production of D in the same cell, which is achieved by the rate function of the transitions *Generate2*.
The rate functions and constants (see Figure (b) and (c)) are adapted from [CMM+96]. The rate function of the transitions *Bind* is an increasing function of D from neighbouring cells. The rate function of the transitions *Generate2* is a decreasing function of NI of the same cell. Degradation rates (*Degrade1*, *Degrade2* and *Degrade3*) follow mass action kinetics, while the sources (*Generate1*) produce at constant rate.

a

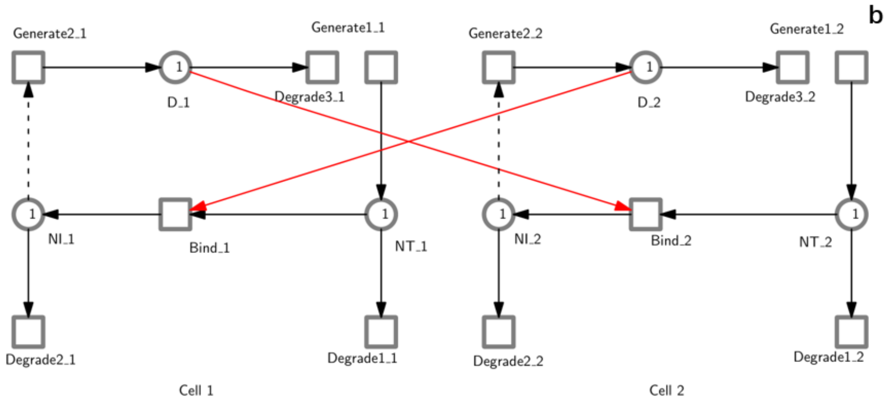


b

```
constants:
  double d1=0.1;
  double d2=0.1;
  double d3=0.1;
  double kd=1;
  double kc=1;
  double kn=1;
  double k1=0.01;
  double k2=100;
```

c

| Transition | Rate (Cell 1) | Rate (Cell 2) |
|---|---|---|
| Generate1 | kn | kn |
| Degrade1 | MassAction(d1) = d1*NT_1 | MassAction(d1) = d1*NT_2 |
| Bind | kc*D_2*D_2/(k1+D_2*D_2)*NT_1/(k1+NT_1) | kc*D_1*D_1/(k1+D_1*D_1)*NT_2/(k1+NT_2) |
| Degrade2 | MassAction(d2) = d2*NI_1 | MassAction(d2) = d2*NI_2 |
| Generate2 | kd/(1+k2*NI_1*NI_1) | kd/(1+k2*NI_2*NI_2) |
| Degrade3 | MassAction(d3) = d3*D_1 | MassAction(d3) = d3*D_2 |

Figure S16. Petri net model of two cells.

## 13. Snoopy - Petri nets elements

Table S2. Elements of Petri nets in Snoopy.

| Type | Icon | Name | PN ColPN | XPN ColXPN | SPN FSPN ColSPN ColFSPN | CPN FCPN ColCPN ColFCPN | HPN FHPN ColHPN ColFHPN |
|---|---|---|---|---|---|---|---|
| Place | 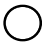 | Discrete | √ | √ | √ | | √ |
| | 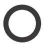 | Continuous | | | | √ | √ |
| Transition | 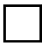 | Discrete/ stochastic | √ | √ | √ | | √ |
| | 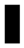 | Immediate | | | √ | | √ |
| | 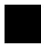 | Deterministic | | | √ | | √ |
| | 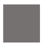 | Scheduled | | | √ | | √ |
| | 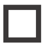 | Continuous | | | | √ | √ |
| Arc | 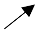 | Standard | √ | √ | √ | √ | √ |
| | 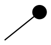 | Read/ test | √ | √ | √ | √ | √ |

| | | | | | | | |
|---|---|---|---|---|---|---|---|
| | | Inhibitor | √ | √ | √ | √ | √ |
| | | Reset | √ | √ | √ | | √ |
| | | Equal | √ | √ | √ | | √ |
| | | Modifier | | | √ | √ | √ |
| Logical node | | Logical place | √ | √ | √ | √ | √ |
| | | Logical transition | √ | √ | √ | √ | √ |
| Macro node | | Macro place | √ | √ | √ | √ | √ |
| | | Macro transition | √ | √ | √ | √ | √ |

*(i) Different net classes may have distinct groups of elements.*

(ii) *Connection rules in HPN and ColHPN:*

- Discrete places cannot be connected with continuous transitions via standard arcs.
- Continuous places cannot be tested via equal arcs, neither by discrete nor continuous transitions.
- Continuous transitions cannot use reset arcs.

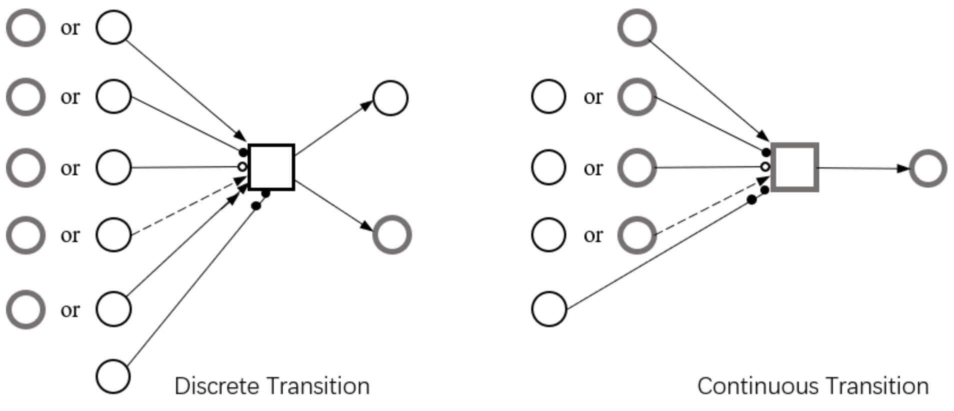


Figure S17. Connection rules in HPN and ColHPN.

*Remarks:*

- Discrete transitions include stochastic, immediate, deterministic and scheduled ones.
- Arc weights have to be natural numbers (integer constants) for discrete transitions, and can either be natural numbers or real numbers (double constants) for continuous transitions.
- Self-modifying arcs enjoy the same graphics as standard arcs, but the arc weight is a marking-dependent expression, see Section 3.

## 14. Snoopy - basic features

Open Snoopy. Selecting the *File* menu at the top reveals a pull-down list with the following options.

**New** asks for the type of Petri net to be constructed (*Select a document template*) and then creates an empty canvas for the new Petri net, called *unnamed* followed by an extension reflecting the net class (such as pn, xpn, spn, …) The graph elements and declarations, offered in movable sub-windows, generally depend on the Petri net type. Several Petri nets, possibly of different type, can be open simultaneously. The windows with the graph elements and declarations are automatically adjusted to the active net window.

**Open** existing file shows all files with an extension known to Snoopy in the selected folder.

**Close Window** closes the active canvas, but keeps all other windows open. If there are unsaved changes, a warning pops up asking whether they should be saved or ignored.

**Save** saves the Petri net in the active canvas. If it is a new net and no name has been assigned yet, Snoopy asks to specify a name.

**Save As** permits to change the file name upon saving, which creates a copy of the Snoopy file.

**Save As and Sign**

*Remark:* Files are stored in a compressed format (zip), if this option has been permitted in the global preferences.

**Page Setup** permits to configure the printer; set the paper size large enough for the Petri net to be printed.

**Print** opens the standard printer menu; select *save as pdf* to obtain high-resolution figures of a Petri nets.

**Print Preview** permits to check whether the paper size has been set large enough to show the entire Petri net.

**Export** functions include:

(i) export to other net classes,

(ii) export to exchange file formats like ANDL, CANDL and SBML,

(iii) export to other file formats like eps, Latex, and CSV.

(iv) Export of a coloured model to an uncoloured one involves unfolding. Afterwards, the unfolded model can be opened and checked against the coloured origin.

(v) It is also possible to export just the structure of a coloured model (without unfolding), which is useful, for example, to extract a component from a system model.

**Import** functions include the reading of exchange file formats like ANDL, CANDL and SBML. Upon import, an SBML file can be converted into a Petri net, stochastic Petri net or continuous Petri net.

There is a list of recently opened files at the bottom of the file pull-down list, providing easy access to re-open them again.

*Remark:*

All loading and saving of files is reported in the log window, with date, file name and full path.

## 15. Snoopy - other useful features

(1) Selecting the ***Edit*** menu on the top reveals a pull-down list; among the options offered are:

**Edit selected elements** The attributes of all net elements can not only be edited individually, but also in bunches. For this, select first all net elements to be edited, then go to this option, and change in the dialogue all attributes which should be equally applied to all selected elements. This feature permits, among others, to easily colour subnets.

**Transform Shapes** supports layout adjustments by rotating, vertical flipping or horizontally mirroring of the selected subnet.

**Layout**. Snoopy offers three types of layout algorithms: FMMM, Planarization, and Sugiyama.

**Sort Nodes** permits to order the elements in each category (place, transition, … constants, functions) either by name, ID or manually, in ascending or descending order.

**Check net**. Checks the integrity of the net, looks for duplicated nodes and checks its syntax. The checking result will be given in the log window. It also supports the repositioning of all attributes (such as node names).

(2) Selecting the ***View*** menu on the top reveals a pull-down list; among the options offered are:

**Zoom in/out** specifically helpful for larger networks.

**Net information** reveals some statistics including the number of places, transitions, arcs, each split according the various types available in the given net class, and the total token count.

**Show Attributes** permits to uniformly switch on/off the showing of the individual attributes for every type of net elements; e.g., whether transition names should be shown or not. Additionally, the show attribute can be set individually for every net element; e.g., the name of some transitions could be shown and for other transitions not.

(3) Selecting the ***Search/Search nodes*** menu opens the search dialogue, where places or transitions can be searched in terms of their complete or partial names or ID. Do not forget to select the appropriate type of nodes.

(4) Selecting the ***Extras*** menu on the top reveals a pull-down list; among the options offered are:

**Duplicate into logical nodes** creates copies of the selected nodes, one for each adjacent arc, and indicates all copies as logical. This option permits the fine tuning of the graphical layout by exploiting Snoopy's feature of logical nodes; works equally for places and transitions.

**Merge logical nodes** merges among the selected nodes those places or transitions, which are equally named.

(5) Selecting ***Preferences*** opens a window offering numerous options to configure general settings for Canvas, Animation, Elements and Fonts, each of them specific for every net class. Among them are:

**Grid spacing** to assist a neat net layout, a grid can be shown on the drawing canvas. The spacing can be flexibly adjusted, e.g. to the chosen node size.

**Show Grid** can be switch on/off.

**Snap elements to grid** can be switch on/off.

**Window size (of the canvas)** can be adjusted, specifically increased, in order to cope with the size of the net to be constructed.

**Enable file compression** Snoopy's file format builds on XML technology. So, the files holding larger nets may become large. To save space, these nets can be automatically compressed upon saving, which then also includes the automatic decompression upon opening.

(6) **Check and delete unused declarations**. The iterative process to construct a net which finally conforms to the expected behaviour may result into a net, where certain declarations of constants, colour sets, variables, etc. are actually not required anymore. This option permits to identify those unused declarations and to delete them, if appropriate. To make use of this feature, make a right-mouse click in the declaration sub-window on any node in the declaration tree, and all nodes in the selected subtree will be check for unused declarations, which then can all or selective be deleted.

## 16. Snoopy - animation configuration

Animation is supported for all discrete Petri net classes, i.e. qualitative and stochastic Petri nets, either coloured or uncoloured ones. The animation dialogue and features slightly depend on the net class.

**To animate QPN, XPN** follow these steps:

1. Open the net, and open the animate window (*View -> Start Animation Mode*).
2. Set initial marking: A left mouse click on a place increases the token number by 1, a right mouse click decreases the token number by 1. Alternatively, go to *Marking overview* to set a specific marking.
3. Choose execution mode:
   - ***Manual mode*** – A left mouse click on a transition triggers the firing of this transition, if it is enabled; otherwise the alert appears "This transition is not enabled!".
   - ***Automatic mode*** – Clicking directly on the control panel in the animation window, either *Step forward* or *Play forward*, leaves the decision of which transition to fire to the animation algorithm, which works as follows.

     First, the set of all enabled transitions is determined. Next, one of three strategies is applied to choose the transition(s) to be fired in the next execution step among all enabled transitions.

     - *Single step* – one single enabled transition is randomly chosen.
     - *Intermediate step* – an arbitrary subset of concurrently enabled transitions is randomly chosen.
     - *Maximal step* – a maximal set of concurrently enabled transitions is randomly chosen.

     Go to *Options* to select the strategy to apply; the default is *Intermediate*.

4. There is a step counter indicating the number of executed steps in the current trace, which is actually a random walk through the state space.

These steps can be played backwards or stepped backwards, and can also be written to a file by *Export,* either as a (not necessarily consecutive) sequence of markings (extension: mseq) or as a (always consecutive) sequence of transition steps (extension: tseq). The export file is written while running an animation, thus the file has to be specified before starting the animation. Sequences of transition steps can be loaded again by *Import.* In this way, special traces of interest can be saved or even synthesized by other tools.

5. Before leaving the animation mode, press *Keep marking* or select *Always keep marking when closing* to keep the latest marking reached; otherwise the marking is automatically reset to the one before opening the animation mode.

*Remarks:*

- A set of transitions is *concurrently enabled* at a given marking, if the marking provides sufficient tokens for the simultaneous firing of all transitions in this set.
- Intermediate and maximal step can be combined with *auto-concurrency; i*f selected, a transition can fire concurrently to itself. By default auto-concurrency is disabled.

**To animate ColPN, ColXPN** follow the steps for QPN and ColXPN, with the following differences:

- When varying the initial marking, select the box *Choose random colors* if the colour of the tokens to be added does not matter; otherwise a dialogue appears asking for the colour of the token.
- For the manual mode, additionally select whether the binding dialogue should be shown: *never*, when there are *more than one binding* or *always*.
- Auto-concurrency is not supported.

**To animate SPN or ColSPN** follow the steps above and bear in mind: Animation in automatic mode considers the firing rates; thus, the marking set, function set, parameter sets etc. can be selected, before starting the animation.

## 17. Snoopy - simulation configuration

**Model configuration** During modelling, different sets of initial states (markings), rate functions and constants and their groups (including kinetic parameters) can be defined, and one of each has to be chosen for a given experiment. In contrast to all following categories, the options to choose from do not depend on the simulation type.

**Simulator Configuration**

1. *Interval start* – The simulation starts always at time 0, but simulation data are only recorded beginning with the given start time.
2. *Interval end* – The simulation runs until the specified end time, if no dead state is encountered.

3. *Interval splitting* – The number of output steps in the specified simulation interval defines the output grid resolution of the recorded simulation data.
4. *Simulator* - Choose simulation algorithm, for the available options.
5. *Properties* - Set properties for the simulation algorithm; the options to choose from may depend on the simulation algorithm.

   *Stochastic simulation:*

   - Select if results should be visualized on the fly, i.e., while the simulation is running, which of course increases the runtime (default: on).
   - Set the refresh rate for the visualization of simulation results (default 5,000).
   - The number of simulation runs to be averaged (default: 1).
   - The number of parallel threads to be deployed (default 1).

   *Deterministic simulation:*

   - Set initial step size (default 0.1) or chose to automatically calculate initial step size.
   - Check the results for negative values.
   - Reduce generated ODEs.
   - Specify relative/absolute tolerance.

   *Hybrid Simulation:* Additionally to the stochastic and deterministic simulation options, choose among the following synchronisation principles.

   - *Static* – Each transition type is kept as it has been determined by the modeller.
   - *Dynamic* – The types of stochastic and continuous transitions are adjusted on the fly by evaluating the current transition rates.
   - *Continuous* – The entire net is considered as CPN and thus simulated continuously. Any stochastic transition is automatically converted to a continuous one.
   - *Stochastic* – The entire net is considered as SPN and thus simulated stochastically. Any continuous transition is automatically converted to a stochastic one.

**Import/export details**

- *No export* – No data are written while the simulation is running. Simulation results can be written later, after visual inspection using result viewers.
- *Direct export* – The time series data of the selected places or transitions (as determined by the chosen result viewer) are directly exported.
- *Single trace export* (only SPN, ColSPN) – The result of every simulation run is exported separately.
- *Exact trace export* (only SPN, ColSPN)– Every change of the marking of the selected places or the rates of the selected transitions at any time point is exported.
- *Properties* - Specify the file name, the spacer to be used (default: comma), and if the output is to automatically compress (zip) when writing the simulation data (table).

- *Load Data* – Previously written simulation data can be reloaded for visual inspection using the result viewers defined. Do not forget to have the corresponding Petri net open.
- *Save ODE (only CPN, ColCPN)* – The ODEs automatically generated for the deterministic simulation are written as plain text to the specified file.

**Views** Simulation results are shown be help of result viewers. Several result viewers can be created (*Add*, *Remove*, *Rename*, …) and be opened simultaneously to switch conveniently between different views of the simulation results. Each viewer can be configured independently.

- *Viewer type*, select between:
  1) *Table* – Each column represents a selected place (transition) and each row shows the averaged marking (firing times) at one output step point.
  2) *xyPlot* – Each curve stands for one selected place (transition); the x-axis shows the time and the y-axis the number of tokens (firing times).
  3) *Histogram* – Shows the frequency distribution of the numerical data. The total range of all values is divided into a series of intervals (bins) and then it is counted how many values fall into each interval, with interval width = (max value – min value)/round(sqrt(number of values)).
- *Edit* – Permits to configure the viewer; the options depend on the viewer type.
- *Export* – Simulation results can be saved (tables as csv files, plots and histograms as csv or, e.g., png); to configure them, go to Edit.
- *Change X Axis* – Permits to choose for xyPlots between time, place, or transition.
- *Edit Node List* – Each view is characterised by a set of places (transitions), either uncoloured or coloured ones, which can either be selected manually or using regular expressions.
- *Hide Node List* – Switches off the window to select/unselect the items to view (among those which have been chosen via *Edit Node List*).
- *Refresh* – Triggers a refresh of the visualization from the simulation data produced most recently.
- *Disconnect* – Toggles between automatic refresh on/off, which permits to keep the results from one run to be compared with the results of other runs.
- *Close* – Closes this result viewer, but keeps all other result viewers and the simulation window open.

**Start Simulation** Having started the simulation, the progress bar indicates how far the simulation has gone until now, and the time consumed by the simulation is displayed, too.

## 18. Charlie's Graphical User Interface

Charlie's GUI basically comprises three separate windows.

**Left -** main control panel: the bars for the various analysis engines with their options can be independently opened and closed. The last bar *net properties* shows a summary of the results obtained so far; green stands for YES (the property holds) and red for NO (the property does not hold). Hovering with the mouse over a specific property reveals a tool tip giving the full name of that property; see Section 19 – Section 21 for details.

**Middle -** protocol window: essential analysis results are logged, together with the rules applied; here: *"There is no dead state, because the net is homogenous and the STP holds"* (see red box). When the Charlie session is closed, the log is written to a file.

**Right -** analysis thread window: The analyses run as parallel threads; green indicates that a run has finished; unfinished runs can be stopped or cancelled.

*Remarks:*

Charlie provides more analyser engines than we use in this protocol. Among others, it also performs dynamic analyses of a finite state space, including LTL and CTL model checking. However, the state space is explicitly encoded (in contrast to Marcie, exploiting symbolic data structures), and thus Charlie can generally not compete with Marcie's performance. In more general terms, we do not recommend to use Charlie's dynamic analyses beyond a state space of 500,000. For more details see **[**BHM11**].**

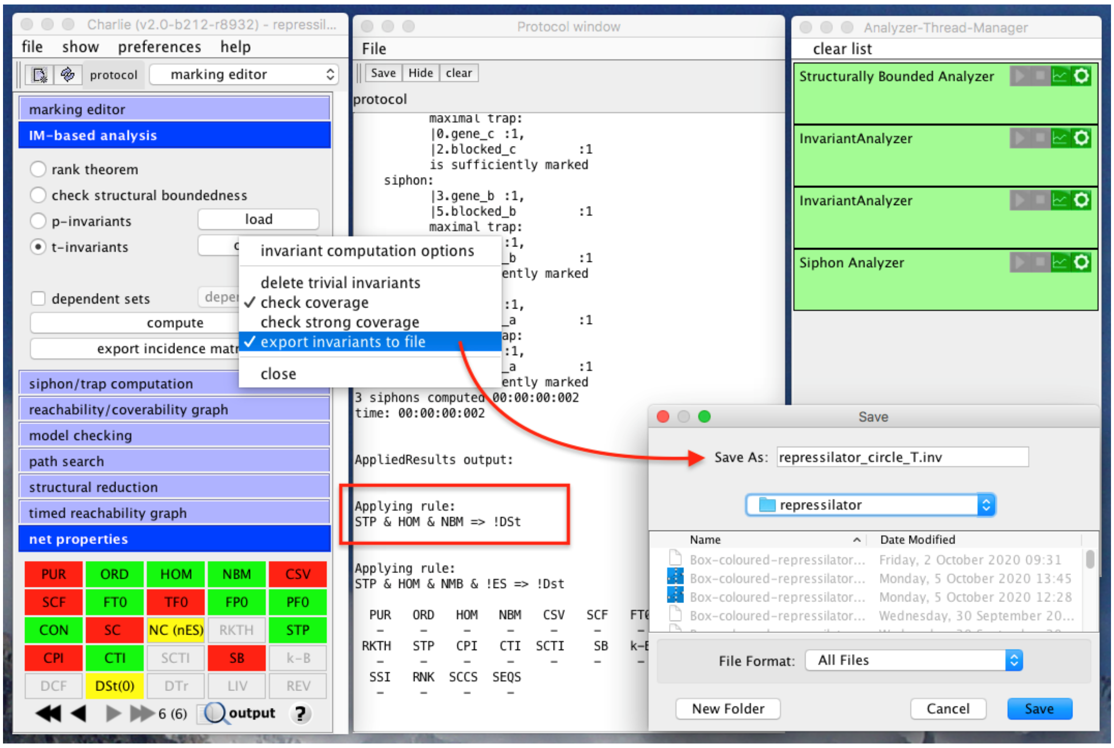


Figure S18. Charlie's interface.

## 19. Behavioural properties

There are three *orthogonal general behavioural properties* that are usually explored first to gain some insights into the behaviour of a Petri net. The acronyms given in brackets are those used by Charlie, see Section 18.

**Boundedness.** A place is said to be *k-bounded (k-B),* or bounded for short, if the maximal number of tokens on this place is bounded by a constant $k$ in all reachable states. A Petri net is $k$-bounded, if all its places are $k$-bounded.

*Interpretation:* Boundedness precludes unrestricted increase of token numbers (representing the amount of a biochemical species) and ensures a finite state space.

**Liveness.** A transition is said to be *live (LIV)* if, whatever happens, it is always possible to reach a state where this transition is enabled. A Petri net is live, if all transitions are live.

*Interpretation:* In a live net, all reactions are able to contribute to the net behaviour forever and are thus forever active; or the other way around: none of reactions will be blocked forever.

**Reversibility.** A Petri net is said to be *reversible (REV)* if the initial state can be reached again from each reachable state. This obviously involves the reversibility of every reachable state.

*Interpretation:* A reversible net has the capability of self-reinitialization; thus it is always capable of returning to its initial state – every behaviour observed so far at any point can be observed again.

Further behavioral properties of general interest include:

**Dead state.** States where no transition is enabled are called *dead states (DSt)*. A live Petri net does not have dead states, but freedom from dead states does not ensure liveness.

*Interpretation:* Dead states are terminal states of the system behaviour; all reactions are blocked and no further progress is possible; also known as a *false equilibrium*.

**Dynamic conflict.** It may happen that two transitions are enabled, but the firing of one transition disables the other one. Such a situation is called a *dynamic conflict (DCF)*.

*Interpretation:* The occurrence of dynamic conflicts indicates alternative (branching) system behaviour, e.g. alternative pathways; decisions between alternatives are taken non-deterministically.

Further *special behavioural properties* can sometimes be expressed by the reachability/unreachability of specific states, but in most cases these call for the use of model checking techniques (see Section 23).

Generally, the decision of behavioural properties requires the construction of the state space. However, there are some techniques from Petri net theory, which can permit conclusions about behavioural properties by just exploring the net structure; see Section 20 and Section 21.

## 20. Elementary graph properties

Elementary graph properties can be determined just by analyzing the net structure and do not depend on the initial state. The computational load for their decision is negligible, thus they are immediately decided when calling Charlie. They usually reflect the modeling approach, so they may be used for preliminary consistency checks to preclude production faults when constructing a Petri net. Occasionally they permit on their own conclusions about behavioral properties (see Section 19). The acronyms given in brackets are those used by Charlie (see Section 18).

**Pure.** A Petri net is *pure (PUR)* if there are no two nodes connected in both directions. This precludes read arcs. In this case, the net structure is fully represented by the incidence matrix, which is used for the computation of invariants (see Section 21).

**Ordinary.** A Petri net is *ordinary (ORD)* if all arc weights are equal to 1. This includes homogeneity (see next property). A non-ordinary Petri net cannot be live and 1-bounded at the same time.

**Homogeneous.** A Petri net is *homogeneous (HOM)* if all outgoing arcs of a given place have the same multiplicity.

**Conservative.** A Petri net is *conservative (CSV)* if all transitions add in total exactly as many tokens to their post-places as they subtract from their pre-places, or briefly, all tokens are preserved under any transition firing.

A conservative Petri net is structurally bounded, that is, bounded for any initial state (where bounded refers to a behavioural property, see Section 19) and in this case the behavioural property can be simply determined by structural analysis.

**Static conflict free.** A Petri net is *static conflict free (SCF)* if there are no two transitions sharing a pre-place. Transitions involved in a static conflict may compete for the tokens on shared pre-places. Thus, static conflicts indicate situations where dynamic conflicts, that is, non-deterministic choices, may occur in the system behavior. However, it depends on the token situation whether a dynamic conflict (DCF) does actually occur. There is no non-determinism in SCF nets.

**Boundary nodes.** Nodes which have either no pre-node or no post-node are called boundary nodes. They exist as four types.

- *Input transition*: a transition without pre-places (FT0),
- *Output transition*: a transition without post-places (TF0),
- *Input place*: a place without pre-transitions (FP0),
- *Output place*: a place without post-transitions (PF0).

Boundary nodes typically model interconnections of an open system with its environment. A net without boundary nodes is self-contained, it is a closed system. Thus, it needs a non-clean initial marking to become live.

A Petri net with input transitions is unbounded, because the firing of input transitions does not depend on any precondition. A Petri net with input places is not live because the tokens on an input place are sooner or later used up. In fact, a net with boundary nodes cannot be strongly connected, and thus not both bounded and live.

A place with neither pre-transitions nor post-transitions is a trivially isolated net, and can represent a biochemical entity which is not involved in any reaction. A transition with neither pre-places nor post-places likewise is a trivially isolated net, but has no obvious meaning.

**Connected.** A Petri net is *connected (CON)* if it holds for every two nodes *a* and *b* that there is an undirected path between *a* and *b*. Disconnected parts of a Petri net cannot influence each other, so it is advisable to analyze them separately.

**Strongly connected.** A Petri net is *strongly connected* (SC) if it holds for every two nodes *a* and *b* that there is a directed path from *a* to *b*. Strong connectedness involves connectedness and the absence of boundary nodes. This is a necessary condition for a Petri net to be bounded and live.

**Net structure class.** We distinguish the following classes of net structures (NC): state machine (SM), synchronisation graph (SG), free choice (FC), extended free choice (EFC), and extended simple (ES): see [HGD08] for details.
The net structure class is specifically of interest if the siphon-trap property holds (see Section 21), because the conclusions w.r.t. liveness (Section 19) depend on the net structure class.

## 21. Basic notions of structural analysis

We recall the basics of two structural analysis techniques, which have been proved to be useful for BioModel Engineering. Both techniques are generally computationally more expensive than the elementary graph properties (see Section 20).

**Invariants** are sets of nodes, which stand for some invariant behavioural property. Invariants induce subnets, comprising the nodes belonging to the invariant and all pre- and post-nodes. These subnets, which may overlap, may be used for modularization, validation, or reduction of the model. We distinguish two types of invariants: P-invariants and T-invariants.

**P-invariant.** A P-invariant specifies a set of places over which the weighted token count keeps constant whatever happens in the Petri net. So, a place belonging to a P-invariant is bounded.
*Interpretation:* In metabolic networks, P-invariants often correspond to conservation laws in chemistry, reflecting substrate conservation, while in signal-transduction networks P-invariants often correspond to the possible states of proteins. Each P-invariant defines a mass/token-preserving subnet.

**Covered with P-invariants.** A net is *covered with P-invariants (CPI*), if each place belongs to a P-invariant.
*Interpretation:* A place covered by a P-invariant is bounded; vice versa, a place not covered by a P-invariant is either unbounded (unlimited token increase) or loses tokens which are never replenished.

**T-invariant**. A T-invariant specifies a multiset of transitions; it can be interpreted in two different ways. The multiset either says how often a transition has to fire to obtain a cycle in the system behaviour, or the multiset gives relative firing rates, keeping the Petri net in a steady state; see [Hei09] for details.

*Interpretation:* T-invariants are often identified with elementary functional modules or sub-pathways; the composition of their behaviour yields the total system behaviour. Each T-invariant defines a state-reproducing subnet. Any steady state behaviour is a linear combination of T-invariants.

**Covered with T-invariants.** A net is *covered with T-invariants (CTI*), if each transition belongs to a T-invariant.

*Interpretation:* A reaction not covered by a T-invariant cannot be involved in any steady state behaviour.

*Examples:* P-invariants and T-invariants are illustrated in Section 22.

**Siphon**. A non-empty set S of places of a Petri net is called a siphon if every transition that fires tokens onto a place in S also has a pre-place in this set; that is, the set of pre-transitions of S is contained in the set of post-transitions of S. A set of places is called clean, if none of the places caries a token.

A clean siphon cannot get tokens again, and then all its post-transitions are dead.

*Interpretation*: Reactions with precursors which are part of a clean siphon will never be able to occur. A system in a dead state (see Section 19) has a clean siphon. Generally, a clean siphon is not desired in biological systems because there will be dead parts in the network.

**Trap**. a non-empty set Q of places of a Petri net is called a trap if every transition that subtracts tokens from a place of the trap set also has a post-place in this set; that is, the set of post-transitions of Q is contained in the set of pre-transitions of Q. Post-transitions of a trap always return tokens to the place set. Therefore, once a trap contains tokens, it cannot become clean again.

*Interpretation:* In biological systems, a trap indicates an irreversible accumulation of compounds. It depends on the application whether this effect is desired or should not occur [ZOS03].

**Siphon-trap property (STP)**. Siphon and trap are closely related, but contrasting notions. When they come on their own, we usually get undesirable behaviour. However, both notions have the power to perfectly complement each other. A Petri net satisfies the siphon trap property (STP) if every siphon includes an initially marked trap. This property obviously depends on the initial state. For certain combinations of structural properties, we can derive behavioural properties:

- If a PN is ORD and does not have any siphons, then it is live.
- If a PN is ORD, then STP precludes dead states.
- If a PN is ORD, ES, and the STP holds, then the net is live.
- If a PN is ORD and EFC, then it is live iff the STP holds.

*Interpretation:* Liveness ensures that none of the reactions will be blocked forever (see Section 19).

**Bad siphon**. A siphon is called bad if it does not include a trap. If there is a bad siphon, then there is no initial state to satisfy the STP [HMS10].

*Interpretation:* Without appropriate timing constraints, a bad siphon may always become clean, then blocking reactions which have a precursor in the bad siphon.

*Examples:* The place set {s1, s2} forms a (bad) siphon, and the place set {s3, s4} a trap. The siphon holds initially one token, which can circulate via the reactions r1 and r2, until the reaction r3 moves the token from s2 to s3, which cleans the siphon, and there will never a token enter this cycle again, while the trap gets a token, which now can circulate forever via the reactions r4 and r5.

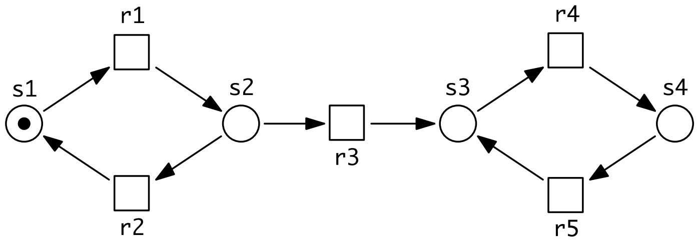


Figure S19. A Petri net illustrating siphon and trap.

## 22. Snoopy - Charlie interplay

Some analysis results computed with Charlie can be visualised with Snoopy, such as (a) transition invariants and (b) place invariants, or siphons/traps; see Section 21 for explanations of all these terms. To make use of this feature, follow these steps:

**In Charlie:**

(1) Switch on options -> 'export invariants' or 'export traps/siphons', and specify appropriate directories (see Section 18).

(2) Compute the results.

Now, there should be new files with the default extensions 'inv' or 'res' in the specified directories.

**Go to Snoopy:**

(1) Open the corresponding Petri net.

(2) Go to Extras -> Load node set file, and choose the result file written by Charlie.

(3) A new window appears, as shown in (a) and (b), where a single node set or the union/intersection of node sets can be highlighted according to the chosen colour.

To assign to each invariant a different colour, do:

(1) Go to Extras -> Load node set file.

(2) Select a colour and a node set.

(3) Select 'keep coloring'.

(4) Close the window showing all node sets.

(5) Save the Petri net file.

Repeat steps 1-5 and select different colours.

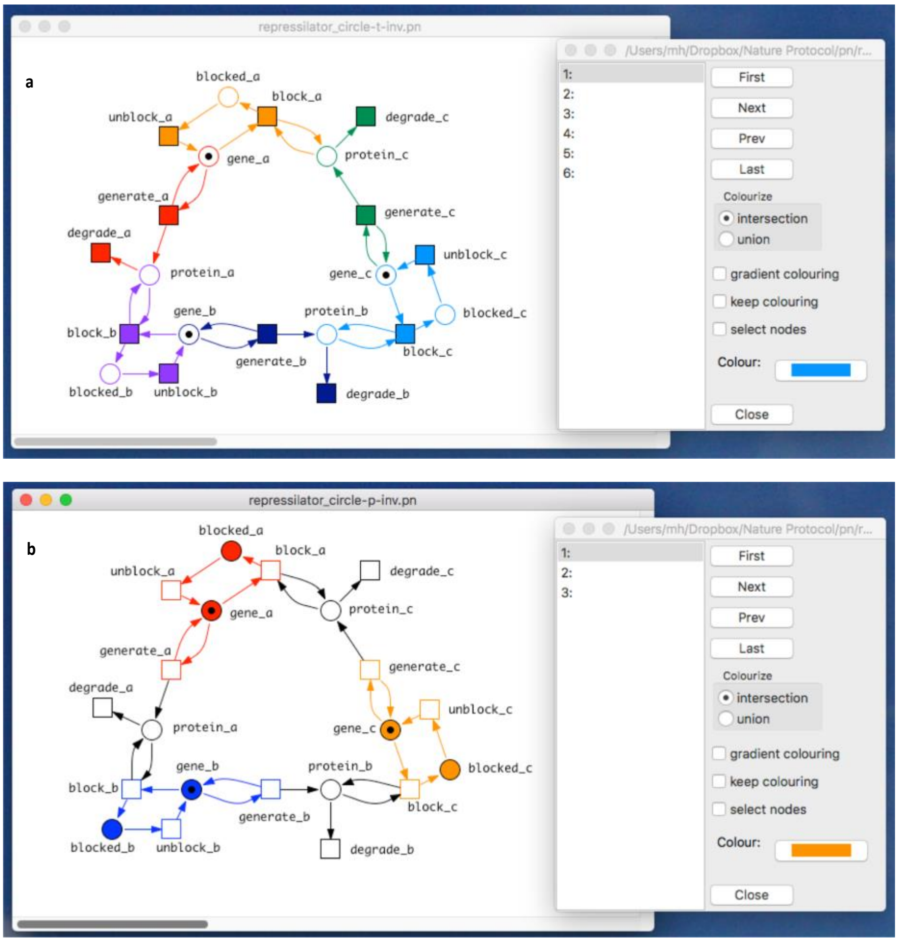


Figure S20. Snoopy - Charlie interplay.

## 23. Model checking

Model checking is a method for checking whether a model of a system meets a given specification of behavioural properties which are given in temporal logic. There are two basic principles of model checking.

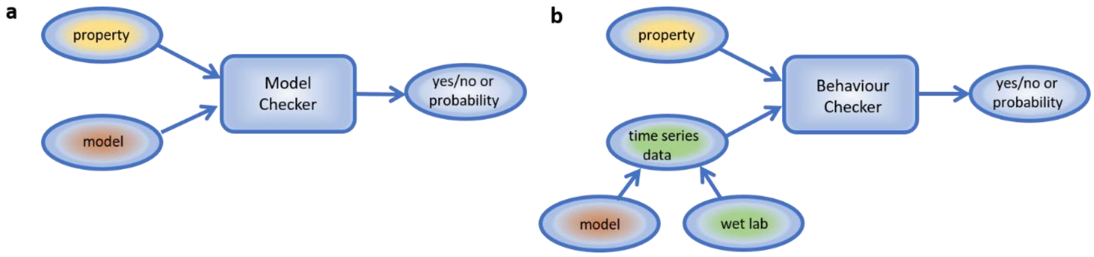


Figure S21. Model checking.

**(a)** *Analytical model checking* -- the entire state space is checked without simulating the model.

**(b)** *Simulative model checking* -- a model is simulated and the time series traces are checked. Alternatively, time series traces can be generated by observations of the real (biological) system, enabling lab data to be compared with the model's behaviour; in general (**b**) can be termed

*behavioural checking*. In our platform we are able to check the behavioural properties of both biochemical entities (represented by places) and reaction activities (represented by transitions).

Analytical model checking is limited by size of the state space, whereas simulative model checking is inherently an approximative approach because checking is performed over a finite number of finite simulation traces. In our platform, analytical model checking is supported for QPN and SPN, while simulative model checking is supported in our platform for all quantitative net classes, including all fuzzy classes. Further, *in-line* simulative model checking, where the properties are checked during the generation of the traces, is supported for SPN by Marcie, and *off-line* model checking where the traces (from the model or the wet lab) are first generated before checking, is supported for all quantitative net classes by MC2. In the following we confine ourselves to simulative model checking.

Checking stochastic systems requires a stochastic temporal logic, and thus conformance to properties is given in terms of probabilities, whereas deterministic systems do not involve a stochastic element and thus conformance is expressed in a yes/no manner. In practice both types of systems can be checked with the same stochastic logic, where the deterministic results are given as 0 or 1 probabilities, whilst the stochastic checks result in the range 0 to 1. The result is approximated by the ratio of the number of traces that conform to the property over the total number of traces checked.

Our tools employ a linear time probabilistic temporal logic with constraints, PLTLc [DG08]. The operators and functions of PLTLc include

- the usual first order logic connectives: & (And), ∨ (Or), ¬ (Not), → (Implication),
- temporal operator: F(φ) (φ holds eventually (finally)),
  *The concentration of metabolite A will at some point be below a certain threshold (100)*:
  $P_{\geq 1}$[ F($A$ < 100) ],
- temporal operator: G(φ) (φ holds forever (globally)),
  *The concentration of metabolite A is always below a certain threshold (100)*:
  $P_{\geq 1}$[ G($A$ < 100) ],
- temporal operator: φ1 *U* φ2 (φ1 holds until φ2 holds),
  *The concentration of metabolite A is below a certain threshold (100) until the concentration of metabolite B is above another threshold (200)*:
  $P_{\geq 1}$[ ($A$ < 100) U ($B$ > 200) ],
- function *d* returning the derivative of the trace variable at each time point,
  *The concentration of metabolite A is always strictly increasing*:
  $P_{\geq 1}$[ G(d($A$) > 0 ) ].

More details and further examples can be found in:

- [DG08] – model checking used for optimisation of parameters,
- [PG16] – multiscale spatio-temporal model checking,
- [MMG+12] – automatic generation of properties from sets of traces,
- [GHJ+19] – exploring whole genome metabolic models generated from SBML, using a library of general PLTLc properties to check very large numbers of places and transitions,
- [GHG+19] – model validation using the PLTLc library of general properties.

The library of general PLTLc properties will be available on github, together with example scripts.

## 24. Data analytics

The simulation output comprises time series traces of the values of place markings or transition activity, which can be saved in the form of CSV (or equivalent) files. These data can then be analysed with standard time-series data analytic techniques using software such as Matlab or R. The actual methods applied depend on the problem being investigated, but common techniques include:

**Feature extraction**, e.g.

- Peaks & troughs: detection, maximum/minimum values, numbers of occurrence, and orders of occurrence,
- Oscillations: detection, frequency, damping.

**Unsupervised learning**:

- Visual analytics – display of data in a manner to support visual inspection of the traces and visual feature extraction
- Clustering
    - Identify an appropriate distance/similarity metric (Euclidean, Dynamic Time Warping (DTW))
    - Perform pairwise comparison using the similarity measure
    - Use existing clustering techniques such as hierarchical clustering, k-means, or density-based clustering, to find clustering structures. These can be displayed as dendrograms for hierarchical clustering or scatter plots for k-means and density-based.

**Supervised learning**: For time series classification, first extract and build features from time series data, and then apply existing classification techniques to the feature set such as support vector machines, k-nearest neighbours, neural networks, regression or decision trees.

Library packages from CRAN in R which can be used include:

- General approaches:
    - **timetk** - A Tool Kit for Working with Time Series in R - Easy visualization, wrangling, and feature engineering of time series data for forecasting and machine learning prediction
    - **distantia** to assess the dissimilarity between multivariate time-series.
    - **Plot3d, ggplot2, dygraphs** - visualizing (comparing) time series data
    - **TSrepr** - Methods for representations (i.e. dimensionality reduction, pre-processing, feature extraction) of time series
    - **TSMining** - Repeated Pattern (motif) discovery in time-series data
- Time series clustering
    - **TSdist** - Distance Measures for Time Series Data
    - **dtwclust** - Time Series Clustering Along with Optimizations for the Dynamic Time Warping Distance, including **dtw** to compute and visualize dynamic time warping alignments

    - **factoextra, stats** - k-means clustering
    - **stats** - hierarchical clustering including **dist(), hclust(), kmeans()** functions
    - **fpc, dbscan** - density-based clustering (DBSCAN)
- Classification
    - **wavelets -** Contains functions for computing and plotting Discrete Wavelet Transform (DWT)
- Feature selection
    - **StepAIC** (in the MASS package)
    - **Boruta**, based on random forests

Illustrations of the use of these techniques can be found in these case studies:

- **Multiscale modelling and analysis of planar cell polarity in the *Drosophila* wing:** unsupervised learning with DBscan and Principal Component analysis was used to generate clusters of the behaviour of cells in a 2D tissue, and a pattern mining approach was applied to generate high-level classificatory descriptions in temporal logic of the behaviour of the clusters [GLG+11], [GGH+13], [MMG+12].
- **Dynamic genome scale metabolic models, applied to E. *coli* K-12:** simulative model checking was performed using temporal logic property libraries, clustering and data analysis, over time series of reaction rates and metabolite concentrations. This approach was extended to consider the evolution of reaction-oriented properties of subnets over time, including dead subnets and functional subsystems [GHJ+19]**.**
- **Derivation of a proxy for the biomass function in whole genome scale models:** a dynamic biomass function was developed which acts as a faithful proxy for the FBA equivalent, applied to E. *coli* K-12. This involved complexity reduction using StepAIC to remove excess correlated variables identified using clustering, combined with machine learning using step-wise regression analysis employing the Boruta package [SGH18].

## 25. Tools of the Petri net platform to support the workflow

Table S3 - Tools of the Petri net platform to support the workflow.

| Name | Description |
|---|---|
| Snoopy [HHL+12]<br><br>Manual ColPN [LHR12]<br>Manual ColHPN [HLR+17a]<br>CANDL report [ACR+20] | Hierarchical (coloured) Petri net editor and simulator supporting various types of Petri nets, including all Petri net classes used in this protocol, with an automatic conversion between them. Snoopy supports several data exchange formats, among them the Systems Biology Markup Language (SBML, level 1 and 2). For communication within the PetriNuts platform, Snoopy reads and writes ANDL and CANDL files, and writes simulation traces as CSV files. Snoopy's proprietary file format uses XML technology; default file extensions indicate the net class (i.e., pn, xpn, spn, cpn, hpn, fspn, fcpn, fhpn, colpn, …, colfhpn). A Snoopy2LATEX generator supports documentation [Sha15]. |

| | |
|---|---|
| Patty [Sch08] | JavaScript to support Petri net animation (token flow) in a web browser; does not require any installation on the user's site. Patty reads PN and XPN files in Snoopy's proprietary format. |
| Charlie [HSW15] | Analysis of (uncoloured) Petri net models applying standard techniques of Petri net theory, complemented by explicit CTL and LTL model checking. Charlie reads ANDL files, and writes some analysis results (invariants, siphons, traps) into files, to be read by Snoopy for visualisation; for details see [BHM11]. |
| Marcie [SHR11]<br><br>Marcie Manual [SRH17] | Model checker for Petri nets and (generalized) stochastic Petri nets, and their coloured counterparts; it combines exact analysis techniques gaining their efficiency by symbolic data structures (IDD) with approximative analysis techniques building on fast adaptive uniformization (FAU) and parallelized stochastic simulation (Gillespie, tau leaping, delta leaping). It supports CTL, CSL and PLTLc model checking. Marcie reads ANDL and CANDL files. |
| Spike [CH19] | Command line tool for reproducible stochastic, continuous & hybrid simulation experiments of large-scale (coloured) Petri nets. Spike reads a couple of file formats, among them are SBML, ANDL and CANDL. |
| MC2 [DG08] | A Monte Carlo Model Checker for LTLc and PLTLc, operating on stochastic, deterministic, and hybrid simulation traces or even wetlab data, given as CSV files. We use MC2 to analyze deterministic and hybrid traces generated by Snoopy or Spike. |
| Obtain from | http://www-dssz.informatik.tu-cottbus.de/DSSZ/Software |
| **Third party tools** | |
| R routines | Analysis of simulation data using techniques such as clustering.<br>https://www.r-project.org |
| Gnuplot routines | Visualisation of simulation results as 2D/3D space.<br>http://www.gnuplot.info/ |
| Matlab routines | Visualisation of simulation results as 2D space.<br>https://www.mathworks.com/<br>https://figshare.com/articles/dataset/Delta_Models/5522020 as used in [LYS+22]. |
| Python routines | Visualisation of simulation results as 2D space.<br>https://www.python.org/ |

## 26. Selected case studies undertaken with the platform

Table S4. Selected case studies undertaken with the platform.

| Category | Uncoloured Petri nets | Coloured Petri nets |
|---|---|---|
| Gene transcription & regulatory networks | • Lac operon/prokaryotic gene regulation (SPN) [HLG+09]<br>• Hypoxia Response Network (QPN, CPN) [HS10]<br>• phosphate regulation in enteric bacteria (SPN) [MRH12]<br>• probing the Waddington landscape [RWH+20] | |
| Signal transduction networks | • Apoptosis (QPN) [HKW04]<br>• Mating pheromone response pathway in Saccharomyces cerevisiae (QPN) [SHK06]<br>• Mitogen-Activated Protein Kinase (MAPK) (QPN, SPN, CPN) [HGD08]<br>• RKIP-inhibited ERK pathway (QPN, SPN, CPN) [HDG10]<br>• Circadian oscillation (HPN) [HH12]<br>• JAK/STAT signaling (QPN) [BDR+13]<br>• Eukaryotic cell cycle (HPN) [HSH13]<br>• Yeast pheromone pathway, (QPN, SPN) [SHK06], [MSD+14]<br>• Tobacco-induced prothrombotic state, inflammation and oxidative stress (QPN) [GFF19] | • Planar cell polarity in *Drosophila* wing (ColCPN) [GGH+13]<br>• Coupled Ca2+ channels at multiple spatial scales (ColSPN) [LH13]<br>• Spatial patterning in C. elegans vulval development (ColSPN/ColCPN) [LHY14]<br>• Brusselator/Pattern formation (ColSPN, ColCPN) [LBH+14]<br>• Yeast cell cycles based on multisite phosphorylation (ColHPN) [HLH18]<br>• Calcium dynamics inside a dendritic spine (ColHPN) [HLR+18]<br>• Intracellular calcium diffusion (ColHPN) 2D: [IHH19], 3D: [IHA+20] |
| Metabolic networks | • Sucrose breakdown in potato tuber (QPN) [KJH05]<br>• Combined glycolysis and pentose phosphate pathway in erythrocytes (QPN) [KH08]<br>• Whole-genome metabolism of *Escherichia coli* K-12 under different growth conditions (SPN) [GHJ+19]<br>• Liver iron metabolism (HPN) [HH18a]<br>• Asparagine synthetases from wheat (CPN) [XCP+18] | |

| | | |
|---|---|---|
| | • adaptive immune response system against HCV infection (CPN) [ONI+18]<br>• Akt-Wnt-mTOR-p70S6K signalling network in neurons (HPN) [FH18] | |
| Combinations of the above | • Von Hippel-Lindau (VHL) Tumor Suppressor Interaction (QPN) [MPG+14]<br>• Hypoxia Pathway to predict a novel incoherent feed-forward loop controlling SDF-1 expression in acute kidney injury (SPN) [HGM+16]<br>• Angiogenesis (QPN) [FRZ+17]<br>• Competence cycle in the human pathogen Streptococcus pneumoniae (QPN, CPN) [WPB+18] | • Innate immune response to Mycobacterium infection in zebrafish (ColQPN, nested hierarchies encode four scales) [CKM+12], [CHK+15]<br>• Circadian oscillation (QPN, SPN, CPN, HPN, coloured counterparts) [BHM15]<br>• Spatial quorum sensing (ColHPN) [GHG+19]<br>• Cell-fate decision and pattern formation in *Drosophila* large intestine by Delta/Notch signalling (ColCPN) [LYS+19], [LYS+22] |
| Population level | • Chagas disease transmission (HPN) [HH15]<br>• Feeding regimen induced entrainment of Hepatocyte circadian oscillators (HPN) [TA15]<br>• A trophic network of the Venice lagoon (QPN, CPN) [BBB+18]<br>• Stage model of honey bee population development (HPN) [HH18a] | • Bacterial colonies with phase variable genes (ColSPN) [GHL+13], [PGH+15]<br>• humoral immune system response (ColSPN) [PCM+16] |
| Synthetic biology | • Biosensor (SPN, CPN) [GHR+08], [GTR+10] | • DNA walker circuit design (ColSPN) [GHR18] |
| Model construction by module composition | • A structured approach for the engineering of biochemical network models [BGH+08]<br>• Target driven biochemical network reconstruction [WGG10] | • Model construction by the composition of two modules (ColCPN) [GHG+19]<br>• component-based extension of basic SIR models to include spatio-geographic information [CGH22] |

## 27. Unfolding data

For illustration, we provide some figures for a few selected case studies.

Table S5. A few selected case studies.

| Case study | Size of coloured model | Size of uncoloured model |
|---|---|---|
| Planar cell polarity in *Drosophila* wing (ColCPN) [GGH+13] | Tissue with 800 cells: | |
| | 16 places,<br>19 transitions,<br>45 arcs,<br>49,600 tokens | 135,200 places,<br>175,286 transitions,<br>445,792 arcs |
| Phase variation in bacterial colony growth (ColSPN) [GHL+13], [PGH+15] | Growth up to colony size 70,000,000 on 101×101 grid: | |
| | 4 places,<br>3 transitions,<br>12 arcs,<br>71,407,001 tokens | 30,605 places,<br>362,405 transitions,<br>1,812,022 arcs |
| DNA walker (ColSPN) [GHR18] | optimised layout on 25×33 grid: | |
| | 2 places,<br>9 transitions,<br>17 arcs | 77 places,<br>536 transitions,<br>1,498 arcs |
| Diffusion 3D26, basic module (ColCPN) [GHG+19], supplementary material | 11×11 grid: | |
| | 1 place,<br>1 transition,<br>2 arcs | 13,31 places,<br>7,260 transitions,<br>14,520 arcs |
| Quorum sensing (ColHPN) [GHG+19] | 25 bacteria positioned on 101×101 grid: | |
| | 34 places,<br>40 transitions,<br>102 arcs | 11,026 places,<br>81,375 transitions,<br>163,300 arcs |
| Cell-fate decision and pattern formation in *Drosophila* large intestine by Delta/Notch signalling (ColCPN) [LYS+19], [LYS+22] | Tissue with 144 cells: | |
| | 16 places,<br>19 transitions,<br>29 arcs | 1,440 places,<br>3,024 transitions,<br>6,336 arcs |
| 3D diffusion (ColHPN3) [IHA+20] | four clusters, each containing 12 channels: | |
| | 12 places,<br>24 transitions | 3,212 places,<br>34,462 transitions |
| Pandemic SIR model with 10 age strata (ColSPN, ColCPN) [CGH22] | 48 countries (Europe) | |
| | 3 places,<br>5 transitions,<br>11 arcs,<br>4,800,000 tokens | 1,440 places,<br>10,380 transitions,<br>30,360 arcs,<br>4,800,000 tokens |

## 28. Abbreviations

Table S6. Abbreviations.

| | |
|---|---|
| **Petri net classes (ordered by functionality)** | |
| PN | Petri net |
| QPN | Qualitative Petri net (to emphasise absence of time) |
| XPN | Extended Petri net |
| SPN | Stochastic Petri net |
| CPN | Continuous Petri net |
| HPN | Hybrid Petri net |
| ColPN | Coloured Petri net |
| ColQPN | Coloured qualitative Petri net net (to emphasise absence of time) |
| ColXPN | Coloured extended Petri net |
| ColSPN | Coloured stochastic Petri net |
| ColCPN | Coloured continuous Petri net |
| ColHPN | Coloured hybrid Petri net |
| FSPN | Fuzzy stochastic Petri net |
| FCPN | Fuzzy continuous Petri net |
| FHPN | Fuzzy hybrid Petri net |
| ColFSPN | Coloured fuzzy stochastic Petri net |
| ColFCPN | Coloured fuzzy continuous Petri net |
| ColFHPN | Coloured fuzzy hybrid Petri net |
| **General abbreviations (ordered alphabetically)** | |
| ANDL | Abstract Net Definition Language |
| BNF | Backus Naur Form |
| CANDL | Coloured Abstract Net Definition Language |
| CTL | Computational tree logic |
| CTMC | Continuous time Markov chain |
| CSP | Constraint satisfaction problem |
| CSRL | Continuous stochastic reward logic |
| CSV | Comma-separated values |
| GUI | Graphical user interface |
| IDD | Interval decision diagram |
| LTL | Linear-time logic |
| LTLc | Linear-time logic with constraints |
| LTS | Labelled transition system |
| ODEs | System of ordinary differential equations |
| PLTLc | Probabilistic linear-time logic with constraints |
| PO | Partial order semantics |
| SPC | Spike configuration |
| SSA | Stochastic simulation algorithm |
| STP | Siphon Trap Property |

| | |
|---|---|
| 1D | One dimensional |
| 2D | Two dimensional |
| 3D | Three dimensional |
| **Abbreviations used by Charlie (ordered by Charlie result vector)** | |
| PUR | pure |
| ORD | ordinary |
| HOM | homogeneous |
| CSV | conservative |
| SCF | static conflict free |
| CON | connected |
| SC | strongly connected |
| FT0 | a transition without pre-places |
| TF0 | a transition without post-places |
| FP0 | a place without pre-transitions |
| PF0 | a place without post-transitions |
| NC | net structure class |
| SM | state machine |
| SG | synchronisation graph |
| FC | free choice |
| ECF | extended free choice |
| ES | extended simple |
| k-B | k-bounded |
| B | bounded |
| LIV | liveness |
| REV | reversibility |
| DSt | dead state |
| DCF | dynamic conflict free |
| CPI | covered with P-invariants |
| CTI | covered with T-invariants |
| STP | siphon-trap property |

## References


[ABC+98] Marsan, M.A., Balbo, G., Conte, G., Donatelli, S., and Franceschinis, G. (1998). Modelling with Generalized Stochastic Petri Nets. ACM SIGMETRICS Perform. Eval. Rev. *26*, 2. 10.1145/288197.581193.

[ACR+20] Assaf, G., Chodak, J., Rohr, C., Schwarick, M., and Heiner, M. (2020) CANDL Report; Department of Computer Science, Brandenburg University of Technology Cottbus.

[AF73] Agerwala, T., and Flynn, M. (1973). Comments on capabilities, limitations and "correctness" of Petri nets. SIGARCH Comput. Archit. News *2*, 81–86. 10.1145/633642.803973.

[AHL19] Assaf, G., Heiner, M., and Liu, F. (2019). Biochemical Reaction Networks with Fuzzy Kinetic Parameters in Snoopy. In Computational Methods in Systems Biology Lecture Notes in Computer Science., L. Bortolussi and G. Sanguinetti, eds. (Springer International Publishing), pp. 302–307. 10.1007/978-3-030-31304-3_17.

[AHL21] Assaf, G., Heiner, M., and Liu, F. (2021). Colouring fuzziness for systems biology. Theoretical Computer Science *875*, 52–64. 10.1016/j.tcs.2021.04.011.

[BBB+18] Baldan, P., Bocci, M., Brigolin, D., Cocco, N., Heiner, M., Simeoni, M. (2018). Petri Nets for Modelling and Analysing Trophic Networks. Fundam. Inf. *160*, 27–52. 10.3233/FI-2018-1673.

[BCP08] Blossey, R., Cardelli, L., and Phillips, A. (2008). Compositionality, stochasticity, and cooperativity in dynamic models of gene regulation. HFSP Journal *2*, 17–28. 10.2976/1.2804749.

[BDR+13] Blätke, M.A., Dittrich, A., Rohr, C., Heiner, M., Schaper, F., and Marwan, W. (2013). JAK/STAT signalling – an executable model assembled from molecule-centred modules demonstrating a module-oriented database concept for systems and synthetic biology. Mol. BioSyst. *9*, 1290–1307. 10.1039/C3MB25593J.

[BGH+08] Breitling, R., Gilbert, D., Heiner, M., and Orton, R. (2008). A structured approach for the engineering of biochemical network models, illustrated for signalling pathways. Brief Bioinform *9*, 404–421. 10.1093/bib/bbn026.

[BHM11] Blätke, M.-A., Heiner, M., and Marwan, W. (2011). Tutorial - Petri Nets in Systems Biology (Otto von Guericke University Magdeburg, Magdeburg Centre for Systems Biology).

[BHM15] Blätke, M.A., Heiner, M., and Marwan, W. (2015). BioModel Engineering with Petri Nets. In Algebraic and Discrete Mathematical Methods for Modern Biology, R. S. Robeva, ed. (Academic Press), pp. 141–192. 10.1016/B978-0-12-801213-0.00007-1.

[CH19] Chodak, J. and Heiner, M. (2019). Reproducible Simulation Experiments with Configuration File Branching. In Computational Methods in Systems Biology: 17th International Conference, CMSB 2019, Trieste, Italy, September 18–20, 2019, Proceedings (Springer-Verlag), pp. 315–321. 10.1007/978-3-030-31304-3_19.

[CHK+15] Carvalho, R.V., van den Heuvel, J., Kleijn, J., and Verbeek, F.J. (2015) Coupling of Petri net models of the mycobacterial infection process and innate immune response. *Computation*. **3**(2), 150-176. 10.3390/computation3020150

[CKM+12] Carvalho, R.V., Kleijn, J., Meijer, A.H., and Verbeek, F.J. (2012). Modeling Innate Immune Response to Early *Mycobacterium* Infection. Computational and Mathematical Methods in Medicine *2012*, e790482. 10.1155/2012/790482.

[CMM+96] Collier, J.R., Monk, N.A., Maini, P.K., and Lewis, J.H. (1996). Pattern formation by lateral inhibition with feedback: a mathematical model of delta-notch intercellular signalling. J Theor Biol *183*, 429–446. 10.1006/jtbi.1996.0233.

[CGH22] Connolly, S., Gilbert, D., and Heiner, M. (2022). From Epidemic to Pandemic Modelling; Frontiers in Systems Biology. 10.3389/fsysb.2022.861562

[DA10] David, R. and Alla, H. (2010). Discrete, Continuous, and Hybrid Petri Nets (Springer).

[DFS98] Dufourd, C., Finkel, A., and Schnoebelen, Ph. (1998). Reset nets between decidability and undecidability. In Automata, Languages and Programming Lecture Notes in Computer Science., K. G. Larsen, S. Skyum, and G. Winskel, eds. (Springer), pp. 103–115. 10.1007/BFb0055044.

[DG08] Donaldson, R. and Gilbert, D. (2008). A Model Checking Approach to the Parameter Estimation of Biochemical Pathways. In Computational Methods in Systems Biology Lecture Notes in Computer Science., M. Heiner and A. M. Uhrmacher, eds. (Springer), pp. 269–287. 10.1007/978-3-540-88562-7_20.

[FH18] Fortin, M.P. and Hardy, S.V. (2018) A hybrid Petri net model of the Akt-Wnt-mTOR-p70S6K signalling network in neurons. Fund. Inform. 160(1-2), 1-25. 10.3233/FI-2018-1672

[FRZ+17] Formanowicz, D., Radom, M., Zawierucha, P., and Formanowicz, P. (2017). Petri net-based approach to modeling and analysis of selected aspects of the molecular regulation of angiogenesis. PLoS One *12*, e0173020. 10.1371/journal.pone.0173020.

[GFF19] Gutowska, K., Formanowicz, D., and Formanowicz, P. (2019). Selected Aspects of Tobacco-Induced Prothrombotic State, Inflammation and Oxidative Stress: Modeled and Analyzed Using Petri Nets. Interdiscip Sci *11*, 373–386. 10.1007/s12539-018-0310-7.

[GGH+13] Gao, Q., Gilbert, D., Heiner, M., Liu, F., Maccagnola, D., and Tree, D. (2013). Multiscale Modeling and Analysis of Planar Cell Polarity in the Drosophila Wing. IEEE/ACM Transactions on Computational Biology and Bioinformatics *10*, 337–351. 10.1109/TCBB.2012.101.

[GHG+19] Gilbert, D., Heiner, M., Ghanbar, L., and Chodak, J. (2019). Spatial quorum sensing modelling using coloured hybrid Petri nets and simulative model checking. BMC Bioinformatics. *20*, 173. 10.1186/s12859-019-2690-z.

[GHJ+19] Gilbert, D., Heiner, M., Jayaweera, Y., and Rohr, C. (2019). Towards dynamic genome scale models. Brief. Bioinform. *20*, 1167-1180. 10.1093/bib/bbx096.

[GHL+13] Gilbert, D., Heiner, M., Liu, F., and Saunders, N. (2013). Colouring Space - A Coloured Framework for Spatial Modelling in Systems Biology. In Application and Theory of Petri Nets and Concurrency Lecture Notes in Computer Science., J.-M. Colom and J. Desel, eds. (Springer), pp. 230–249. 10.1007/978-3-642-38697-8_13.

[GHR+08] Gilbert, D., Heiner, M., Rosser, S., Fulton, R., Gu, X., and Trybilo, M. (2008). A Case Study in Model-driven Synthetic Biology. In Biologically-Inspired Collaborative Computing IFIP – The International Federation for Information Processing., M. Hinchey, A. Pagnoni, F. J. Rammig, and H. Schmeck, eds. (Springer US), pp. 163–175. 10.1007/978-0-387-09655-1_15.

[GHR18] Gilbert, D., Heiner, M., and Rohr, C. (2018). Petri-net-based 2D design of DNA walker circuits. Nat Comput *17*, 161–182. 10.1007/s11047-018-9671-4.

[GLG+11] Gao, Q., Liu, F., Gilbert, D., Heiner, M., and Tree, D. (2011). A multiscale approach to modelling planar cell polarity in Drosophila wing using hierarchically coloured Petri nets. In Proceedings of the 9th International Conference on Computational Methods in Systems Biology CMSB '11. (Association for Computing Machinery), pp. 209–218. 10.1145/2037509.2037538.

[GTR+10] Gu, X., Trybiło, M., Ramsay, S., Jensen, M., Fulton, R., Rosser, S., and Gilbert, D. (2010). Engineering a novel self-powering electrochemical biosensor. Syst Synth Biol *4*, 203–214. 10.1007/s11693-010-9063-2.

[HDG10] Heiner, M., Donaldson, R., and Gilbert, D. (2010). Petri Nets for Systems Biology. In Symbolic Systems Biology: Theory and Methods (Jones & Bartlett Learning, LCC), pp. 61–97.

[HFS+03] Hucka, M., Finney, A., Sauro, H.M., Bolouri, H., Doyle, J.C., Kitano, H., Arkin, A.P., Bornstein, B.J., Bray, D., Cornish-Bowden, A., et al. (2003). The systems biology markup language (SBML): a medium for representation and exchange of biochemical network models. Bioinformatics *19*, 524–531. 10.1093/bioinformatics/btg015.

[HG13] Heiner, M. and Gilbert, D. (2013). BioModel engineering for multiscale systems biology. Prog. Biophys. Mol. Bio. *111*, 119-128. 10.1016/j.pbiomolbio.2012.10.001.

[HGD08] Heiner, M., Gilbert, D., and Donaldson, R. (2008). Petri Nets for Systems and Synthetic Biology. In Formal Methods for Computational Systems Biology Lecture Notes in Computer Science., M. Bernardo, P. Degano, and G. Zavattaro, eds. (Springer), pp. 215–264. 10.1007/978-3-540-68894-5_7.

[HGM+16] Heidary, Z., Ghaisari, J., Moein, S., Naderi, M., and Gheisari*, Y. (2016). Stochastic Petri Net Modeling of Hypoxia Pathway Predicts a Novel Incoherent Feed-Forward Loop Controlling SDF-1 Expression in Acute Kidney Injury. IEEE Transactions on NanoBioscience *15*, 19–26. 10.1109/TNB.2015.2509475.

[HH12] Herajy, M. and Heiner, M. (2012). Hybrid representation and simulation of stiff biochemical networks. Nonlinear Analysis: Hybrid Systems *6*, 942–959. 10.1016/j.nahs.2012.05.004.

[HH15] Herajy, M. and Heiner, M. (2015). Modeling and simulation of multi-scale environmental systems with Generalized Hybrid Petri Nets. Frontiers in Environmental Science 3. 10.3389/fenvs.2015.00053

[HH18a] Herajy, M. and Heiner, M. (2018). Adaptive and Bio-semantics of Continuous Petri Nets: Choosing the Appropriate Interpretation. Fundam. Inf. *160*, 53–80. 10.3233/FI-2018-1674.

[HHL+12] Heiner, M., Herajy, M., Liu, F., Rohr, C., and Schwarick, M. (2012). Snoopy – A Unifying Petri Net Tool. In Application and Theory of Petri Nets Lecture Notes in Computer Science., S. Haddad and L. Pomello, eds. (Springer), pp. 398–407. 10.1007/978-3-642-31131-4_22.

[HKW04] Heiner, M., Koch, I., and Will, J. (2004). Model validation of biological pathways using Petri nets—demonstrated for apoptosis. Biosystems *75*, 15–28. 10.1016/j.biosystems.2004.03.003.

[HLG+09] Heiner, M., Lehrack, S., Gilbert, D., and Marwan, W. (2009). Extended Stochastic Petri Nets for Model-Based Design of Wetlab Experiments. In Transactions on Computational Systems Biology XI Lecture Notes in Computer Science., C. Priami, R.-J. Back, and I. Petre, eds. (Springer), pp. 138–163. 10.1007/978-3-642-04186-0_7.

[HLH18] Herajy, M., Liu, F., and Heiner, M. (2018). Efficient modelling of yeast cell cycles based on multisite phosphorylation using coloured hybrid Petri nets with marking-dependent arc weights. Nonlinear Analysis: Hybrid Systems *27*, 191–212. 10.1016/j.nahs.2017.09.002.

[HLR+17a] Herajy, M., Liu, F., Rohr, C., and Heiner, M. (2017). Coloured Hybrid Petri Nets in Snoopy - User Manual (Department of Computer Science, Brandenburg University of Technology Cottbus).

[HLR+17b] Herajy, M., Liu, F., Rohr, C., and Heiner, M. (2017). Snoopy's hybrid simulator: a tool to construct and simulate hybrid biological models. BMC Systems Biology *11*, 71. 10.1186/s12918-017-0449-6.

[HLR+18] Herajy, M., Liu, F., Rohr, C., and Heiner, M. (2018). Coloured Hybrid Petri Nets: An adaptable modelling approach for multi-scale biological networks. Computational Biology and Chemistry *76*, 87–100. 10.1016/j.compbiolchem.2018.05.023.

[HMS10] Heiner, M., Mahulea, C., and Silva, M. (2010). On the importance of the deadlock trap property for monotonic liveness. In Recent Advances in Petri Nets and Concurrency, RAPNeC 2010 - Workshops of the 31st International Conference on Application and Theory of Petri Nets and Other Models of Concurrency, PETRI NETS 2010 and the 10th int. conf. ACSD 2010, June 21, 2010 - June 22, 2010 CEUR Workshop Proceedings. (CEUR-WS), pp. 23–38.

[HS10] Heiner, M. and Sriram, K. (2010). Structural Analysis to Determine the Core of Hypoxia Response Network. PLOS ONE *5*, e8600. 10.1371/journal.pone.0008600.

[HSH13] Herajy, M., Schwarick, M., and Heiner, M. (2013). Hybrid Petri Nets for Modelling the Eukaryotic Cell Cycle. In Transactions on Petri Nets and Other Models of Concurrency VIII Lecture Notes in Computer Science., M. Koutny, W. M. P. van der Aalst, and A. Yakovlev, eds. (Springer), pp. 123–141. 10.1007/978-3-642-40465-8_7.

[HSW15] Heiner M., Schwarick M., and Wegener J. (2015) Charlie—an extensible Petri net analysis tool. in: *Application and Theory of Petri Nets and Concurrency*, Vol. LNCS 9115 (eds. Devillers, R. & Valmari, A.) 200-211 (Springer, Cham, Switzerland). 10.1007/978-3-319-19488-2_10

[IHA+20] Ismail, A., Herajy, M., Atlam, E., and Heiner, M. (2020). A Graphical Approach for Hybrid Simulation of 3D Diffusion Bio-Models via Coloured Hybrid Petri Nets. Modelling and Simulation in Engineering *2020*, e4715172. 10.1155/2020/4715172.

[IHH19] Ismail, A., Herajy, M., and Heiner, M. (2019). A Graphical Approach for Hybrid Modelling of Intracellular Calcium Dynamics Based on Coloured Hybrid Petri Nets. In Automated Reasoning for Systems Biology and Medicine Computational Biology., P. Liò and P. Zuliani, eds. (Springer International Publishing), pp. 349–367. 10.1007/978-3-030-17297-8_13.

[KH08] Koch, I. and Heiner, M. (2008). Petri Nets. In Analysis of Biological Networks (John Wiley & Sons), pp. 139–175.

[KJH05] Koch, I., Junker, B.H., and Heiner, M. (2005). Application of Petri net theory for modelling and validation of the sucrose breakdown pathway in the potato tuber. Bioinformatics *21*, 1219–1226. 10.1093/bioinformatics/bti145.

[Kop12] Kopan, R. (2012). Notch Signaling. Cold Spring Harb Perspect Biol *4*, a011213. 10.1101/cshperspect.a011213.

[LBH+14] Liu, F., Blätke, M.-A., Heiner, M., and Yang, M. (2014). Modelling and simulating reaction–diffusion systems using coloured Petri nets. Computers in Biology and Medicine *53*, 297–308. 10.1016/j.compbiomed.2014.07.004.

[LH13] Liu, F. and Heiner, M. (2013). Multiscale modelling of coupled Ca2+ channels using coloured stochastic Petri nets. IET Systems Biology *7*, 106–113. 10.1049/iet-syb.2012.0017.

[LH14] Liu, F. and Heiner, M. (2014). Petri Nets for Modeling and Analyzing Biochemical Reaction Networks. In Approaches in Integrative Bioinformatics: Towards the Virtual Cell, M. Chen and R. Hofestädt, eds. (Springer), pp. 245–272. 10.1007/978-3-642-41281-3_9.

[LHG18] Liu, F., Heiner, M., and Gilbert, D. (2018). Fuzzy Petri nets for modelling of uncertain biological systems. Brief Bioinform. 10.1093/bib/bby118.

[LHR12] Liu, F., Heiner, M., and Rohr, C. (2012). Manual for colored Petri nets in Snoopy (Department of Computer Science, Brandenburg University of Technology Cottbus).

[LHY14] Liu, F., Heiner, M., and Yang, M. (2014). Modeling and analyzing biological systems using coloured hierarchical Petri nets, illustrated by C. elegans vulval development. WSPC J. Biol. Syst. *22*, 463-493. 10.1142/S0218339014500181.

[LYS+19] Liu, F., Yamamoto, E., Shirahama, K., Saitoh, T., Aoyama, S., Harada, Y., Murakami, R., and Matsuno, H. (2019). Pattern formation analysis by colored Petri nets with quantitative manipulation of a gene. In 2019 IEEE International Conference on Bioinformatics and Biomedicine (BIBM), pp. 138–144. 10.1109/BIBM47256.2019.8983053.

[LYS+22] Liu, F., Yamamoto, E., Shirahama, K., Saitoh, T., Aoyama, S., Harada, Y., Murakami, R., and Matsuno, H. (2022). Analysis of Pattern Formation by Colored Petri Nets With Quantitative Regulation of Gene Expression Level. IEEE/ACM Trans Comput Biol Bioinform *19*, 317–327. 10.1109/TCBB.2020.3005392.

[MMG+12] Maccagnola, D., Messina, E., Gao, Q., and Gilbert, D. (2012). A machine learning approach for generating temporal logic classifications of complex model behaviours. In Proceedings of the Winter Simulation Conference WSC '12. (Winter Simulation Conference), pp. 1–12.

[MPG+14] Minervini, G., Panizzoni, E., Giollo, M., Masiero, A., Ferrari, C., and Tosatto, S.C.E. (2014). Design and analysis of a Petri net model of the Von Hippel-Lindau (VHL) tumor suppressor interaction network. PLoS One *9*, e96986. 10.1371/journal.pone.0096986.

[MRH12] Marwan, W., Rohr, C., and Heiner, M. (2012). Petri Nets in Snoopy: A Unifying Framework for the Graphical Display, Computational Modelling, and Simulation of Bacterial Regulatory Networks. In Bacterial Molecular Networks: Methods and Protocols Methods in Molecular Biology., J. van Helden, A. Toussaint, and D. Thieffry, eds. (Springer), pp. 409–437. 10.1007/978-1-61779-361-5_21.

[MSD+14] Majumdar, A., Scott, S.D., Deogun, J.S., and Harris, S. (2014). Yeast pheromone pathway modeling using Petri nets. BMC Bioinformatics *15*, S13. 10.1186/1471-2105-15-S7-S13.

[OCJ+15] Oliveira, S.M.D., Chandraseelan, J.G., Häkkinen, A., Goncalves, N.S.M., Yli-Harja, O., Startceva, S., and Ribeiro, A.S. (2015). Single-cell kinetics of a repressilator when implemented in a single-copy plasmid. Mol. BioSyst. *11*, 1939–1945. 10.1039/C5MB00012B.

[ONI+18] Obaid, A., Naz, A., Ikram, A., Awan, F.M., Raza, A., Ahmad, J., and Ali, A. (2018). Model of the adaptive immune response system against HCV infection reveals potential immunomodulatory agents for combination therapy. Sci Rep *8*, 8874. 10.1038/s41598-018-27163-0.

[PCM+16] Pennisi, M., Cavalieri, S., Motta, S. and Pappalardo, F. (2016). A methodological approach for using high-level Petri nets to model the immune system response. BMC Bioinform. *17*, 91. 10.1186/s12859-016-1361-6

[Pet81] Peterson, J.L. (1981). Petri net theory and the modeling of systems (Prentice Hall PTR).

[PG16] Pârvu, O. and Gilbert, D. (2016). A Novel Method to Verify Multilevel Computational Models of Biological Systems Using Multiscale Spatio-Temporal Meta Model Checking. PLOS ONE *11*, e0154847. 10.1371/journal.pone.0154847.

[PGH+15] Pârvu, O., Gilbert, D., Heiner, M., Liu, F., Saunders, N., and Shaw, S. (2015). Spatial-Temporal Modelling and Analysis of Bacterial Colonies with Phase Variable Genes. ACM Trans. Model. Comput. Simul. *25*, 13:1-13:25. 10.1145/2742546.

[RWH+20] Rätzel, V., Werthmann, B., Haas, M., Strube, J., and Marwan, W. (2020). Disentangling a complex response in cell reprogramming and probing the Waddington landscape by automatic construction of Petri nets. Biosystems *189*, 104092. 10.1016/j.biosystems.2019.104092.

[Sch08] Schulz, K. (2008). An Extension of the Snoopy Software to Process and Manage Petri Net Animations (in German). Bachelor thesis, BTU Cottbus, Dep. of CS.

[SE00] Elowitz, M.B. and Leibler, S. (2000). A synthetic oscillatory network of transcriptional regulators. Nature *403*, 335–338. 10.1038/35002125.

[SGH18] Self, T., Gilbert, D., and Heiner, M. (2018). Derivation of a Biomass Proxy for Dynamic Analysis of Whole Genome Metabolic Models. In Computational Methods in Systems Biology Lecture Notes in Computer Science., M. Češka and D. Šafránek, eds. (Springer International Publishing), pp. 39–58. 10.1007/978-3-319-99429-1_3.

[Sha15] Sharma, A. (2015). Snoopy Report Generator Snoopy2LATEX (Internship report) (Department of Computer Science, Brandenburg University of Technology Cottbus).

[SHK06] Sackmann, A., Heiner, M., and Koch, I. (2006). Application of Petri net based analysis techniques to signal transduction pathways. BMC Bioinformatics *7*, 482. 10.1186/1471-2105-7-482.

[SHR11] Schwarick, M., Heiner, M., and Rohr, C. (2011). MARCIE - Model Checking and Reachability Analysis Done EffiCIEntly. In 2011 Eighth International Conference on Quantitative Evaluation of SysTems, pp. 91–100. 10.1109/QEST.2011.19.

[SLL+10] Sprinzak, D., Lakhanpal, A., Lebon, L., Santat, L.A., Fontes, M.E., Anderson, G.A., Garcia-Ojalvo, J., and Elowitz, M.B. (2010). Cis-interactions between Notch and Delta generate mutually exclusive signalling states. Nature *465*, 86–90. 10.1038/nature08959.

[SRH17] Schwarick, M., Rohr, C., and Heiner, M. (2017). MARCIE Manual (Department of Computer Science, Brandenburg University of Technology Cottbus).

[TA15] Tareen, S.H.K. and Ahmad, J. (2015). Modelling and analysis of the feeding regimen induced entrainment of hepatocyte circadian oscillators using Petri nets. PLoS One *10*, e0117519. 10.1371/journal.pone.0117519.

[TYY+02] Takashima, S., Yoshimori, H., Yamasaki, N., Matsuno, K., and Murakami, R. (2002). Cell-fate choice and boundary formation by combined action of Notch and engrailed in the Drosophila hindgut. Dev Genes Evol *212*, 534–541. 10.1007/s00427-002-0262-z.

[Val78] Valk, R. (1978). On the computational power of extended Petri nets. In Mathematical Foundations of Computer Science 1978 Lecture Notes in Computer Science., J. Winkowski, ed. (Springer), pp. 526–535. 10.1007/3-540-08921-7_101.

[WGG10] Wu, Z., Gao, Q., and Gilbert, D. (2010). Target driven biochemical network reconstruction based on Petri nets and simulated annealing. Proceedings of the 8th International Conference on Computational Methods in Systems Biology. 33-42. 10.1145/1839764.1839770

[WOQ+17] Wu, L., Ouyang, Q., and Wang, H. (2017). Robust network topologies for generating oscillations with temperature-independent periods. PLOS ONE *12*, e0171263. 10.1371/journal.pone.0171263.

[WPB+18] Weyder, M., Prudhomme, M., Bergé, M., Polard, P., and Fichant, G. (2018). Dynamic Modeling of Streptococcus pneumoniae Competence Provides Regulatory Mechanistic Insights Into Its Tight Temporal Regulation. Front Microbiol *9*, 1637. 10.3389/fmicb.2018.01637.

[XCP+18] Xu, H., Curtis, T.Y., Powers, S.J., Raffan, S., Gao, R., Huang, J., Heiner, M., Gilbert, D.R., and Halford, N.G. (2018). Genomic, Biochemical, and Modeling Analyses of Asparagine Synthetases from Wheat. Front Plant Sci *8*, 2237. 10.3389/fpls.2017.02237.

[ZOS03] Zevedei-Oancea, I. and Schuster, S. (2003). Topological analysis of metabolic networks based on Petri net theory. In Silico Biol *3*, 323–345.
